# Supplementary material for: Potent SARS-CoV-2 neutralizing antibodies with protective efficacy against newly emerged mutational variants
Source: Nat Commun. 2021 Nov 2;12:6304. doi: 10.1038/s41467-021-26539-7 (PMC8563728; doi:10.1038/s41467-021-26539-7)
Supplement: Supplementary file 1 — Supplementary Information File [file 41467_2021_26539_MOESM1_ESM.pdf]

Supplementary Information

Potent SARS-CoV-2 neutralizing antibodies with protective efficacy against newly emerged mutational variants

Tingting Li[1,2,*], Xiaojian Han[1,2,*], Chenjian Gu[3,*], Hangtian Guo[4,5,*], Huajun Zhang[6,*], Yingming Wang[1,2,*], Chao Hu[1,2], Kai Wang[7], Fengjiang Liu[4], Feiyang Luo[1,2], Yanan Zhang[8,9], Jie Hu[7], Wang Wang[1,2], Shenglong Li[1,2], Yanan Hao[1,2], Meiying Shen[10], Jingjing Huang[1,2], Yingyi Long[1,2], Shuyi Song[1,2], Ruixin Wu[1,2], Song Mu[1,2], Qian Chen[1,2], Fengxia Gao[1,2], Jianwei Wang[1,2], Shunhua Long[1,2], Luo Li[1,2], Yang Wu[3], Yan Gao[4], Wei Xu[3], Xia Cai[3], Di Qu[3], Zherui Zhang[8,9], Hongqing Zhang[8,9], Na Li[8,9], Qingzhu Gao[7], Guiji Zhang[7], Changlong He[7], Wei Wang[11], Xiaoyun Ji[5,11], Ni Tang[7], Zhenghong Yuan[3], Youhua Xie[3,#], Haitao Yang[4,#], Bo Zhang[8,#], Ailong Huang[7,#]& Aishun Jin[1,2, #]

[1]Department of Immunology, College of Basic Medicine, Chongqing Medical University, Chongqing, 400010, China

[2]Chongqing Key Laboratory of Basic and Translational Research of Tumor Immunology, Chongqing Medical University, Chongqing, 400010, China

[3]Key Laboratory of Medical Molecular Virology, Department of Medical Microbiology and Parasitology, School of Basic Medical Sciences, Shanghai Medical College, Fudan University, Shanghai, 200032, China

[4]Shanghai Institute for Advanced Immunochemical Studies and School of Life Science and Technology, ShanghaiTech University, Shanghai, 201210, China

[5]State Key Laboratory of Pharmaceutical Biotechnology, School of Life Sciences, Nanjing University, Nanjing, Jiangsu, 210023, China

[6]State Key Laboratory of Virology, Wuhan Institute of Virology, Center for Biosafety Mega-Science, Chinese Academy of Sciences, Wuhan, 430071, China

[7]Key Laboratory of Molecular Biology on Infectious Diseases, Ministry of Education, Chongqing Medical University, Chongqing, 400010, China

[8]Key Laboratory of Special Pathogens and Biosafety, Wuhan Institute of Virology, Center for Biosafety Mega-Science, Chinese Academy of Sciences, Wuhan, 430071, China

[9]University of Chinese Academy of Sciences, Beijing, 100049, China

[10]Department of Breast Surgery, Harbin Medical University Cancer Hospital, Harbin, 150000, China

[11]Institute of life sciences, Chongqing Medical University, Chongqing, 400010, China

[*]These authors contributed equally: Tingting Li, Xiaojian Han, Chenjian Gu, Hangtian Guo, Huajun Zhang, Yingming Wang.

[#]email: yhxie@fudan.edu.cn (Y.H.X); yanght@shanghaitech.edu.cn (H.T.Y); zhangbo@wh.iov.cn (B.Z); ahuang@cqmu.edu.cn (A.L.H); aishunjin@cqmu.edu.cn (A.S.J)

Supplementary information

Supplementary Figures. 1-12

Supplementary Tables 1-3

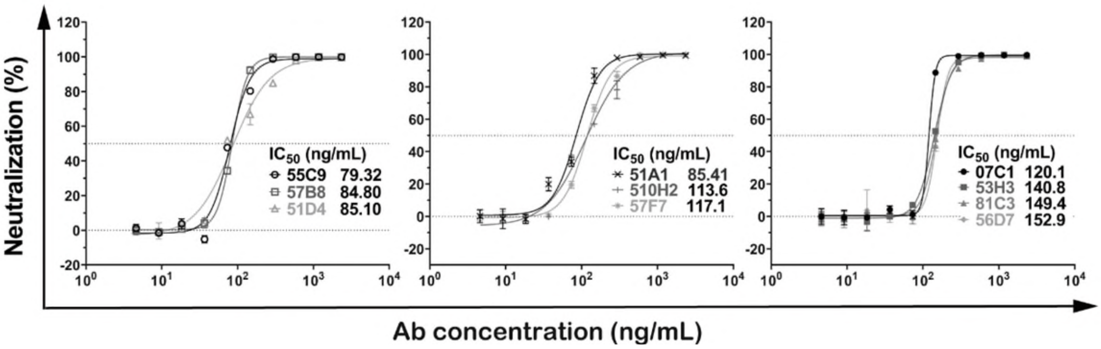

Supplementary Fig. 1 The neutralizing efficacy of top 11-20 mAbs against authentic SARS-CoV-2 virus. The capabilities of the top 11-20 mAbs were measured by authentic SARS-CoV-2 (nCoV-SH01) neutralization assay and quantified by qRT-PCR. Dashed line indicated 0% or 50% reduction in viral neutralization. Data for each mAb were obtained from a representative neutralization experiment of three replicates, presented as mean values ± SEM.

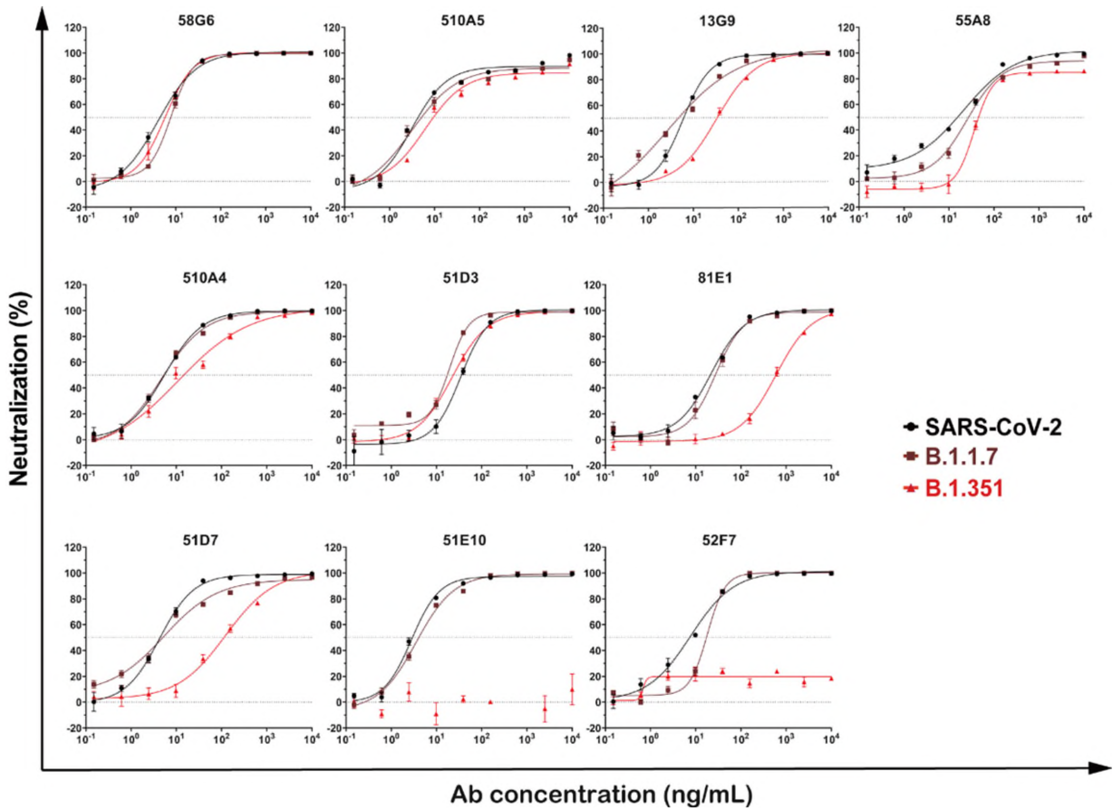

Supplementary Fig. 2 The neutralizing capabilities of selected mAbs against SARS-CoV-2, B.1.1.7 and B.1.351 pseudoviruses. The neutralizing potencies of the top 10 mAbs against SARS-CoV-2, B.1.1.7 and B.1.351 were measured by pseudovirus neutralization assay. Dashed line indicated 0% or 50% reduction in the viral neutralization. Data for each mAb were obtained from a representative neutralization experiment of three replicates, presented as mean values ± SEM.

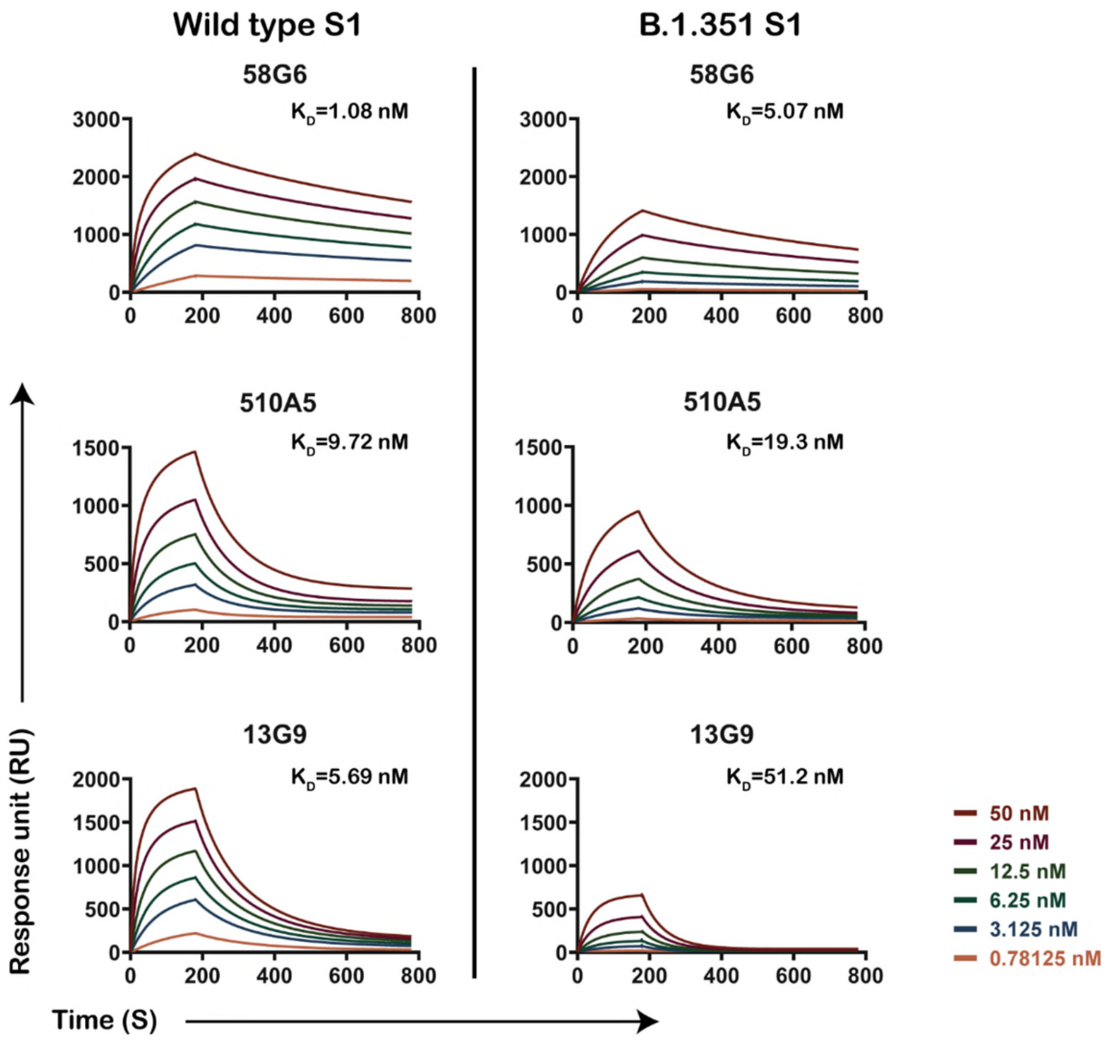

Supplementary Fig. 3 The binding kinetics of 58G6, 510A5 and 13G9 to SARS-CoV-2 S1 or B.1.351 S1. The binding kinetics was determined by the SPR assay. The purified mAbs were coated on the CM5 sensor chip followed by the injection of various concentrations of soluble recombinant SARS-CoV-2 S1 or B.1.351 S1 proteins. Data were representative of 2 independent experiments.

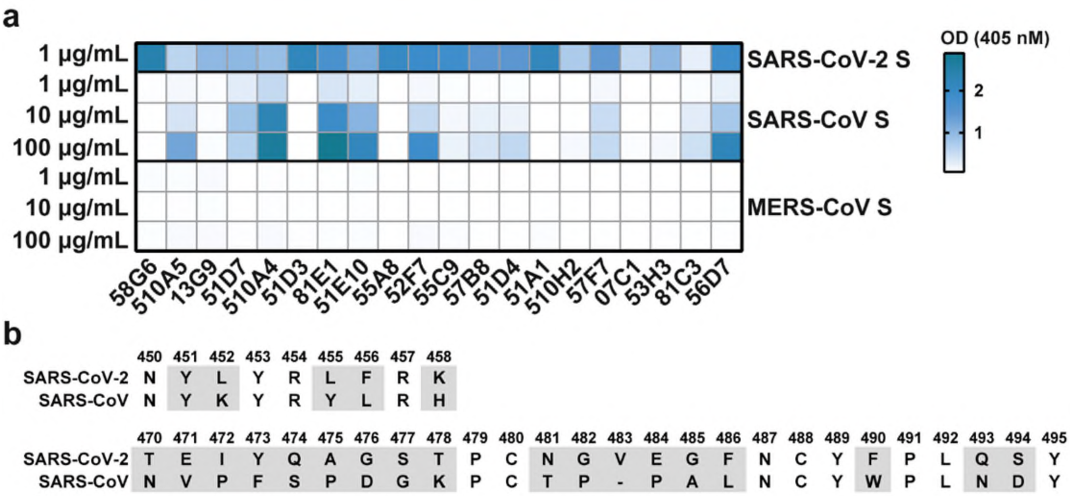

Supplementary Fig. 4 The binding characteristics of the top 20 mAbs to SARS-CoV-2 S, SARS-CoV S and MERS-CoV S. (**a**) The binding capability of these top 20 mAbs to the S protein of SARS-CoV-2, SARS-CoV or MERS-CoV was tested with various concentrations of the mAbs via ELISA. Data were representative of 2 independent experiments performed in technical duplicate. (**b**) Sequence alignment with the amino acids corresponding to the antigenic sites of 13G9 or 58G6 on the S protein of SARS-CoV-2 and SARS-CoV. The non-conserved residues between these two viruses were displayed in grey.

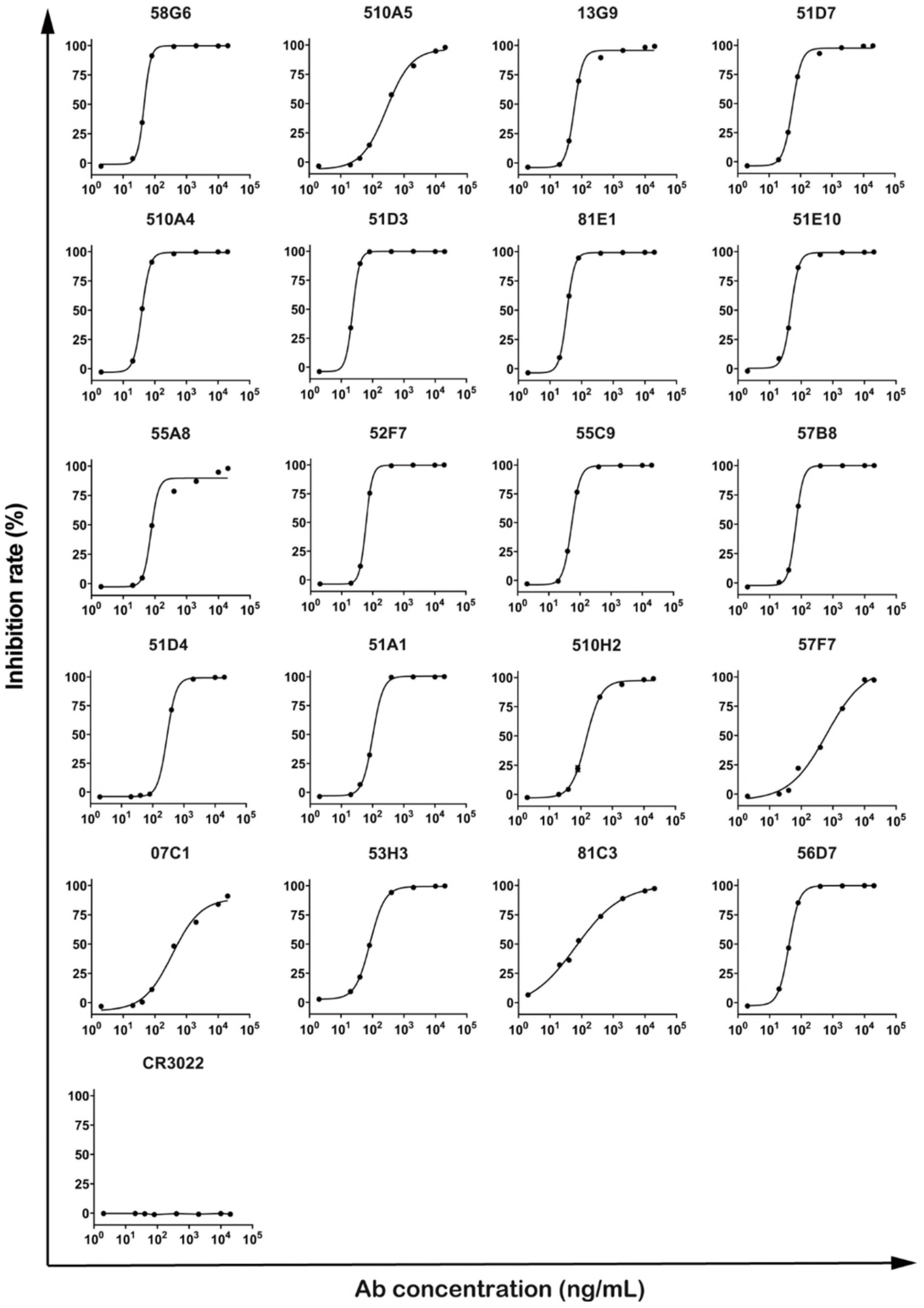

Supplementary Fig. 5 The inhibition curves for the neutralizing Abs on the ACE2-RBD interaction. Competitive ELISA for the neutralizing Abs to inhibit the ACE2-RBD interaction. The SARS-CoV specific mAb CR3022 was used as the negative control (n=2 biologically independent samples).

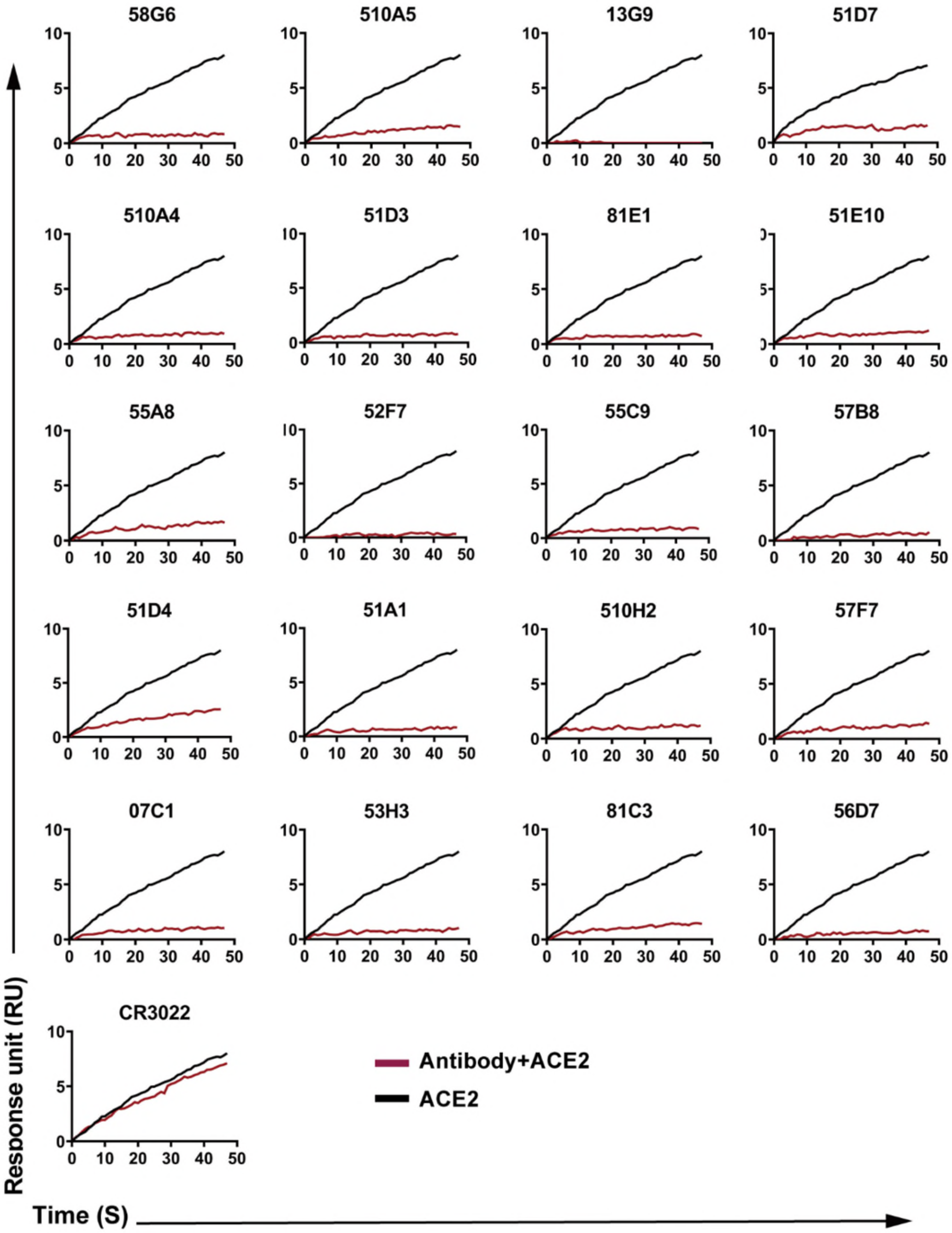

Supplementary Fig. 6 Neutralizing Abs competing with ACE2 for the binding of RBD. In SPR assay, the purified soluble SARS-CoV-2 RBD protein was coated onto a CM5 sensor chip followed by injection of individual neutralizing Abs at concentration of 20 μg/mL. The competition capacity of each antibody was indicated by the level of reduction in the response unit of ACE2 comparing with or without prior antibody incubation. The SARS-CoV RBD mAb CR3022 was used as the negative control.

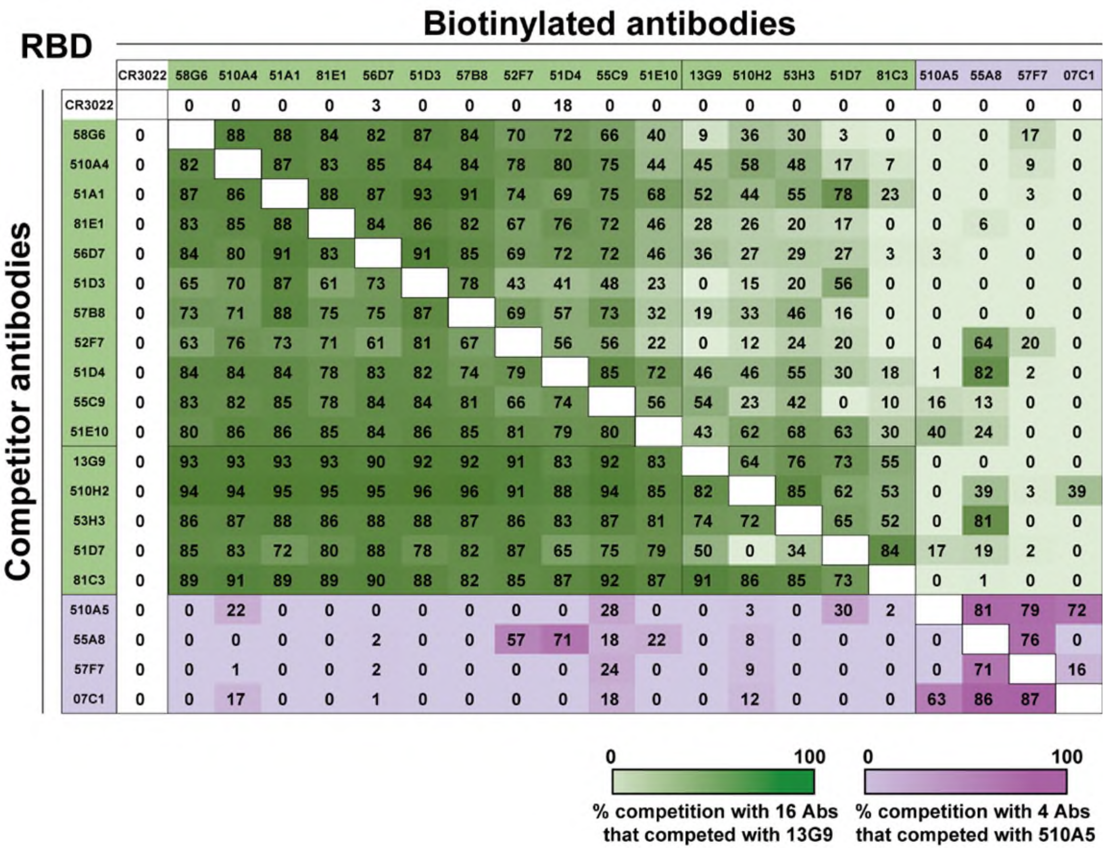

Supplementary Fig. 7 The competitive analysis of potential epitopes recognized by the top 20 neutralizing Abs. Each of our 20 mAb was biotinylated and competed with other unmodified mAbs for the identification of corresponding epitopes. The number in each box indicated the inhibition rate between two mAbs tested by competitive ELISA. Results were representatives of two independent experiments.

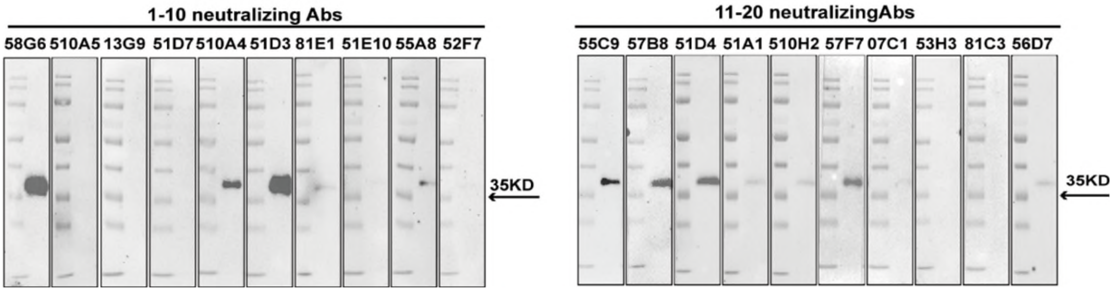

Supplementary Fig. 8 The binding ability of purified mAbs to the denatured RBD. Western blot results of the 1-10 (left) and 11-20 (right) neutralizing Abs binding to the denatured RBD. Data for each mAb were obtained from a representative of two dependent experiments.

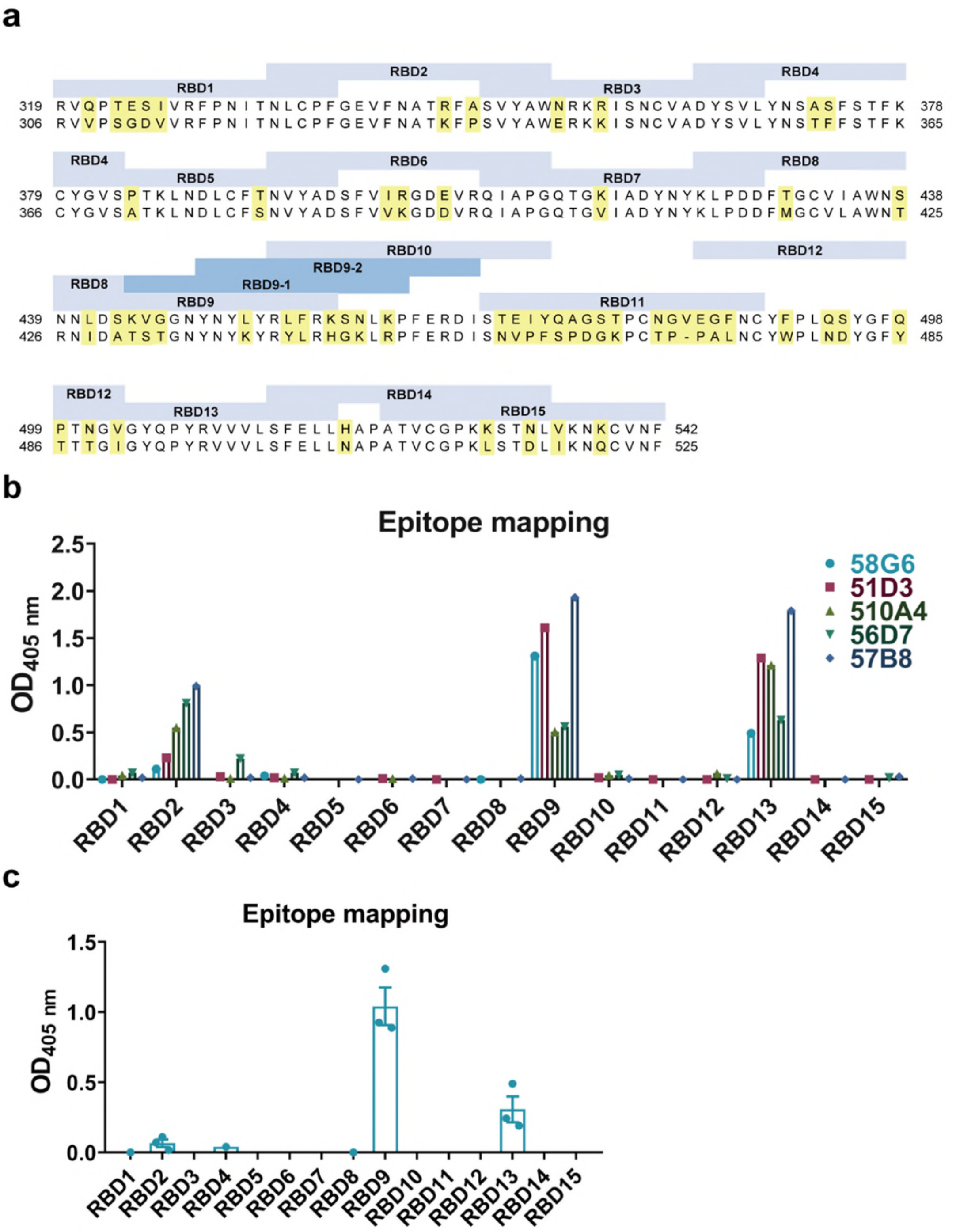

Supplementary Fig. 9 The binding ability of purified mAbs to the RBD related peptides. (**a**) The amino acid sequences for the RBDs of SARS-CoV-2 and SARS-CoV were aligned for comparison. Linear peptides designed for SARS-CoV-2 were shown in ash blue and the names of the peptides were indicated in the boxes above. The non-conserved residues between two viruses were displayed in yellow. (**b**) The interactions

of 5 out of 9 mAbs (58G6, 510A4, 51D3, 57B8 and 56D7) capable of binding to the denatured RBD to the linear peptides (RBD1-RBD15) were analyzed by peptide ELISA. The other 4 mAbs were uncapable to react with synthesized peptides. (c) The interactions of 58G6 to RBD1-RBD15 were analyzed by peptide ELISA (n=3 biologically independent samples). Data were presented as mean values ± SEM.

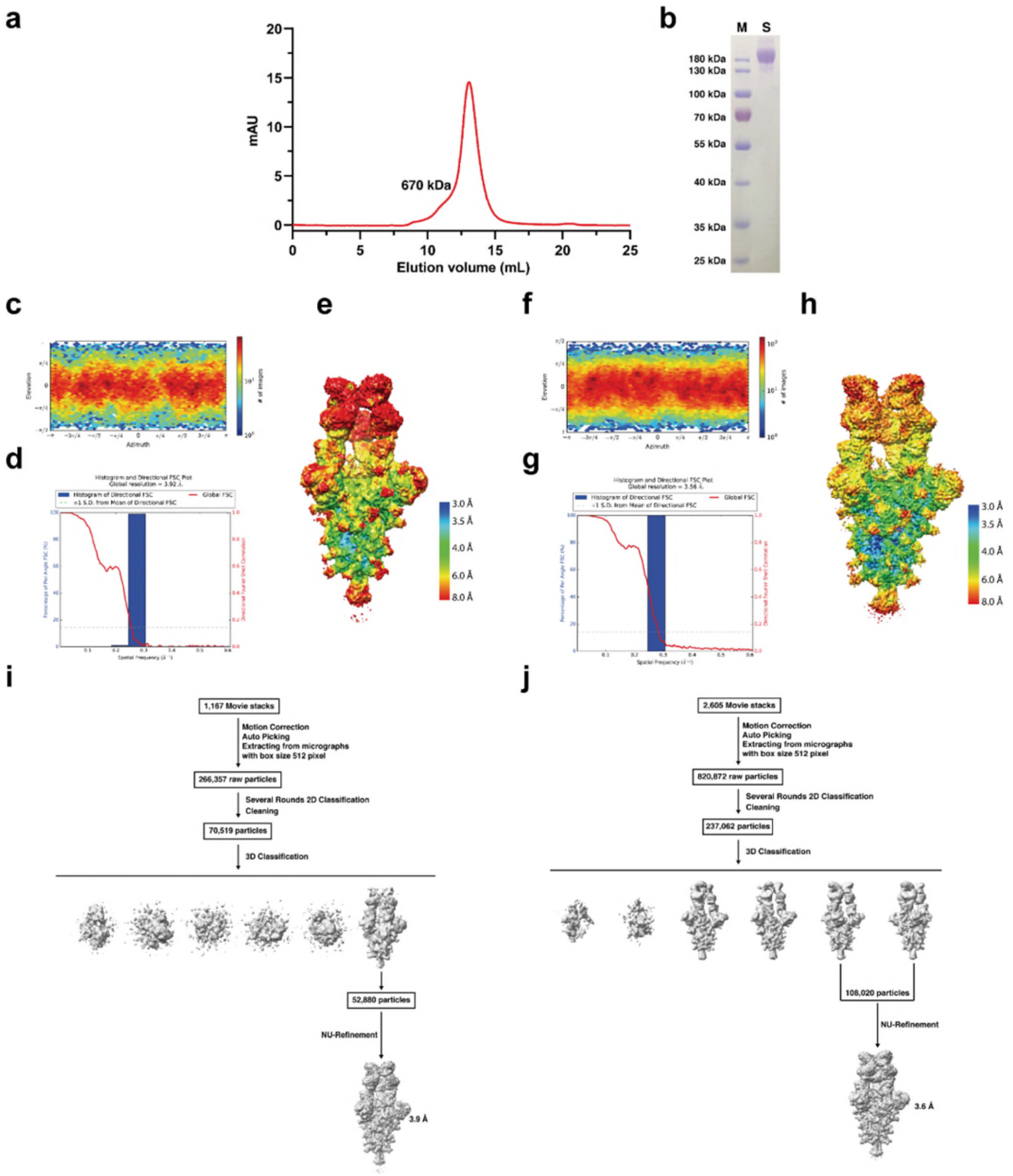

Supplementary Fig. 10 Workflow for 13G9 and 58G6 cryo-EM 3D Reconstructions. (**a**) Gel filtration profile of the affinity-purified SARS-CoV-2 S trimer. (**b**) SDS-PAGE analysis of the SARS-CoV-2 S protein. Data are representative of 2 independent experiments performed. (**c, g**) Cryo-EM data processing workflow of 13G9 (**c**) and 58G6 (**g**). (**d, h**) The viewing direction distribution plot for SARS-CoV-2 S in complex with 13G9 Fab (**d**) and 158G6 (**h**). (**e, i**) Global FSC and histogram. (**f, j**) Cryo-EM density of SARS-CoV-2 S-Fab complexes, colored according to local

resolution, including locally refined reconstruction of the RBD-13G9 (**f**) or RBD-58G6 (**j**) variable domains.

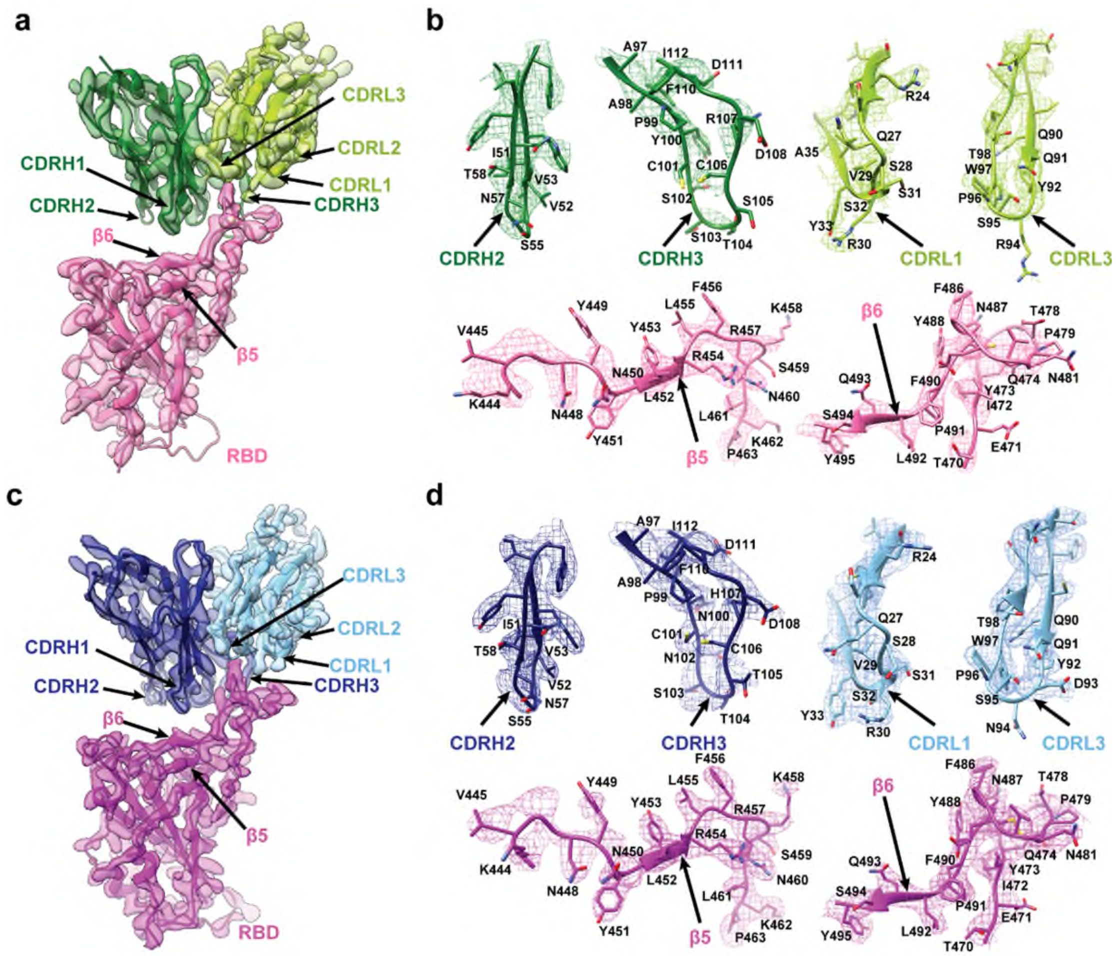

Supplementary Fig. 11 Density maps and atomic models. (**a, c**) Cryo-EM maps of the binding interface between SARS-CoV-2 RBD and 13G9 Fab (**a**) or 58G6 Fab (**c**) variable domains. The color scheme is the same as in Fig. 4 and 5. (**b, d**) Density maps (mesh) and related 13G9 CDRs (**b**) or 58G6 CDRs (**d**) atomic models. Residues are shown as sticks, oxygen atoms are colored red, nitrogen atoms are colored blue and sulfurs are shown in yellow.

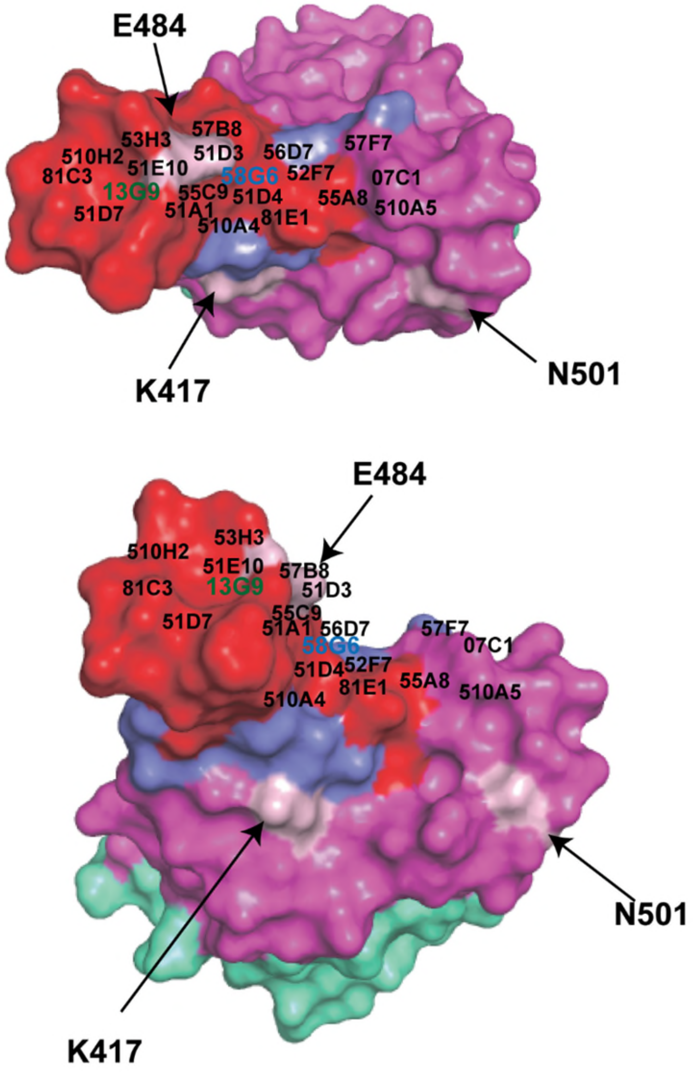

Supplementary Fig. 12 Mapping of the epitopes for the top 20 neutralizing Abs onto the surface of the viral RBD. The data for mapping calculations were from Supplementary Fig. 7. The view is chosen for clarity and is related to that shown in Fig. 5a, b. The epitopes for 58G6 and 13G9 were distinguished according to the color shown in Fig. 5a, b. The epitope for CR3022 was colored cyan. Positions of the S^{K417}, S^{E484}, and S^{N501} were labeled with pink.

Supplementary Table 1. Cryo-EM data collection and models refinement statistics

EM Data collection and reconstruction statistics

Protein	S-58G6 Fab	S-13G9 Fab
Voltage (kV)	300	300
Detector	K3	K3
Pixel size (Å)	0.82	0.82
Electron dose (e^-/ $Å^2$)	60	60
Defocus range	1.0-2.8	1.0-2.8
Micrographs collected	2605	1167
Particles initial/final	820,872/106,020	266,357/52,880
Final resolution (Å)	3.56	3.92

Models refinement and validation statistics

Protein	S-58G6 Fab	S-13G9 Fab
**RMSD**		
Bond lengths (Å)	0.007	0.008
Bond angles (°)	1.129	1.173
**Ramachandran statistics**		
Favored (%)	91.00	91.98
Allowed (%)	8.91	7.93
Outliers (%)	0.09	0.09
Rotamer outliers (%)	0.32	0.12
Clash score	8.99	12.66
C-β utliers (%)	0.10	0.03
CaBLAM outliers (%)	4.01	3.93

Supplementary Table 2. The information of 58G6 and 13G9 variable genes and sequences.

mAbs	VDJ information				CDR information					
	VH	JH	VL	JL	CDRH1	CDRL1	CDRH2	CDRL2	CDRH3	CDRL3
58G6	IGHV1-58	IGHJ3-2	IGKV3-20	IGKJ1-1	GFTFSSSA	QSVRSSY	IVVGSGNT	GAS	AAPNCNSTTCHDGFDI	QQYDN*SPWT
13G9	IGHV1-58	IGHJ3-2	IGKV3-20	IGKJ1-1	GFTFSGSA	QSVRSSY	IVVGSGNT	GAS	AAPYCSSTSCRDGFDI	QQYGR*SPWT

VDJ alignment was determined by IgBLAST. The CDR sequences of 58G6 and 13G9 are aligned. Distinct amino acids were marked in red. The 94th amino acid on the CDRH3 of mAb interacting with S^{E484} is labelled with asterisk.

Supplementary Table 3. The list of primers.

Gene name	Forward primer	Reverse primer
SARS-CoV-2 N gene (nt 608-706)	5’-GGGGAACTTCTCCTGCTAGAAT-3’	5’-CAGACATTTTGCTCTCAAGCTG-3’
