# Supplementary material for: Potent SARS-CoV-2 neutralizing antibodies with protective efficacy against newly emerged mutational variants
Source: Nat Commun. 2021 Nov 2;12:6304. doi: 10.1038/s41467-021-26539-7 (PMC8563728; doi:10.1038/s41467-021-26539-7)
Supplement: Supplementary file 5 — Full wwPDB EM Validation Report for 7E3K [file 41467_2021_26539_MOESM5_ESM.pdf]

Full wwPDB EM Validation Report (i)

Apr 22, 2021 – 03:46 PM JST

PDB ID	:	7E3K
EMDB ID	:	EMD-30982
Title	:	Ultrapotent SARS-CoV-2 neutralizing antibodies with protective efficacy against newly emerged mutational variants
Deposited on	:	2021-02-09
Resolution	:	3.90 Å(reported)

This is a Full wwPDB EM Validation Report.

This report is produced by the wwPDB biocuration pipeline after annotation of the structure.

We welcome your comments at *validation@mail.wwpdb.org*
A user guide is available at
https://www.wwpdb.org/validation/2017/EMValidationReportHelp
with specific help available everywhere you see the (i) symbol.

CONFIDENTIAL VALIDATION REPORT

The following versions of software and data (see references (i)) were used in the production of this report:

EMDB validation analysis	:	0.0.0.dev75
Mogul	:	1.8.5 (274361), CSD as541be (2020)
MolProbity	:	4.02b-467
buster-report	:	1.1.7 (2018)
Percentile statistics	:	20191225.v01 (using entries in the PDB archive December 25th 2019)
Ideal geometry (proteins)	:	Engh & Huber (2001)
Ideal geometry (DNA, RNA)	:	Parkinson et al. (1996)
Validation Pipeline (wwPDB-VP)	:	2.18

1 Overall quality at a glance ⓘ

The following experimental techniques were used to determine the structure:
ELECTRON MICROSCOPY

The reported resolution of this entry is 3.90 Å.

Percentile scores (ranging between 0-100) for global validation metrics of the entry are shown in the following graphic. The table shows the number of entries on which the scores are based.

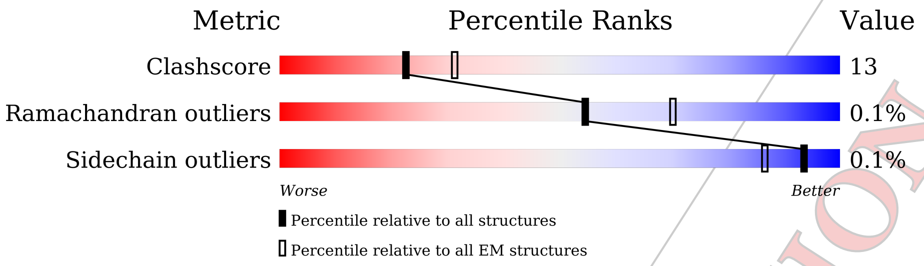

Metric	Whole archive (#Entries)	EM structures (#Entries)
Clashscore	158937	4297
Ramachandran outliers	154571	4023
Sidechain outliers	154315	3826

The table below summarises the geometric issues observed across the polymeric chains and their fit to the map. The red, orange, yellow and green segments of the bar indicate the fraction of residues that contain outliers for >=3, 2, 1 and 0 types of geometric quality criteria respectively. A grey segment represents the fraction of residues that are not modelled. The numeric value for each fraction is indicated below the corresponding segment, with a dot representing fractions <=5% The upper red bar (where present) indicates the fraction of residues that have poor fit to the EM map (all-atom inclusion < 40%). The numeric value is given above the bar.

Mol	Chain	Length	Quality of chain
1	A	1288	61% 16% 23%
1	B	1288	60% 16% 23%
1	C	1288	62% 15% 23%
2	D	221	7% / 72% 28%
2	F	221	5% / 72% 28%
2	H	221	15% / 71% 28%
3	E	215	6% / 67% 33%
3	G	215	5% / 68% 32%

Continued on next page...

CONFIDENTIAL VALIDATION REPORT

Continued from previous page...

Mol	Chain	Length	Quality of chain
3	L	215	8% / 67% / 33%

CONFIDENTIAL VALIDATION REPORT

2 Entry composition (i)

There are 4 unique types of molecules in this entry. The entry contains 32539 atoms, of which 0 are hydrogens and 0 are deuteriums.

In the tables below, the AltConf column contains the number of residues with at least one atom in alternate conformation and the Trace column contains the number of residues modelled with at most 2 atoms.

- Molecule 1 is a protein called Spike glycoprotein.

Mol	Chain	Residues	Atoms					AltConf	Trace
1	A	990	Total 7591	C 4859	N 1261	O 1441	S 30	0	0
1	B	990	Total 7571	C 4846	N 1257	O 1439	S 29	0	0
1	C	990	Total 7577	C 4847	N 1262	O 1438	S 30	0	0

There are 255 discrepancies between the modelled and reference sequences:

Chain	Residue	Modelled	Actual	Comment	Reference
A	682	GLY	ARG	engineered mutation	UNP P0DTC2
A	683	SER	ARG	engineered mutation	UNP P0DTC2
A	685	SER	ARG	engineered mutation	UNP P0DTC2
A	986	PRO	LYS	engineered mutation	UNP P0DTC2
A	987	PRO	VAL	engineered mutation	UNP P0DTC2
A	1209	GLY	-	expression tag	UNP P0DTC2
A	1210	SER	-	expression tag	UNP P0DTC2
A	1211	GLY	-	expression tag	UNP P0DTC2
A	1212	TYR	-	expression tag	UNP P0DTC2
A	1213	ILE	-	expression tag	UNP P0DTC2
A	1214	PRO	-	expression tag	UNP P0DTC2
A	1215	GLU	-	expression tag	UNP P0DTC2
A	1216	ALA	-	expression tag	UNP P0DTC2
A	1217	PRO	-	expression tag	UNP P0DTC2
A	1218	ARG	-	expression tag	UNP P0DTC2
A	1219	ASP	-	expression tag	UNP P0DTC2
A	1220	GLY	-	expression tag	UNP P0DTC2
A	1221	GLN	-	expression tag	UNP P0DTC2
A	1222	ALA	-	expression tag	UNP P0DTC2
A	1223	TYR	-	expression tag	UNP P0DTC2
A	1224	VAL	-	expression tag	UNP P0DTC2
A	1225	ARG	-	expression tag	UNP P0DTC2
A	1226	LYS	-	expression tag	UNP P0DTC2
A	1227	ASP	-	expression tag	UNP P0DTC2

Continued on next page...

CONFIDENTIAL VALIDATION REPORT

Continued from previous page...

Chain	Residue	Modelled	Actual	Comment	Reference
A	1228	GLY	-	expression tag	UNP P0DTC2
A	1229	GLU	-	expression tag	UNP P0DTC2
A	1230	TRP	-	expression tag	UNP P0DTC2
A	1231	VAL	-	expression tag	UNP P0DTC2
A	1232	PHE	-	expression tag	UNP P0DTC2
A	1233	LEU	-	expression tag	UNP P0DTC2
A	1234	SER	-	expression tag	UNP P0DTC2
A	1235	THR	-	expression tag	UNP P0DTC2
A	1236	PHE	-	expression tag	UNP P0DTC2
A	1237	LEU	-	expression tag	UNP P0DTC2
A	1238	SER	-	expression tag	UNP P0DTC2
A	1239	GLY	-	expression tag	UNP P0DTC2
A	1240	LEU	-	expression tag	UNP P0DTC2
A	1241	GLU	-	expression tag	UNP P0DTC2
A	1242	VAL	-	expression tag	UNP P0DTC2
A	1243	LEU	-	expression tag	UNP P0DTC2
A	1244	PHE	-	expression tag	UNP P0DTC2
A	1245	GLN	-	expression tag	UNP P0DTC2
A	1246	GLY	-	expression tag	UNP P0DTC2
A	1247	PRO	-	expression tag	UNP P0DTC2
A	1248	GLY	-	expression tag	UNP P0DTC2
A	1249	GLY	-	expression tag	UNP P0DTC2
A	1250	TRP	-	expression tag	UNP P0DTC2
A	1251	SER	-	expression tag	UNP P0DTC2
A	1252	HIS	-	expression tag	UNP P0DTC2
A	1253	PRO	-	expression tag	UNP P0DTC2
A	1254	GLN	-	expression tag	UNP P0DTC2
A	1255	PHE	-	expression tag	UNP P0DTC2
A	1256	GLU	-	expression tag	UNP P0DTC2
A	1257	LYS	-	expression tag	UNP P0DTC2
A	1258	GLY	-	expression tag	UNP P0DTC2
A	1259	GLY	-	expression tag	UNP P0DTC2
A	1260	GLY	-	expression tag	UNP P0DTC2
A	1261	SER	-	expression tag	UNP P0DTC2
A	1262	GLY	-	expression tag	UNP P0DTC2
A	1263	GLY	-	expression tag	UNP P0DTC2
A	1264	GLY	-	expression tag	UNP P0DTC2
A	1265	SER	-	expression tag	UNP P0DTC2
A	1266	GLY	-	expression tag	UNP P0DTC2
A	1267	GLY	-	expression tag	UNP P0DTC2
A	1268	SER	-	expression tag	UNP P0DTC2
A	1269	ALA	-	expression tag	UNP P0DTC2

Continued on next page...

CONFIDENTIAL VALIDATION REPORT

Continued from previous page...

Chain	Residue	Modelled	Actual	Comment	Reference
A	1270	TRP	-	expression tag	UNP P0DTC2
A	1271	SER	-	expression tag	UNP P0DTC2
A	1272	HIS	-	expression tag	UNP P0DTC2
A	1273	PRO	-	expression tag	UNP P0DTC2
A	1274	GLN	-	expression tag	UNP P0DTC2
A	1275	PHE	-	expression tag	UNP P0DTC2
A	1276	GLU	-	expression tag	UNP P0DTC2
A	1277	LYS	-	expression tag	UNP P0DTC2
A	1278	GLY	-	expression tag	UNP P0DTC2
A	1279	GLY	-	expression tag	UNP P0DTC2
A	1280	SER	-	expression tag	UNP P0DTC2
A	1281	HIS	-	expression tag	UNP P0DTC2
A	1282	HIS	-	expression tag	UNP P0DTC2
A	1283	HIS	-	expression tag	UNP P0DTC2
A	1284	HIS	-	expression tag	UNP P0DTC2
A	1285	HIS	-	expression tag	UNP P0DTC2
A	1286	HIS	-	expression tag	UNP P0DTC2
A	1287	HIS	-	expression tag	UNP P0DTC2
A	1288	HIS	-	expression tag	UNP P0DTC2
B	682	GLY	ARG	engineered mutation	UNP P0DTC2
B	683	SER	ARG	engineered mutation	UNP P0DTC2
B	685	SER	ARG	engineered mutation	UNP P0DTC2
B	986	PRO	LYS	engineered mutation	UNP P0DTC2
B	987	PRO	VAL	engineered mutation	UNP P0DTC2
B	1209	GLY	-	expression tag	UNP P0DTC2
B	1210	SER	-	expression tag	UNP P0DTC2
B	1211	GLY	-	expression tag	UNP P0DTC2
B	1212	TYR	-	expression tag	UNP P0DTC2
B	1213	ILE	-	expression tag	UNP P0DTC2
B	1214	PRO	-	expression tag	UNP P0DTC2
B	1215	GLU	-	expression tag	UNP P0DTC2
B	1216	ALA	-	expression tag	UNP P0DTC2
B	1217	PRO	-	expression tag	UNP P0DTC2
B	1218	ARG	-	expression tag	UNP P0DTC2
B	1219	ASP	-	expression tag	UNP P0DTC2
B	1220	GLY	-	expression tag	UNP P0DTC2
B	1221	GLN	-	expression tag	UNP P0DTC2
B	1222	ALA	-	expression tag	UNP P0DTC2
B	1223	TYR	-	expression tag	UNP P0DTC2
B	1224	VAL	-	expression tag	UNP P0DTC2
B	1225	ARG	-	expression tag	UNP P0DTC2
B	1226	LYS	-	expression tag	UNP P0DTC2

Continued on next page...

CONFIDENTIAL VALIDATION REPORT

Continued from previous page...

Chain	Residue	Modelled	Actual	Comment	Reference
B	1227	ASP	-	expression tag	UNP P0DTC2
B	1228	GLY	-	expression tag	UNP P0DTC2
B	1229	GLU	-	expression tag	UNP P0DTC2
B	1230	TRP	-	expression tag	UNP P0DTC2
B	1231	VAL	-	expression tag	UNP P0DTC2
B	1232	PHE	-	expression tag	UNP P0DTC2
B	1233	LEU	-	expression tag	UNP P0DTC2
B	1234	SER	-	expression tag	UNP P0DTC2
B	1235	THR	-	expression tag	UNP P0DTC2
B	1236	PHE	-	expression tag	UNP P0DTC2
B	1237	LEU	-	expression tag	UNP P0DTC2
B	1238	SER	-	expression tag	UNP P0DTC2
B	1239	GLY	-	expression tag	UNP P0DTC2
B	1240	LEU	-	expression tag	UNP P0DTC2
B	1241	GLU	-	expression tag	UNP P0DTC2
B	1242	VAL	-	expression tag	UNP P0DTC2
B	1243	LEU	-	expression tag	UNP P0DTC2
B	1244	PHE	-	expression tag	UNP P0DTC2
B	1245	GLN	-	expression tag	UNP P0DTC2
B	1246	GLY	-	expression tag	UNP P0DTC2
B	1247	PRO	-	expression tag	UNP P0DTC2
B	1248	GLY	-	expression tag	UNP P0DTC2
B	1249	GLY	-	expression tag	UNP P0DTC2
B	1250	TRP	-	expression tag	UNP P0DTC2
B	1251	SER	-	expression tag	UNP P0DTC2
B	1252	HIS	-	expression tag	UNP P0DTC2
B	1253	PRO	-	expression tag	UNP P0DTC2
B	1254	GLN	-	expression tag	UNP P0DTC2
B	1255	PHE	-	expression tag	UNP P0DTC2
B	1256	GLU	-	expression tag	UNP P0DTC2
B	1257	LYS	-	expression tag	UNP P0DTC2
B	1258	GLY	-	expression tag	UNP P0DTC2
B	1259	GLY	-	expression tag	UNP P0DTC2
B	1260	GLY	-	expression tag	UNP P0DTC2
B	1261	SER	-	expression tag	UNP P0DTC2
B	1262	GLY	-	expression tag	UNP P0DTC2
B	1263	GLY	-	expression tag	UNP P0DTC2
B	1264	GLY	-	expression tag	UNP P0DTC2
B	1265	SER	-	expression tag	UNP P0DTC2
B	1266	GLY	-	expression tag	UNP P0DTC2
B	1267	GLY	-	expression tag	UNP P0DTC2
B	1268	SER	-	expression tag	UNP P0DTC2

Continued on next page...

CONFIDENTIAL VALIDATION REPORT

CONFIDENTIAL VALIDATION REPORT

Continued from previous page...

Chain	Residue	Modelled	Actual	Comment	Reference
B	1269	ALA	-	expression tag	UNP P0DTC2
B	1270	TRP	-	expression tag	UNP P0DTC2
B	1271	SER	-	expression tag	UNP P0DTC2
B	1272	HIS	-	expression tag	UNP P0DTC2
B	1273	PRO	-	expression tag	UNP P0DTC2
B	1274	GLN	-	expression tag	UNP P0DTC2
B	1275	PHE	-	expression tag	UNP P0DTC2
B	1276	GLU	-	expression tag	UNP P0DTC2
B	1277	LYS	-	expression tag	UNP P0DTC2
B	1278	GLY	-	expression tag	UNP P0DTC2
B	1279	GLY	-	expression tag	UNP P0DTC2
B	1280	SER	-	expression tag	UNP P0DTC2
B	1281	HIS	-	expression tag	UNP P0DTC2
B	1282	HIS	-	expression tag	UNP P0DTC2
B	1283	HIS	-	expression tag	UNP P0DTC2
B	1284	HIS	-	expression tag	UNP P0DTC2
B	1285	HIS	-	expression tag	UNP P0DTC2
B	1286	HIS	-	expression tag	UNP P0DTC2
B	1287	HIS	-	expression tag	UNP P0DTC2
B	1288	HIS	-	expression tag	UNP P0DTC2
C	682	GLY	ARG	engineered mutation	UNP P0DTC2
C	683	SER	ARG	engineered mutation	UNP P0DTC2
C	685	SER	ARG	engineered mutation	UNP P0DTC2
C	986	PRO	LYS	engineered mutation	UNP P0DTC2
C	987	PRO	VAL	engineered mutation	UNP P0DTC2
C	1209	GLY	-	expression tag	UNP P0DTC2
C	1210	SER	-	expression tag	UNP P0DTC2
C	1211	GLY	-	expression tag	UNP P0DTC2
C	1212	TYR	-	expression tag	UNP P0DTC2
C	1213	ILE	-	expression tag	UNP P0DTC2
C	1214	PRO	-	expression tag	UNP P0DTC2
C	1215	GLU	-	expression tag	UNP P0DTC2
C	1216	ALA	-	expression tag	UNP P0DTC2
C	1217	PRO	-	expression tag	UNP P0DTC2
C	1218	ARG	-	expression tag	UNP P0DTC2
C	1219	ASP	-	expression tag	UNP P0DTC2
C	1220	GLY	-	expression tag	UNP P0DTC2
C	1221	GLN	-	expression tag	UNP P0DTC2
C	1222	ALA	-	expression tag	UNP P0DTC2
C	1223	TYR	-	expression tag	UNP P0DTC2
C	1224	VAL	-	expression tag	UNP P0DTC2
C	1225	ARG	-	expression tag	UNP P0DTC2

Continued on next page...

Continued from previous page...

Chain	Residue	Modelled	Actual	Comment	Reference
C	1226	LYS	-	expression tag	UNP P0DTC2
C	1227	ASP	-	expression tag	UNP P0DTC2
C	1228	GLY	-	expression tag	UNP P0DTC2
C	1229	GLU	-	expression tag	UNP P0DTC2
C	1230	TRP	-	expression tag	UNP P0DTC2
C	1231	VAL	-	expression tag	UNP P0DTC2
C	1232	PHE	-	expression tag	UNP P0DTC2
C	1233	LEU	-	expression tag	UNP P0DTC2
C	1234	SER	-	expression tag	UNP P0DTC2
C	1235	THR	-	expression tag	UNP P0DTC2
C	1236	PHE	-	expression tag	UNP P0DTC2
C	1237	LEU	-	expression tag	UNP P0DTC2
C	1238	SER	-	expression tag	UNP P0DTC2
C	1239	GLY	-	expression tag	UNP P0DTC2
C	1240	LEU	-	expression tag	UNP P0DTC2
C	1241	GLU	-	expression tag	UNP P0DTC2
C	1242	VAL	-	expression tag	UNP P0DTC2
C	1243	LEU	-	expression tag	UNP P0DTC2
C	1244	PHE	-	expression tag	UNP P0DTC2
C	1245	GLN	-	expression tag	UNP P0DTC2
C	1246	GLY	-	expression tag	UNP P0DTC2
C	1247	PRO	-	expression tag	UNP P0DTC2
C	1248	GLY	-	expression tag	UNP P0DTC2
C	1249	GLY	-	expression tag	UNP P0DTC2
C	1250	TRP	-	expression tag	UNP P0DTC2
C	1251	SER	-	expression tag	UNP P0DTC2
C	1252	HIS	-	expression tag	UNP P0DTC2
C	1253	PRO	-	expression tag	UNP P0DTC2
C	1254	GLN	-	expression tag	UNP P0DTC2
C	1255	PHE	-	expression tag	UNP P0DTC2
C	1256	GLU	-	expression tag	UNP P0DTC2
C	1257	LYS	-	expression tag	UNP P0DTC2
C	1258	GLY	-	expression tag	UNP P0DTC2
C	1259	GLY	-	expression tag	UNP P0DTC2
C	1260	GLY	-	expression tag	UNP P0DTC2
C	1261	SER	-	expression tag	UNP P0DTC2
C	1262	GLY	-	expression tag	UNP P0DTC2
C	1263	GLY	-	expression tag	UNP P0DTC2
C	1264	GLY	-	expression tag	UNP P0DTC2
C	1265	SER	-	expression tag	UNP P0DTC2
C	1266	GLY	-	expression tag	UNP P0DTC2
C	1267	GLY	-	expression tag	UNP P0DTC2

Continued on next page...

CONFIDENTIAL VALIDATION REPORT

Continued from previous page...

Chain	Residue	Modelled	Actual	Comment	Reference
C	1268	SER	-	expression tag	UNP P0DTC2
C	1269	ALA	-	expression tag	UNP P0DTC2
C	1270	TRP	-	expression tag	UNP P0DTC2
C	1271	SER	-	expression tag	UNP P0DTC2
C	1272	HIS	-	expression tag	UNP P0DTC2
C	1273	PRO	-	expression tag	UNP P0DTC2
C	1274	GLN	-	expression tag	UNP P0DTC2
C	1275	PHE	-	expression tag	UNP P0DTC2
C	1276	GLU	-	expression tag	UNP P0DTC2
C	1277	LYS	-	expression tag	UNP P0DTC2
C	1278	GLY	-	expression tag	UNP P0DTC2
C	1279	GLY	-	expression tag	UNP P0DTC2
C	1280	SER	-	expression tag	UNP P0DTC2
C	1281	HIS	-	expression tag	UNP P0DTC2
C	1282	HIS	-	expression tag	UNP P0DTC2
C	1283	HIS	-	expression tag	UNP P0DTC2
C	1284	HIS	-	expression tag	UNP P0DTC2
C	1285	HIS	-	expression tag	UNP P0DTC2
C	1286	HIS	-	expression tag	UNP P0DTC2
C	1287	HIS	-	expression tag	UNP P0DTC2
C	1288	HIS	-	expression tag	UNP P0DTC2

- Molecule 2 is a protein called 13G9 heavy chain.

Mol	Chain	Residues	Atoms					AltConf	Trace
			Total	C	N	O	S		
2	D	221	1570	976	274	310	10	0	0
2	F	221	1570	976	274	310	10	0	0
2	H	221	1570	976	274	310	10	0	0

- Molecule 3 is a protein called 13G9 light chain.

Mol	Chain	Residues	Atoms					AltConf	Trace
			Total	C	N	O	S		
3	E	215	1594	991	277	321	5	0	0
3	G	215	1594	991	277	321	5	0	0
3	L	215	1594	991	277	321	5	0	0

CONFIDENTIAL VALIDATION REPORT

- Molecule 4 is 2-acetamido-2-deoxy-beta-D-glucopyranose (three-letter code: NAG) (formula: $C_8H_{15}NO_6$) (labeled as "Ligand of Interest" by depositor).

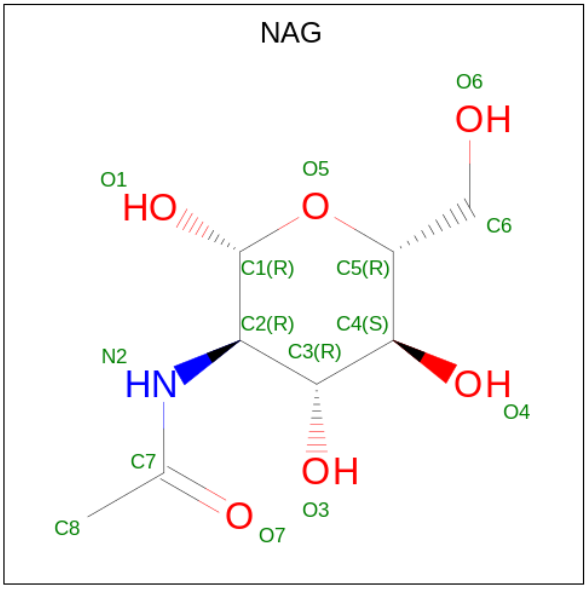

Mol	Chain	Residues	Atoms	AltConf
4	A	1	Total C N O<br>112 64 8 40	0
4	A	1	Total C N O<br>112 64 8 40	0
4	A	1	Total C N O<br>112 64 8 40	0
4	A	1	Total C N O<br>112 64 8 40	0
4	A	1	Total C N O<br>112 64 8 40	0
4	A	1	Total C N O<br>112 64 8 40	0
4	A	1	Total C N O<br>112 64 8 40	0
4	A	1	Total C N O<br>112 64 8 40	0
4	B	1	Total C N O<br>84 48 6 30	0
4	B	1	Total C N O<br>84 48 6 30	0
4	B	1	Total C N O<br>84 48 6 30	0
4	B	1	Total C N O<br>84 48 6 30	0

Continued on next page...

CONFIDENTIAL VALIDATION REPORT

Continued from previous page...

Mol	Chain	Residues	Atoms				AltConf
4	B	1	Total 84	C 48	N 6	O 30	0
4	B	1	Total 84	C 48	N 6	O 30	0
4	C	1	Total 112	C 64	N 8	O 40	0
4	C	1	Total 112	C 64	N 8	O 40	0
4	C	1	Total 112	C 64	N 8	O 40	0
4	C	1	Total 112	C 64	N 8	O 40	0
4	C	1	Total 112	C 64	N 8	O 40	0
4	C	1	Total 112	C 64	N 8	O 40	0
4	C	1	Total 112	C 64	N 8	O 40	0
4	C	1	Total 112	C 64	N 8	O 40	0

CONFIDENTIAL VALIDATION REPORT

3 Residue-property plots (i)

These plots are drawn for all protein, RNA, DNA and oligosaccharide chains in the entry. The first graphic for a chain summarises the proportions of the various outlier classes displayed in the second graphic. The second graphic shows the sequence view annotated by issues in geometry and atom inclusion in map density. Residues are color-coded according to the number of geometric quality criteria for which they contain at least one outlier: green = 0, yellow = 1, orange = 2 and red = 3 or more. A red diamond above a residue indicates a poor fit to the EM map for this residue (all-atom inclusion < 40%). Stretches of 2 or more consecutive residues without any outlier are shown as a green connector. Residues present in the sample, but not in the model, are shown in grey.

- Molecule 1: Spike glycoprotein

Chain A:

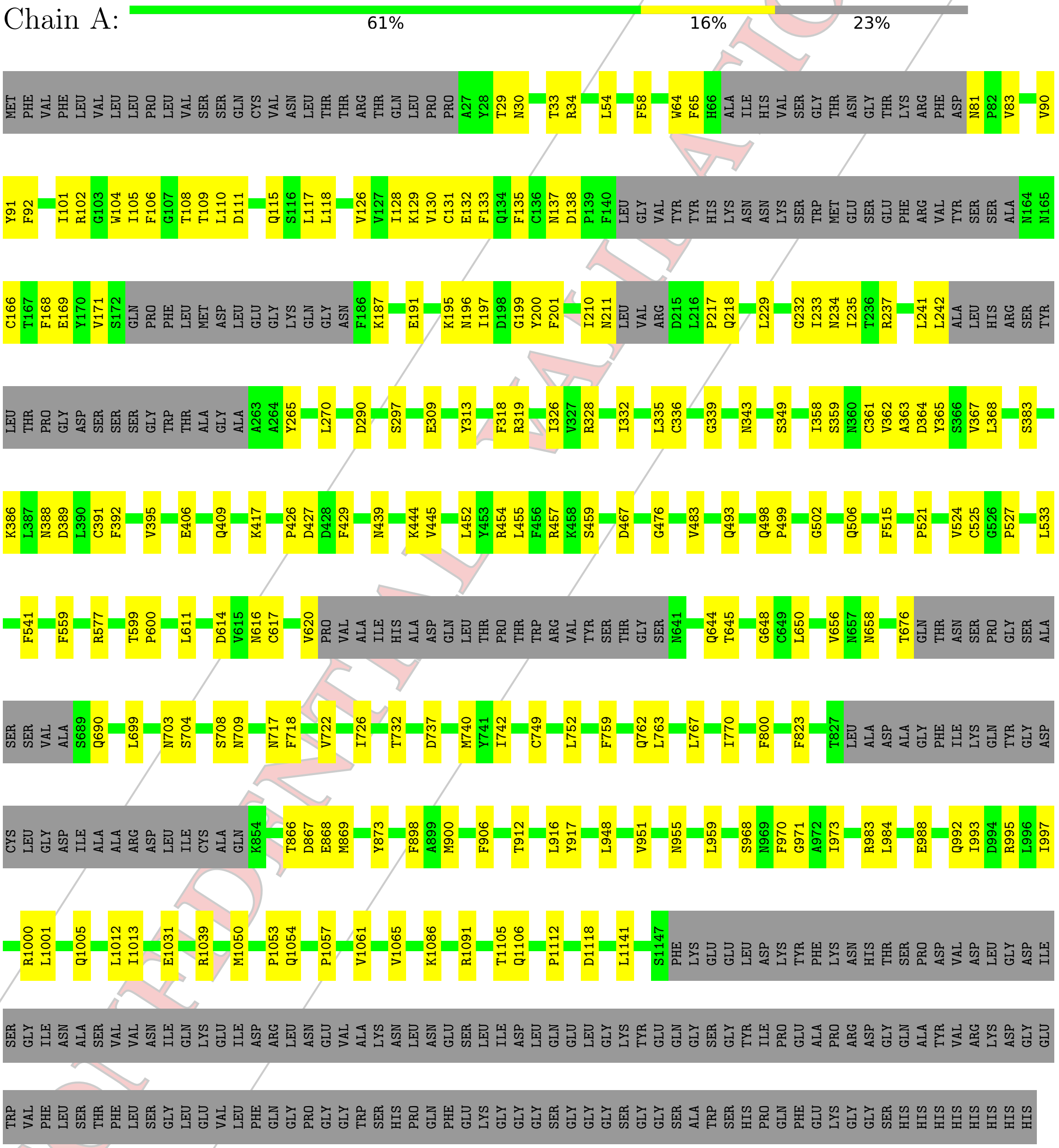

CONFIDENTIAL VALIDATION REPORT

- Molecule 1: Spike glycoprotein

Chain B:

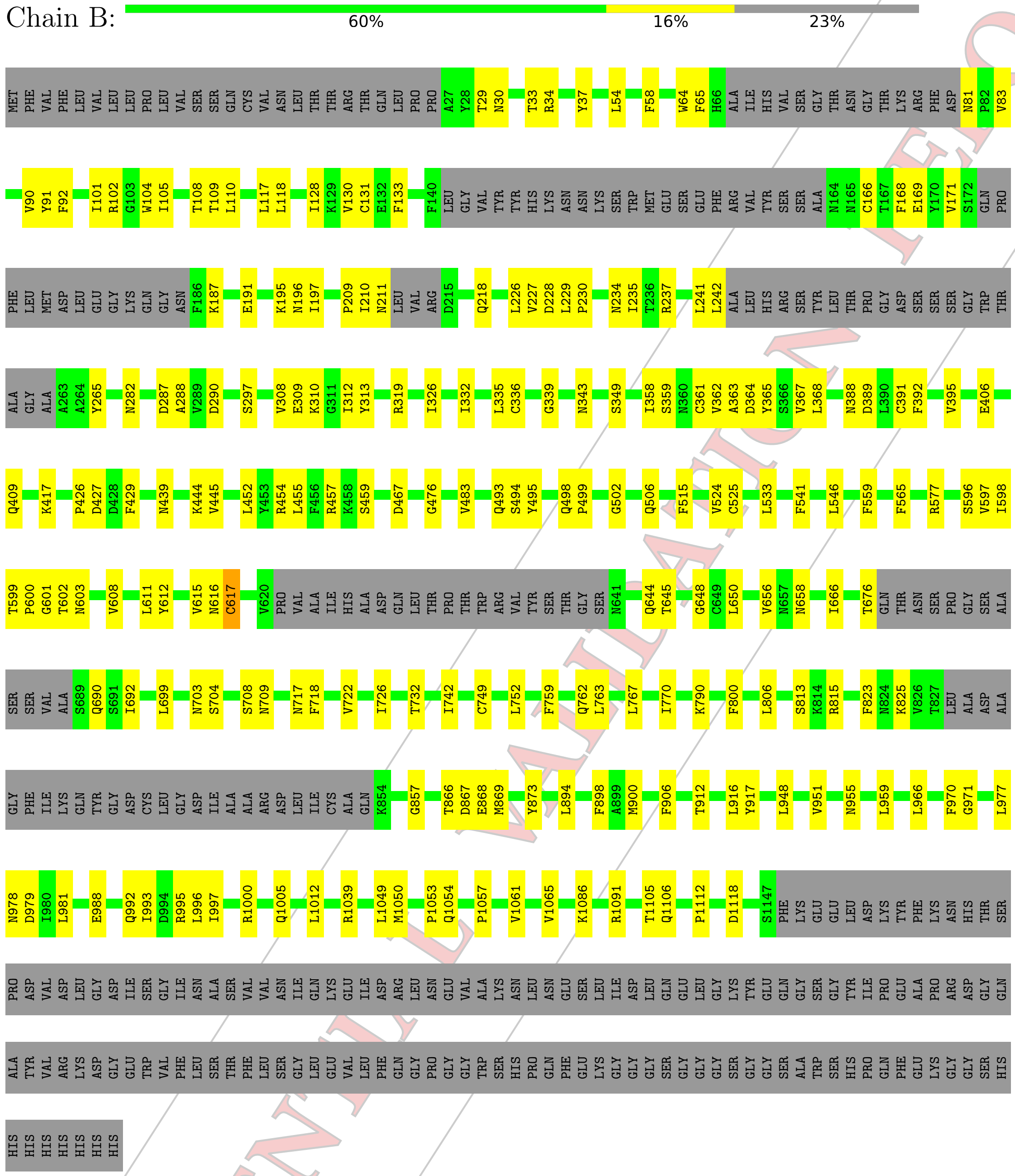

- Molecule 1: Spike glycoprotein

Chain C:

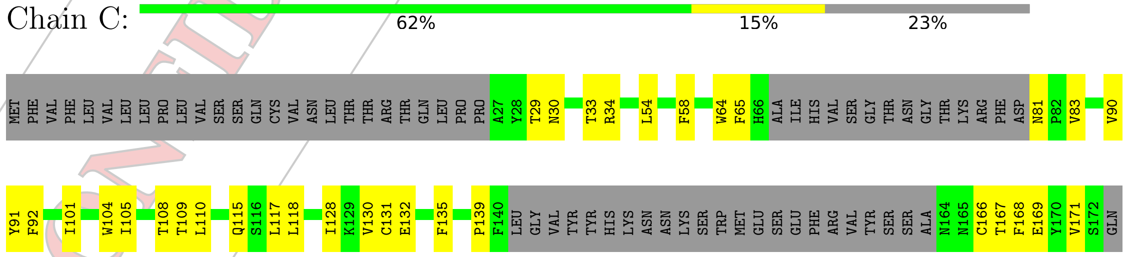

CONFIDENTIAL VALIDATION REPORT

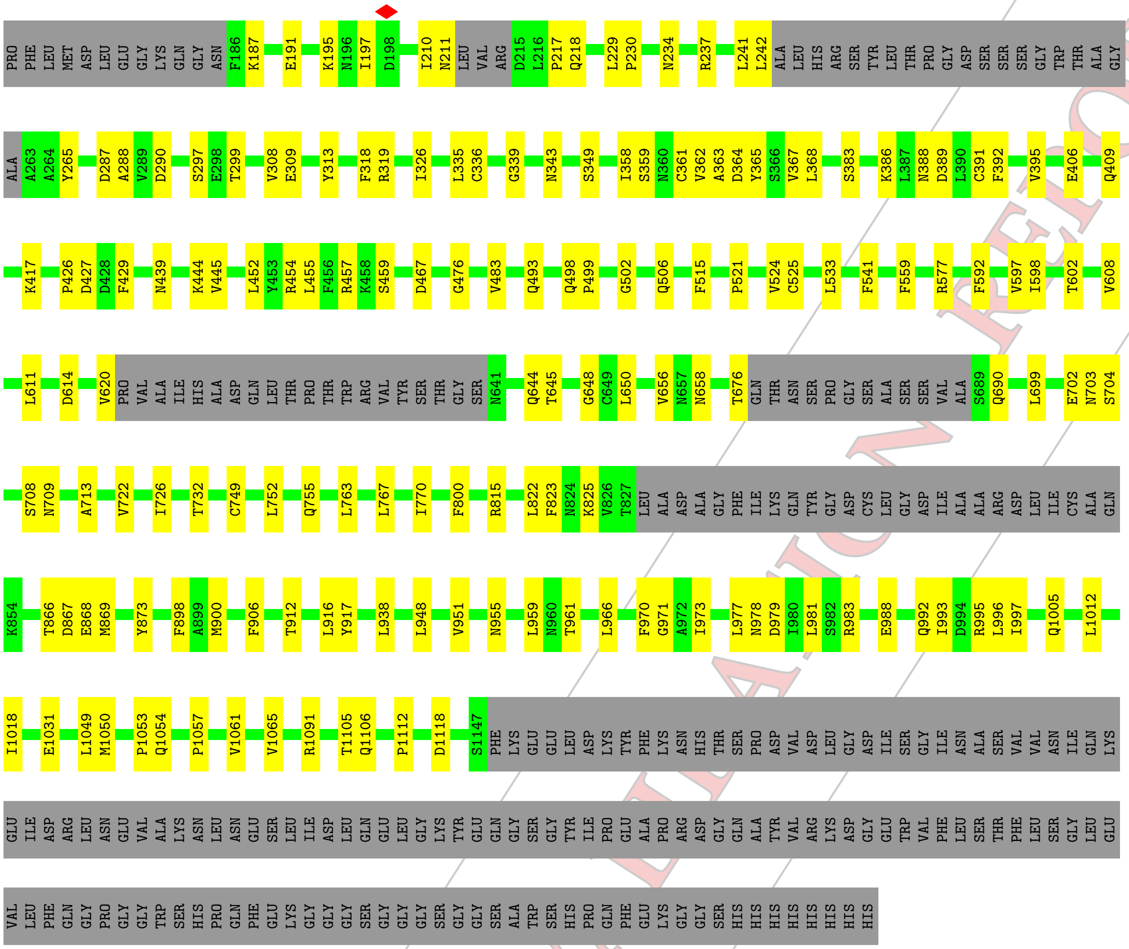

- Molecule 2: 13G9 heavy chain

Chain D:

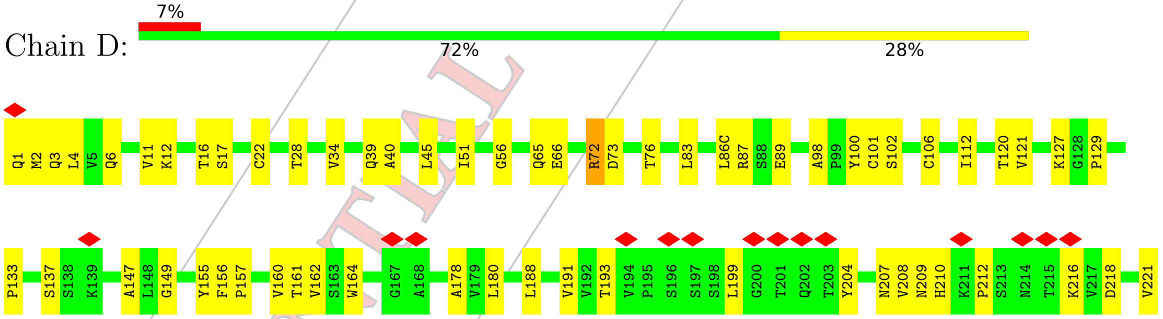

- Molecule 2: 13G9 heavy chain

Chain F:

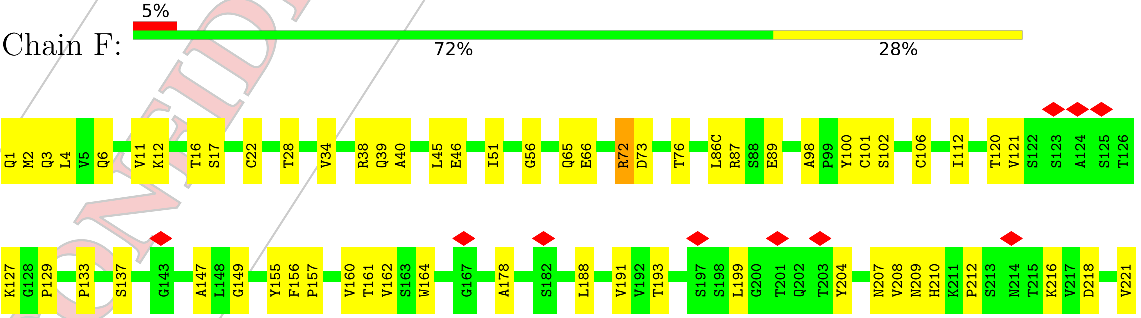

CONFIDENTIAL VALIDATION REPORT

• Molecule 2: 13G9 heavy chain

Chain H:

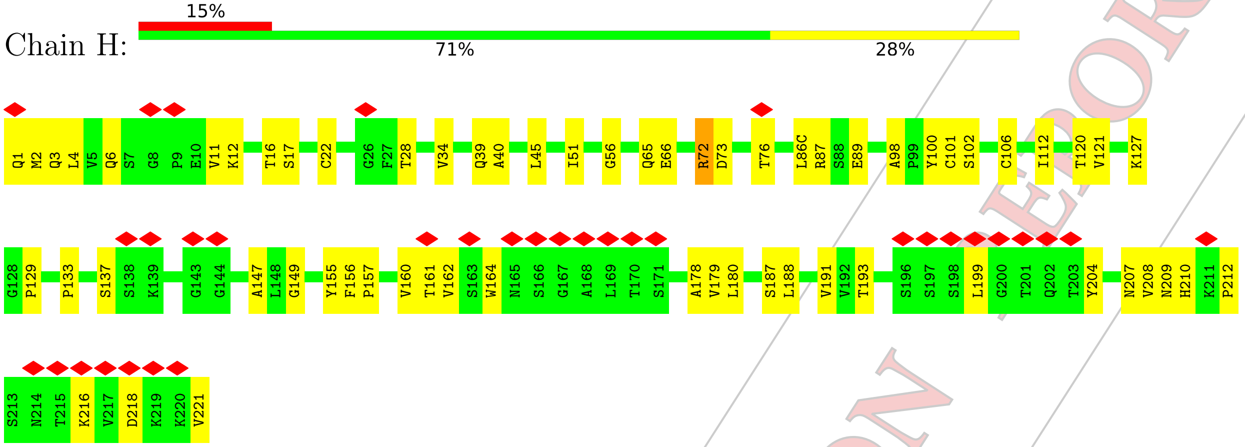

• Molecule 3: 13G9 light chain

Chain E:

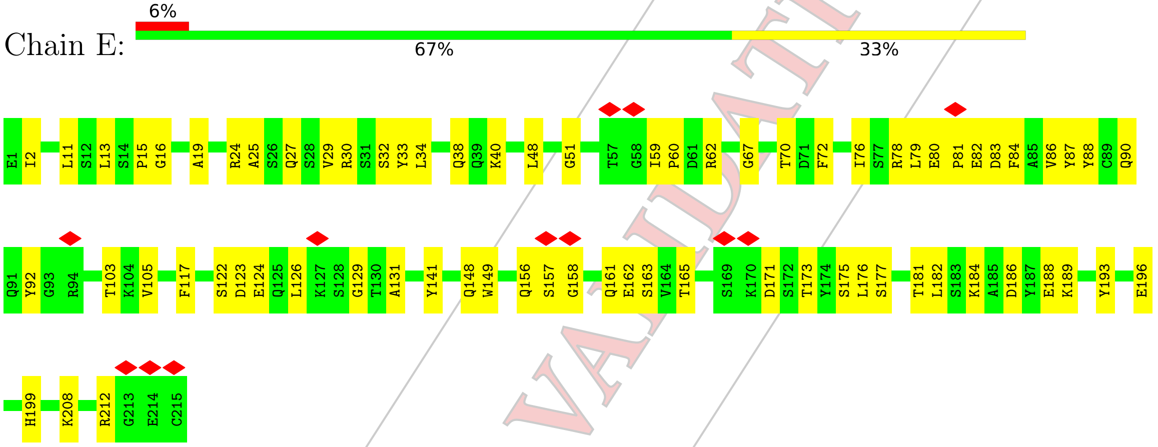

• Molecule 3: 13G9 light chain

Chain G:

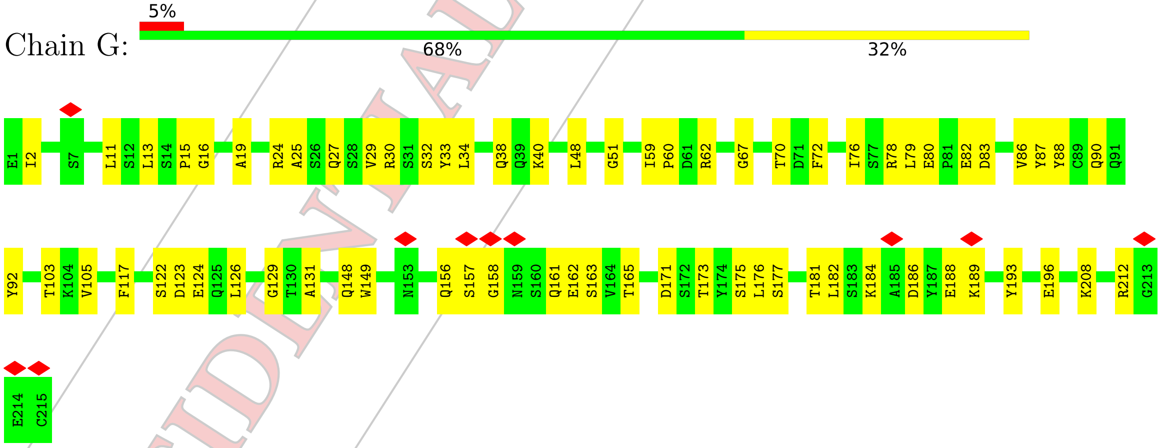

• Molecule 3: 13G9 light chain

Chain L:

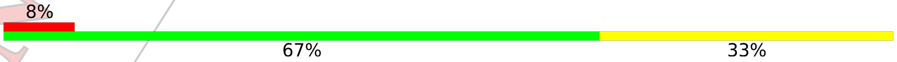

CONFIDENTIAL VALIDATION REPORT

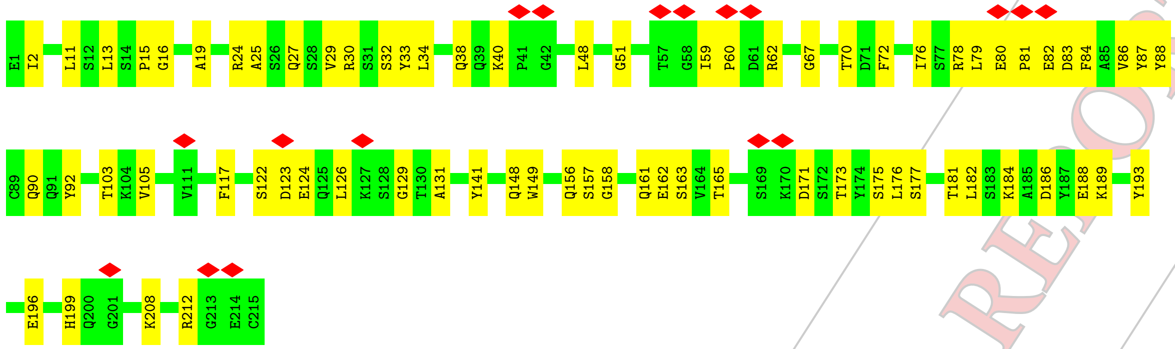

CONFIDENTIAL VALIDATION REPORT

4 Experimental information (i)

Property	Value	Source
EM reconstruction method	SINGLE PARTICLE	Depositor
Imposed symmetry	POINT, Not provided	
Number of particles used	52880	Depositor
Resolution determination method	FSC 0.143 CUT-OFF	Depositor
CTF correction method	PHASE FLIPPING AND AMPLITUDE CORRECTION	Depositor
Microscope	FEI TITAN KRIOS	Depositor
Voltage (kV)	300	Depositor
Electron dose ($e^-/\text{Å}^2$)	60	Depositor
Minimum defocus (nm)	Not provided	
Maximum defocus (nm)	Not provided	
Magnification	Not provided	
Image detector	GATAN K3 (6k x 4k)	Depositor
Maximum map value	0.805	Depositor
Minimum map value	-0.229	Depositor
Average map value	-0.000	Depositor
Map value standard deviation	0.030	Depositor
Recommended contour level	0.1042	Depositor
Map size (Å)	419.84, 419.84, 419.84	wwPDB
Map dimensions	512, 512, 512	wwPDB
Map angles (°)	90.0, 90.0, 90.0	wwPDB
Pixel spacing (Å)	0.82, 0.82, 0.82	Depositor

CONFIDENTIAL VALIDATION REPORT

5 Model quality (i)

5.1 Standard geometry (i)

Bond lengths and bond angles in the following residue types are not validated in this section: NAG

The Z score for a bond length (or angle) is the number of standard deviations the observed value is removed from the expected value. A bond length (or angle) with $|Z| > 5$ is considered an outlier worth inspection. RMSZ is the root-mean-square of all Z scores of the bond lengths (or angles).

Mol	Chain	Bond lengths		Bond angles	
		RMSZ	$\#|Z| > 5$	RMSZ	$\#|Z| > 5$
1	A	0.45	0/7763	0.53	0/10582
1	B	0.44	0/7742	0.53	0/10558
1	C	0.44	0/7747	0.53	0/10561
2	D	0.36	0/1601	0.56	0/2185
2	F	0.36	0/1601	0.56	0/2185
2	H	0.36	0/1601	0.56	0/2185
3	E	0.41	0/1631	0.57	0/2219
3	G	0.41	0/1631	0.57	0/2219
3	L	0.40	0/1631	0.57	0/2219
All	All	0.43	0/32948	0.54	0/44913

There are no bond length outliers.

There are no bond angle outliers.

There are no chirality outliers.

There are no planarity outliers.

5.2 Too-close contacts (i)

In the following table, the Non-H and H(model) columns list the number of non-hydrogen atoms and hydrogen atoms in the chain respectively. The H(added) column lists the number of hydrogen atoms added and optimized by MolProbity. The Clashes column lists the number of clashes within the asymmetric unit, whereas Symm-Clashes lists symmetry-related clashes.

Mol	Chain	Non-H	H(model)	H(added)	Clashes	Symm-Clashes
1	A	7591	0	7293	183	0
1	B	7571	0	7265	195	0
1	C	7577	0	7282	168	0
2	D	1570	0	1445	43	0

Continued on next page...

CONFIDENTIAL VALIDATION REPORT

Continued from previous page...

Mol	Chain	Non-H	H(model)	H(added)	Clashes	Symm-Clashes
2	F	1570	0	1445	41	0
2	H	1570	0	1445	42	0
3	E	1594	0	1449	54	0
3	G	1594	0	1449	55	0
3	L	1594	0	1449	54	0
4	A	112	0	104	1	0
4	B	84	0	78	3	0
4	C	112	0	104	0	0
All	All	32539	0	30808	796	0

The all-atom clashscore is defined as the number of clashes found per 1000 atoms (including hydrogen atoms). The all-atom clashscore for this structure is 13.

All (796) close contacts within the same asymmetric unit are listed below, sorted by their clash magnitude.

Atom-1	Atom-2	Interatomic distance (Å)	Clash overlap (Å)
1:A:115:GLN:HA	1:A:132:GLU:CG	1.37	1.54
1:B:282:ASN:HD21	4:B:1304:NAG:C1	1.24	1.50
1:A:115:GLN:CA	1:A:132:GLU:HG3	1.66	1.25
1:A:115:GLN:HE21	1:A:233:ILE:HG13	0.97	1.11
1:A:115:GLN:HE21	1:A:233:ILE:CG1	1.66	1.08
1:B:230:PRO:HB2	1:C:521:PRO:CG	1.85	1.06
1:A:115:GLN:CA	1:A:132:GLU:CG	2.27	1.05
1:A:115:GLN:NE2	1:A:233:ILE:HG13	1.70	1.04
1:A:115:GLN:HB3	1:A:132:GLU:OE2	1.57	1.03
1:C:115:GLN:HA	1:C:132:GLU:HG2	1.35	1.03
1:B:230:PRO:HB2	1:C:521:PRO:HG3	1.00	0.99
1:B:230:PRO:CB	1:C:521:PRO:HG3	1.93	0.97
1:A:656:VAL:HG12	1:A:658:ASN:H	1.30	0.96
1:B:656:VAL:HG12	1:B:658:ASN:H	1.30	0.94
1:C:656:VAL:HG12	1:C:658:ASN:H	1.30	0.93
1:A:521:PRO:HG3	1:C:230:PRO:HB2	1.50	0.93
1:C:815:ARG:NH1	1:C:823:PHE:CD2	2.37	0.91
1:C:135:PHE:CE1	1:C:139:PRO:HG3	2.06	0.91
1:C:115:GLN:HA	1:C:132:GLU:CG	2.01	0.90
1:A:115:GLN:HA	1:A:132:GLU:HG2	1.52	0.89
1:A:196:ASN:HD21	1:A:235:ILE:HB	1.37	0.88
1:B:310:LYS:CG	1:B:601:GLY:H	1.88	0.85
1:B:599:THR:HG22	1:B:608:VAL:HG12	1.56	0.85
1:B:598:ILE:HD12	1:B:650:LEU:HD11	1.58	0.85
1:B:310:LYS:HG2	1:B:600:PRO:HA	1.59	0.83

Continued on next page...

CONFIDENTIAL VALIDATION REPORT

Continued from previous page...

Atom-1	Atom-2	Interatomic distance (Å)	Clash overlap (Å)
1:A:115:GLN:CB	1:A:132:GLU:OE2	2.28	0.82
1:C:167:THR:O	1:C:167:THR:HG22	1.78	0.81
1:A:617:CYS:HA	1:A:620:VAL:HG23	1.63	0.80
1:B:599:THR:HG22	1:B:608:VAL:CG1	2.11	0.80
1:B:762:GLN:HE21	1:C:961:THR:HG21	1.47	0.80
1:A:197:ILE:HG22	1:A:197:ILE:O	1.83	0.79
1:A:196:ASN:HD22	1:A:235:ILE:HD12	1.48	0.79
1:C:815:ARG:NH1	1:C:823:PHE:CE2	2.52	0.78
1:A:115:GLN:HG3	1:A:233:ILE:CD1	2.14	0.77
1:B:326:ILE:HD11	1:B:533:LEU:HA	1.67	0.77
1:C:326:ILE:HD11	1:C:533:LEU:HA	1.67	0.76
3:E:48:LEU:HD13	3:E:59:ILE:HD12	1.68	0.76
1:B:196:ASN:ND2	1:B:235:ILE:HB	2.01	0.76
1:A:115:GLN:NE2	1:A:233:ILE:CG1	2.38	0.75
1:B:310:LYS:HG2	1:B:601:GLY:N	2.02	0.75
3:L:48:LEU:HD13	3:L:59:ILE:HD12	1.68	0.75
1:B:310:LYS:CG	1:B:601:GLY:N	2.48	0.74
3:G:48:LEU:HD13	3:G:59:ILE:HD12	1.68	0.74
1:A:115:GLN:HE21	1:A:233:ILE:CB	2.00	0.74
1:A:196:ASN:ND2	1:A:235:ILE:HB	2.01	0.74
1:B:597:VAL:HG12	1:B:599:THR:HG23	1.68	0.73
1:B:616:ASN:OD1	1:B:616:ASN:O	2.07	0.72
1:B:813:SER:OG	1:B:815:ARG:HG3	1.89	0.72
1:A:483:VAL:HG11	3:E:30:ARG:NH2	2.05	0.72
1:B:483:VAL:HG11	3:G:30:ARG:NH2	2.05	0.72
3:L:60:PRO:HB2	3:L:62:ARG:HG3	1.70	0.72
1:C:483:VAL:HG11	3:L:30:ARG:NH2	2.05	0.72
1:A:115:GLN:NE2	1:A:233:ILE:CB	2.52	0.71
1:B:310:LYS:HG3	1:B:601:GLY:H	1.53	0.71
1:B:226:LEU:HG	1:B:227:VAL:HG23	1.72	0.71
3:G:60:PRO:HB2	3:G:62:ARG:HG3	1.70	0.71
1:A:29:THR:HG22	1:A:30:ASN:H	1.55	0.71
1:C:318:PHE:CZ	1:C:620:VAL:HG22	2.25	0.71
3:E:60:PRO:HB2	3:E:62:ARG:HG3	1.70	0.71
1:B:29:THR:HG22	1:B:30:ASN:H	1.55	0.70
1:A:196:ASN:OD1	1:A:197:ILE:N	2.24	0.70
1:A:115:GLN:HA	1:A:132:GLU:HG3	0.71	0.70
1:B:599:THR:CG2	1:B:608:VAL:HG12	2.21	0.70
1:B:732:THR:OG1	1:B:955:ASN:ND2	2.25	0.70
1:C:732:THR:OG1	1:C:955:ASN:ND2	2.25	0.70
2:H:45:LEU:HD23	3:L:88:TYR:CE1	2.27	0.69

Continued on next page...

CONFIDENTIAL VALIDATION REPORT

Continued from previous page...

Atom-1	Atom-2	Interatomic distance (Å)	Clash overlap (Å)
1:C:130:VAL:O	1:C:130:VAL:HG12	1.93	0.69
2:F:86(C):LEU:HD21	2:F:121:VAL:HG21	1.74	0.69
1:A:732:THR:OG1	1:A:955:ASN:ND2	2.25	0.69
2:H:86(C):LEU:HD21	2:H:121:VAL:HG21	1.74	0.69
2:F:45:LEU:HD23	3:G:88:TYR:CE1	2.27	0.69
1:C:29:THR:HG22	1:C:30:ASN:H	1.55	0.69
2:D:45:LEU:HD23	3:E:88:TYR:CE1	2.27	0.68
2:D:86(C):LEU:HD21	2:D:121:VAL:HG21	1.74	0.68
3:E:67:GLY:HA2	3:E:72:PHE:CD1	2.29	0.68
1:A:326:ILE:HD11	1:A:533:LEU:HA	1.76	0.68
3:G:80:GLU:O	3:G:82:GLU:N	2.27	0.67
3:L:67:GLY:HA2	3:L:72:PHE:CD1	2.29	0.67
1:C:417:LYS:HE2	1:C:455:LEU:HD12	1.76	0.67
3:E:15:PRO:HA	3:E:79:LEU:HB2	1.76	0.67
3:L:80:GLU:O	3:L:82:GLU:N	2.27	0.67
1:B:599:THR:HG22	1:B:608:VAL:CB	2.25	0.67
3:E:80:GLU:O	3:E:82:GLU:N	2.27	0.67
3:G:15:PRO:HA	3:G:79:LEU:HB2	1.76	0.67
3:G:67:GLY:HA2	3:G:72:PHE:CD1	2.29	0.67
1:B:417:LYS:HE2	1:B:455:LEU:HD12	1.76	0.67
3:L:15:PRO:HA	3:L:79:LEU:HB2	1.76	0.67
1:A:54:LEU:HD12	1:A:195:LYS:HE3	1.77	0.67
1:A:335:LEU:HD23	1:A:362:VAL:HB	1.76	0.67
1:B:209:PRO:O	1:B:210:ILE:HD13	1.95	0.66
1:B:310:LYS:HG3	1:B:601:GLY:N	2.10	0.66
1:C:318:PHE:HZ	1:C:620:VAL:HG22	1.61	0.66
1:A:81:ASN:N	1:A:265:TYR:HH	1.94	0.66
1:A:417:LYS:HE2	1:A:455:LEU:HD12	1.76	0.66
1:B:81:ASN:N	1:B:265:TYR:HH	1.94	0.66
1:A:115:GLN:CA	1:A:132:GLU:HG2	2.15	0.66
3:L:129:GLY:HA2	3:L:184:LYS:HD3	1.77	0.66
1:A:988:GLU:OE1	1:A:988:GLU:N	2.29	0.65
1:B:598:ILE:CD1	1:B:666:ILE:HD11	2.25	0.65
1:B:988:GLU:N	1:B:988:GLU:OE1	2.30	0.65
1:C:81:ASN:N	1:C:265:TYR:HH	1.94	0.65
3:G:129:GLY:HA2	3:G:184:LYS:HD3	1.77	0.65
1:C:988:GLU:OE1	1:C:988:GLU:N	2.29	0.65
3:E:129:GLY:HA2	3:E:184:LYS:HD3	1.77	0.65
2:F:6:GLN:HG3	2:F:22:CYS:HB3	1.79	0.65
1:C:815:ARG:CZ	1:C:823:PHE:CE2	2.80	0.65
3:L:193:TYR:OH	3:L:212:ARG:NH1	2.30	0.64

Continued on next page...

CONFIDENTIAL VALIDATION REPORT

Continued from previous page...

Atom-1	Atom-2	Interatomic distance (Å)	Clash overlap (Å)
1:C:598:ILE:O	1:C:608:VAL:HB	1.97	0.64
2:F:16:THR:HG22	2:F:17:SER:H	1.62	0.64
1:B:336:CYS:HA	1:B:361:CYS:HB2	1.79	0.64
3:G:193:TYR:OH	3:G:212:ARG:NH1	2.30	0.64
2:D:6:GLN:HG3	2:D:22:CYS:HB3	1.79	0.64
3:E:193:TYR:OH	3:E:212:ARG:NH1	2.30	0.64
2:H:6:GLN:HG3	2:H:22:CYS:HB3	1.79	0.64
1:B:597:VAL:HG12	1:B:599:THR:CG2	2.28	0.64
2:D:16:THR:HG22	2:D:17:SER:H	1.62	0.64
1:B:210:ILE:HG22	1:B:211:ASN:N	2.13	0.64
1:B:598:ILE:HD11	1:B:666:ILE:HD11	1.80	0.63
1:A:617:CYS:HA	1:A:620:VAL:CG2	2.29	0.63
1:C:335:LEU:HD23	1:C:362:VAL:HB	1.80	0.63
2:H:16:THR:HG22	2:H:17:SER:H	1.62	0.63
1:A:115:GLN:HG3	1:A:233:ILE:HG13	1.80	0.63
1:B:310:LYS:HG2	1:B:600:PRO:CA	2.26	0.62
1:B:866:THR:HG22	1:B:869:MET:HG3	1.81	0.62
2:F:178:ALA:HA	2:F:188:LEU:HB3	1.81	0.62
1:B:457:ARG:NH1	1:B:459:SER:O	2.29	0.62
1:A:483:VAL:CG1	3:E:30:ARG:NH2	2.63	0.62
1:B:970:PHE:O	1:B:995:ARG:NH2	2.32	0.62
1:C:866:THR:HG22	1:C:869:MET:HG3	1.81	0.62
1:A:115:GLN:CG	1:A:233:ILE:HG13	2.29	0.62
1:C:457:ARG:NH1	1:C:459:SER:O	2.29	0.62
1:C:970:PHE:O	1:C:995:ARG:NH2	2.33	0.62
2:D:178:ALA:HA	2:D:188:LEU:HB3	1.81	0.62
1:A:644:GLN:NE2	1:A:645:THR:O	2.33	0.62
1:A:866:THR:HG22	1:A:869:MET:HG3	1.81	0.62
1:A:970:PHE:O	1:A:995:ARG:NH2	2.33	0.62
1:A:363:ALA:HB1	1:A:365:TYR:CZ	2.35	0.62
1:C:644:GLN:NE2	1:C:645:THR:O	2.33	0.62
1:A:559:PHE:HB2	1:A:577:ARG:HH21	1.65	0.61
3:E:13:LEU:HD22	3:E:19:ALA:HB2	1.82	0.61
1:A:115:GLN:HG3	1:A:233:ILE:HD11	1.82	0.61
1:B:644:GLN:NE2	1:B:645:THR:O	2.33	0.61
1:B:762:GLN:HE21	1:C:961:THR:CG2	2.12	0.61
1:C:483:VAL:CG1	3:L:30:ARG:NH2	2.63	0.61
3:E:62:ARG:NH2	3:E:83:ASP:OD2	2.33	0.61
1:B:483:VAL:CG1	3:G:30:ARG:NH2	2.63	0.61
1:C:90:VAL:HG12	1:C:92:PHE:H	1.66	0.61
1:B:559:PHE:HB2	1:B:577:ARG:HH21	1.65	0.61

Continued on next page...

CONFIDENTIAL VALIDATION REPORT

Continued from previous page...

Atom-1	Atom-2	Interatomic distance (Å)	Clash overlap (Å)
1:C:559:PHE:HB2	1:C:577:ARG:HH21	1.66	0.61
3:L:62:ARG:NH2	3:L:83:ASP:OD2	2.33	0.61
1:B:426:PRO:HG2	1:B:429:PHE:HB2	1.83	0.61
1:C:115:GLN:CA	1:C:132:GLU:HG2	2.23	0.61
2:H:127:LYS:H	2:H:156:PHE:HB3	1.66	0.61
2:H:178:ALA:HA	2:H:188:LEU:HB3	1.81	0.61
1:A:109:THR:HG22	1:A:109:THR:O	2.01	0.61
1:A:426:PRO:HG2	1:A:429:PHE:HB2	1.83	0.61
1:C:299:THR:OG1	1:C:597:VAL:HB	1.99	0.61
1:C:426:PRO:HG2	1:C:429:PHE:HB2	1.83	0.61
3:G:13:LEU:HD22	3:G:19:ALA:HB2	1.82	0.61
1:B:598:ILE:HD11	1:B:666:ILE:CD1	2.30	0.61
1:C:109:THR:O	1:C:109:THR:HG22	2.01	0.61
3:G:62:ARG:NH2	3:G:83:ASP:OD2	2.33	0.61
1:C:644:GLN:NE2	1:C:648:GLY:O	2.34	0.61
1:B:363:ALA:HB1	1:B:365:TYR:CZ	2.35	0.60
1:A:644:GLN:NE2	1:A:648:GLY:O	2.34	0.60
2:D:127:LYS:H	2:D:156:PHE:HB3	1.66	0.60
3:E:16:GLY:HA2	3:E:78:ARG:HB2	1.83	0.60
1:C:363:ALA:HB1	1:C:365:TYR:CZ	2.35	0.60
3:L:16:GLY:HA2	3:L:78:ARG:HB2	1.83	0.60
1:A:1039:ARG:NH2	1:C:1031:GLU:OE2	2.34	0.60
3:L:13:LEU:HD22	3:L:19:ALA:HB2	1.82	0.60
2:F:127:LYS:H	2:F:156:PHE:HB3	1.66	0.60
1:B:109:THR:HG22	1:B:109:THR:O	2.01	0.60
1:B:644:GLN:NE2	1:B:648:GLY:O	2.34	0.60
1:B:54:LEU:HD12	1:B:195:LYS:HE3	1.82	0.60
1:C:90:VAL:HG12	1:C:92:PHE:N	2.17	0.60
1:A:90:VAL:HG12	1:A:92:PHE:H	1.66	0.60
1:A:90:VAL:HG12	1:A:92:PHE:N	2.17	0.59
1:B:596:SER:O	1:B:611:LEU:N	2.35	0.59
1:B:599:THR:HG22	1:B:608:VAL:HB	1.84	0.59
1:C:197:ILE:O	1:C:197:ILE:HG22	2.02	0.59
1:A:457:ARG:NH1	1:A:459:SER:O	2.29	0.59
1:B:417:LYS:NZ	1:B:455:LEU:O	2.25	0.59
1:B:131:CYS:CB	1:B:133:PHE:CE2	2.86	0.58
1:A:64:TRP:HD1	1:A:65:PHE:N	2.01	0.58
1:B:363:ALA:HB2	1:B:524:VAL:HG12	1.85	0.58
1:B:310:LYS:HG2	1:B:601:GLY:H	1.60	0.58
3:G:16:GLY:HA2	3:G:78:ARG:HB2	1.83	0.58
1:A:115:GLN:HG3	1:A:233:ILE:CG1	2.33	0.58

Continued on next page...

CONFIDENTIAL VALIDATION REPORT

Continued from previous page...

Atom-1	Atom-2	Interatomic distance (Å)	Clash overlap (Å)
1:C:64:TRP:HD1	1:C:65:PHE:N	2.01	0.58
1:C:90:VAL:HG12	1:C:91:TYR:N	2.18	0.58
1:C:363:ALA:HB2	1:C:524:VAL:HG12	1.85	0.58
1:A:90:VAL:HG12	1:A:91:TYR:N	2.18	0.58
1:A:196:ASN:HA	1:A:201:PHE:HA	1.86	0.58
1:B:81:ASN:N	1:B:265:TYR:OH	2.37	0.58
1:B:873:TYR:HE1	1:C:699:LEU:HB3	1.69	0.58
1:B:971:GLY:HA3	1:B:995:ARG:HH21	1.69	0.58
1:A:195:LYS:HD2	1:A:270:LEU:CD1	2.33	0.57
1:A:335:LEU:HA	1:A:362:VAL:HB	1.86	0.57
1:A:81:ASN:N	1:A:265:TYR:OH	2.37	0.57
1:A:195:LYS:HD2	1:A:270:LEU:HD12	1.86	0.57
1:A:388:ASN:OD1	1:A:389:ASP:N	2.37	0.57
1:B:227:VAL:HG12	1:B:228:ASP:N	2.20	0.57
1:B:335:LEU:HA	1:B:362:VAL:HB	1.87	0.57
1:C:335:LEU:HA	1:C:362:VAL:HB	1.86	0.57
1:C:388:ASN:OD1	1:C:389:ASP:N	2.37	0.57
1:A:115:GLN:NE2	1:A:233:ILE:HA	2.18	0.57
1:B:64:TRP:HD1	1:B:65:PHE:N	2.01	0.57
1:B:388:ASN:OD1	1:B:389:ASP:N	2.37	0.57
1:A:971:GLY:HA3	1:A:995:ARG:HH21	1.69	0.57
3:G:161:GLN:N	3:G:161:GLN:OE1	2.38	0.57
1:C:195:LYS:CG	1:C:197:ILE:HG12	2.35	0.57
1:B:335:LEU:HD23	1:B:362:VAL:HB	1.87	0.57
1:B:90:VAL:HG12	1:B:92:PHE:H	1.70	0.56
1:C:81:ASN:N	1:C:265:TYR:OH	2.37	0.56
2:D:12:LYS:NZ	2:D:17:SER:O	2.38	0.56
1:C:363:ALA:HB1	1:C:365:TYR:CE1	2.41	0.56
2:F:157:PRO:O	2:F:210:HIS:NE2	2.33	0.56
1:A:363:ALA:HB1	1:A:365:TYR:CE1	2.41	0.56
1:B:195:LYS:HE2	1:B:197:ILE:HD11	1.87	0.56
1:C:676:THR:HA	1:C:690:GLN:HA	1.86	0.56
1:C:971:GLY:HA3	1:C:995:ARG:HH21	1.69	0.56
2:D:100:TYR:HB2	2:D:112:ILE:HD11	1.88	0.56
1:B:599:THR:CG2	1:B:608:VAL:CG1	2.79	0.56
3:E:157:SER:OG	3:E:158:GLY:N	2.39	0.56
1:A:676:THR:HA	1:A:690:GLN:HA	1.86	0.56
3:E:161:GLN:OE1	3:E:161:GLN:N	2.38	0.56
1:A:115:GLN:N	1:A:132:GLU:HG2	2.21	0.56
1:B:363:ALA:HB1	1:B:365:TYR:CE1	2.41	0.56
1:B:676:THR:HA	1:B:690:GLN:HA	1.86	0.56

Continued on next page...

CONFIDENTIAL VALIDATION REPORT

Continued from previous page...

Atom-1	Atom-2	Interatomic distance (Å)	Clash overlap (Å)
3:E:40:LYS:HE2	3:E:40:LYS:HA	1.88	0.55
1:A:33:THR:HG23	1:A:58:PHE:HE2	1.71	0.55
1:C:33:THR:HG23	1:C:58:PHE:HE2	1.71	0.55
2:F:12:LYS:NZ	2:F:17:SER:O	2.38	0.55
2:F:100:TYR:HB2	2:F:112:ILE:HD11	1.88	0.55
3:G:40:LYS:HE2	3:G:40:LYS:HA	1.89	0.55
3:G:122:SER:O	3:G:126:LEU:HG	2.07	0.55
3:L:157:SER:OG	3:L:158:GLY:N	2.39	0.55
3:L:161:GLN:N	3:L:161:GLN:OE1	2.38	0.55
3:E:122:SER:O	3:E:126:LEU:HG	2.07	0.55
2:D:207:ASN:ND2	2:D:218:ASP:OD1	2.41	0.54
1:A:983:ARG:HD2	1:A:984:LEU:HD22	1.88	0.54
3:G:157:SER:OG	3:G:158:GLY:N	2.39	0.54
3:L:40:LYS:HE2	3:L:40:LYS:HA	1.88	0.54
1:A:336:CYS:HA	1:A:361:CYS:HB2	1.90	0.54
1:C:973:ILE:HD12	1:C:983:ARG:HH21	1.73	0.54
1:B:130:VAL:HG23	1:B:168:PHE:HB3	1.88	0.54
1:C:91:TYR:OH	1:C:191:GLU:OE2	2.26	0.54
1:C:336:CYS:HA	1:C:361:CYS:HB2	1.89	0.54
3:E:40:LYS:HD3	3:E:82:GLU:OE2	2.08	0.54
2:H:100:TYR:HB2	2:H:112:ILE:HD11	1.88	0.54
1:A:108:THR:OG1	1:A:234:ASN:O	2.25	0.54
1:A:391:CYS:HB2	1:A:525:CYS:HA	1.90	0.54
1:B:33:THR:HG23	1:B:58:PHE:HE2	1.71	0.54
1:C:108:THR:OG1	1:C:234:ASN:O	2.25	0.54
2:F:207:ASN:ND2	2:F:218:ASP:OD1	2.41	0.54
1:A:210:ILE:HG12	1:A:217:PRO:HG2	1.89	0.54
1:B:308:VAL:HG22	1:B:602:THR:CG2	2.38	0.54
1:B:894:LEU:HB3	1:C:713:ALA:HB3	1.89	0.54
2:H:12:LYS:NZ	2:H:17:SER:O	2.38	0.54
2:H:207:ASN:ND2	2:H:218:ASP:OD1	2.41	0.54
1:B:108:THR:OG1	1:B:234:ASN:O	2.25	0.54
3:L:40:LYS:HD3	3:L:82:GLU:OE2	2.08	0.54
1:A:742:ILE:O	1:A:1000:ARG:NH1	2.40	0.54
2:D:157:PRO:O	2:D:210:HIS:NE2	2.33	0.54
3:L:19:ALA:HB3	3:L:76:ILE:HB	1.90	0.54
3:L:122:SER:O	3:L:126:LEU:HG	2.07	0.54
3:E:186:ASP:HA	3:E:189:LYS:HD3	1.90	0.53
1:A:599:THR:HG22	1:A:600:PRO:O	2.08	0.53
1:C:115:GLN:N	1:C:132:GLU:OE2	2.41	0.53
3:G:40:LYS:HD3	3:G:82:GLU:OE2	2.08	0.53

Continued on next page...

CONFIDENTIAL VALIDATION REPORT

Continued from previous page...

Atom-1	Atom-2	Interatomic distance (Å)	Clash overlap (Å)
1:C:167:THR:O	1:C:167:THR:CG2	2.50	0.53
3:G:186:ASP:HA	3:G:189:LYS:HD3	1.90	0.53
3:L:186:ASP:HA	3:L:189:LYS:HD3	1.89	0.53
3:E:19:ALA:HB3	3:E:76:ILE:HB	1.90	0.53
3:E:184:LYS:NZ	3:E:188:GLU:OE2	2.42	0.53
1:B:91:TYR:OH	1:B:191:GLU:OE2	2.26	0.53
1:A:326:ILE:HG22	1:A:541:PHE:HA	1.91	0.53
1:B:823:PHE:CD1	1:B:1057:PRO:HD3	2.44	0.53
1:C:210:ILE:HG12	1:C:217:PRO:HG2	1.91	0.53
1:B:90:VAL:HG12	1:B:92:PHE:N	2.23	0.53
3:G:184:LYS:NZ	3:G:188:GLU:OE2	2.42	0.53
1:B:326:ILE:HG22	1:B:541:PHE:HA	1.91	0.53
1:B:703:ASN:OD1	1:B:704:SER:N	2.42	0.53
1:A:703:ASN:OD1	1:A:704:SER:N	2.43	0.52
1:C:823:PHE:CD1	1:C:1057:PRO:HD3	2.44	0.52
1:A:309:GLU:O	1:A:313:TYR:OH	2.25	0.52
1:A:823:PHE:CD1	1:A:1057:PRO:HD3	2.44	0.52
1:C:703:ASN:OD1	1:C:704:SER:N	2.43	0.52
1:C:978:ASN:OD1	1:C:979:ASP:N	2.43	0.52
1:A:169:GLU:OE2	1:A:171:VAL:HG23	2.10	0.52
1:B:742:ILE:O	1:B:1000:ARG:NH1	2.40	0.52
1:A:349:SER:HB3	1:A:452:LEU:O	2.10	0.52
1:B:978:ASN:OD1	1:B:979:ASP:N	2.43	0.52
1:C:169:GLU:OE2	1:C:171:VAL:HG23	2.10	0.52
1:C:427:ASP:OD1	1:C:427:ASP:O	2.28	0.52
2:H:157:PRO:O	2:H:210:HIS:NE2	2.33	0.52
1:B:128:ILE:HG21	1:B:229:LEU:HD21	1.90	0.52
1:C:326:ILE:HG22	1:C:541:PHE:HA	1.91	0.52
1:C:349:SER:HB3	1:C:452:LEU:O	2.10	0.52
3:G:67:GLY:HA2	3:G:72:PHE:HD1	1.75	0.52
1:B:308:VAL:H	1:B:602:THR:CG2	2.23	0.52
3:L:184:LYS:NZ	3:L:188:GLU:OE2	2.42	0.52
1:B:169:GLU:OE2	1:B:171:VAL:HG23	2.10	0.51
1:B:1105:THR:HG22	1:B:1112:PRO:HA	1.93	0.51
1:A:128:ILE:HG21	1:A:229:LEU:HD21	1.92	0.51
1:B:427:ASP:OD1	1:B:427:ASP:O	2.28	0.51
3:G:19:ALA:HB3	3:G:76:ILE:HB	1.90	0.51
1:A:115:GLN:NE2	1:A:233:ILE:HB	2.24	0.51
1:C:195:LYS:HG3	1:C:197:ILE:HG12	1.93	0.51
1:A:1105:THR:HG22	1:A:1112:PRO:HA	1.92	0.51
3:L:165:THR:HG22	3:L:175:SER:H	1.76	0.51

Continued on next page...

CONFIDENTIAL VALIDATION REPORT

Continued from previous page...

Atom-1	Atom-2	Interatomic distance (Å)	Clash overlap (Å)
1:A:427:ASP:OD1	1:A:427:ASP:O	2.28	0.51
1:C:1105:THR:HG22	1:C:1112:PRO:HA	1.93	0.51
1:B:358:ILE:HB	1:B:395:VAL:HG13	1.93	0.51
1:A:708:SER:OG	1:A:709:ASN:N	2.43	0.51
1:C:308:VAL:H	1:C:602:THR:CG2	2.24	0.51
1:A:948:LEU:O	1:A:951:VAL:HG12	2.10	0.51
1:B:349:SER:HB3	1:B:452:LEU:O	2.10	0.51
1:B:708:SER:OG	1:B:709:ASN:N	2.43	0.51
1:C:722:VAL:HG22	1:C:1065:VAL:HG22	1.93	0.51
1:A:722:VAL:HG22	1:A:1065:VAL:HG22	1.93	0.50
1:B:857:GLY:HA2	1:C:592:PHE:HE2	1.76	0.50
1:C:708:SER:OG	1:C:709:ASN:N	2.43	0.50
3:G:165:THR:HG22	3:G:175:SER:H	1.76	0.50
2:H:210:HIS:CD2	2:H:212:PRO:HD2	2.47	0.50
1:B:948:LEU:O	1:B:951:VAL:HG12	2.10	0.50
1:C:187:LYS:HA	1:C:210:ILE:O	2.12	0.50
1:C:948:LEU:O	1:C:951:VAL:HG12	2.10	0.50
1:A:199:GLY:HA2	1:A:232:GLY:HA2	1.92	0.50
2:H:39:GLN:HB2	2:H:45:LEU:HD13	1.93	0.50
1:A:187:LYS:HA	1:A:210:ILE:O	2.12	0.50
1:B:195:LYS:CE	1:B:197:ILE:HD11	2.42	0.50
2:D:210:HIS:CD2	2:D:212:PRO:HD2	2.47	0.50
1:B:722:VAL:HG22	1:B:1065:VAL:HG22	1.93	0.50
1:A:1031:GLU:OE2	1:B:1039:ARG:NH2	2.43	0.50
1:B:187:LYS:HA	1:B:210:ILE:O	2.12	0.50
2:D:39:GLN:HB2	2:D:45:LEU:HD13	1.93	0.50
2:F:39:GLN:HB2	2:F:45:LEU:HD13	1.93	0.50
3:L:67:GLY:HA2	3:L:72:PHE:HD1	1.75	0.50
1:C:128:ILE:HG21	1:C:229:LEU:HD21	1.92	0.50
3:E:165:THR:HG22	3:E:175:SER:H	1.76	0.50
1:A:614:ASP:H	1:A:648:GLY:HA2	1.77	0.49
1:C:358:ILE:HB	1:C:395:VAL:HG13	1.93	0.49
1:B:210:ILE:HG22	1:B:211:ASN:H	1.76	0.49
2:F:210:HIS:CD2	2:F:212:PRO:HD2	2.47	0.49
1:A:210:ILE:HG13	1:A:211:ASN:H	1.77	0.49
1:B:309:GLU:O	1:B:313:TYR:OH	2.25	0.49
1:A:358:ILE:HB	1:A:395:VAL:HG13	1.93	0.49
1:A:454:ARG:NE	1:A:467:ASP:OD2	2.46	0.49
1:C:614:ASP:H	1:C:648:GLY:HA2	1.77	0.49
2:D:101:CYS:HA	2:D:106:CYS:CB	2.43	0.49
1:B:770:ILE:HD11	1:B:1012:LEU:HD23	1.95	0.49

Continued on next page...

CONFIDENTIAL VALIDATION REPORT

Continued from previous page...

Atom-1	Atom-2	Interatomic distance (Å)	Clash overlap (Å)
3:E:67:GLY:HA2	3:E:72:PHE:HD1	1.75	0.49
2:F:101:CYS:HA	2:F:106:CYS:CB	2.43	0.49
1:A:91:TYR:OH	1:A:191:GLU:OE2	2.26	0.49
2:H:101:CYS:HA	2:H:106:CYS:CB	2.43	0.49
3:L:163:SER:OG	3:L:177:SER:HB2	2.13	0.49
1:A:912:THR:OG1	1:A:1106:GLN:OE1	2.27	0.48
1:C:135:PHE:CZ	1:C:139:PRO:HG3	2.48	0.48
1:C:364:ASP:HB2	1:C:367:VAL:HB	1.95	0.48
1:C:392:PHE:CG	1:C:515:PHE:HB3	2.48	0.48
3:E:171:ASP:O	3:E:173:THR:HG23	2.13	0.48
1:C:454:ARG:NE	1:C:467:ASP:OD2	2.46	0.48
3:E:163:SER:OG	3:E:177:SER:HB2	2.13	0.48
1:A:770:ILE:HD11	1:A:1012:LEU:HD23	1.95	0.48
1:A:1091:ARG:NH1	1:A:1118:ASP:O	2.46	0.48
1:B:866:THR:CG2	1:B:869:MET:HG3	2.42	0.48
1:B:406:GLU:OE1	1:B:409:GLN:NE2	2.47	0.48
1:C:406:GLU:OE1	1:C:409:GLN:NE2	2.47	0.48
3:G:171:ASP:O	3:G:173:THR:HG23	2.13	0.48
1:A:364:ASP:HB2	1:A:367:VAL:HB	1.96	0.48
1:A:392:PHE:CG	1:A:515:PHE:HB3	2.48	0.48
1:A:749:CYS:SG	1:A:997:ILE:HD11	2.54	0.48
1:B:364:ASP:HB2	1:B:367:VAL:HB	1.96	0.48
1:B:454:ARG:NE	1:B:467:ASP:OD2	2.46	0.48
1:C:749:CYS:SG	1:C:997:ILE:HD11	2.54	0.48
1:C:770:ILE:HD11	1:C:1012:LEU:HD23	1.95	0.48
1:B:439:ASN:CB	1:B:506:GLN:HE21	2.27	0.48
1:A:90:VAL:CG1	1:A:91:TYR:N	2.77	0.48
1:B:392:PHE:CG	1:B:515:PHE:HB3	2.48	0.48
1:B:749:CYS:SG	1:B:997:ILE:HD11	2.54	0.48
1:C:90:VAL:CG1	1:C:91:TYR:N	2.76	0.48
3:L:171:ASP:O	3:L:173:THR:HG23	2.13	0.48
1:A:439:ASN:HD21	1:A:499:PRO:HA	1.79	0.48
1:A:726:ILE:HG12	1:A:1061:VAL:HG22	1.96	0.48
1:A:873:TYR:HE1	1:B:699:LEU:HD22	1.79	0.48
1:A:959:LEU:HD23	1:A:959:LEU:HA	1.66	0.48
1:C:866:THR:CG2	1:C:869:MET:HG3	2.42	0.48
1:A:759:PHE:CD2	1:A:1001:LEU:HD21	2.48	0.48
1:B:1091:ARG:NH1	1:B:1118:ASP:O	2.46	0.48
2:D:199:LEU:HA	2:D:204:TYR:HE2	1.79	0.48
2:F:133:PRO:HB3	2:F:221:VAL:HA	1.96	0.48
1:A:406:GLU:OE1	1:A:409:GLN:NE2	2.47	0.48

Continued on next page...

CONFIDENTIAL VALIDATION REPORT

Continued from previous page...

Atom-1	Atom-2	Interatomic distance (Å)	Clash overlap (Å)
1:A:866:THR:CG2	1:A:869:MET:HG3	2.42	0.48
1:B:898:PHE:HZ	1:B:1050:MET:HE1	1.77	0.48
1:C:815:ARG:NH1	1:C:823:PHE:HD2	2.09	0.48
1:C:1091:ARG:NH1	1:C:1118:ASP:O	2.46	0.48
2:F:209:ASN:ND2	2:F:216:LYS:HE3	2.29	0.48
2:H:209:ASN:HD21	2:H:216:LYS:HE3	1.79	0.48
3:L:33:TYR:HB3	3:L:92:TYR:HB2	1.96	0.48
1:B:282:ASN:HD21	4:B:1304:NAG:C2	2.14	0.47
1:B:308:VAL:CG2	1:B:602:THR:HG23	2.44	0.47
1:B:339:GLY:O	1:B:343:ASN:HB2	2.14	0.47
1:C:726:ILE:HG12	1:C:1061:VAL:HG22	1.96	0.47
2:D:209:ASN:ND2	2:D:216:LYS:HE3	2.29	0.47
2:H:133:PRO:HB3	2:H:221:VAL:HA	1.96	0.47
1:A:339:GLY:O	1:A:343:ASN:HB2	2.14	0.47
1:B:726:ILE:HG12	1:B:1061:VAL:HG22	1.96	0.47
1:C:309:GLU:O	1:C:313:TYR:OH	2.25	0.47
1:A:54:LEU:HB2	1:A:195:LYS:NZ	2.29	0.47
1:A:115:GLN:NE2	1:A:233:ILE:CA	2.77	0.47
2:H:39:GLN:HG2	2:H:40:ALA:O	2.15	0.47
1:C:195:LYS:HG2	1:C:197:ILE:HG12	1.95	0.47
2:F:39:GLN:HG2	2:F:40:ALA:O	2.15	0.47
2:F:199:LEU:HA	2:F:204:TYR:HE2	1.79	0.47
3:G:163:SER:OG	3:G:177:SER:HB2	2.13	0.47
1:B:600:PRO:HD3	1:B:692:ILE:HD11	1.95	0.47
2:D:39:GLN:HG2	2:D:40:ALA:O	2.15	0.47
1:A:34:ARG:NH2	1:A:218:GLN:O	2.48	0.47
1:C:815:ARG:HH11	1:C:823:PHE:HD2	1.62	0.47
3:E:181:THR:C	3:E:182:LEU:HD12	2.35	0.47
2:F:45:LEU:HD23	3:G:88:TYR:CZ	2.49	0.47
1:A:131:CYS:HA	1:A:166:CYS:CB	2.45	0.47
1:C:308:VAL:HG22	1:C:602:THR:CG2	2.45	0.47
1:C:365:TYR:O	1:C:368:LEU:HG	2.15	0.47
1:C:439:ASN:CB	1:C:506:GLN:HE21	2.27	0.47
2:D:209:ASN:HD21	2:D:216:LYS:HE3	1.79	0.47
3:E:33:TYR:HB3	3:E:92:TYR:HB2	1.95	0.47
3:G:33:TYR:HB3	3:G:92:TYR:HB2	1.96	0.47
2:H:199:LEU:HA	2:H:204:TYR:HE2	1.79	0.47
2:H:209:ASN:ND2	2:H:216:LYS:HE3	2.29	0.47
3:L:186:ASP:HA	3:L:189:LYS:HB2	1.97	0.47
3:E:186:ASP:HA	3:E:189:LYS:HB2	1.97	0.47
1:A:365:TYR:O	1:A:368:LEU:HG	2.15	0.47

Continued on next page...

CONFIDENTIAL VALIDATION REPORT

Continued from previous page...

Atom-1	Atom-2	Interatomic distance (Å)	Clash overlap (Å)
1:A:1053:PRO:O	1:A:1054:GLN:NE2	2.24	0.47
1:C:898:PHE:HZ	1:C:1050:MET:HE1	1.80	0.47
2:F:1:GLN:OE1	2:F:2:MET:N	2.48	0.47
2:F:209:ASN:HD21	2:F:216:LYS:HE3	1.79	0.47
1:A:29:THR:HG22	1:A:30:ASN:N	2.28	0.47
1:A:439:ASN:CB	1:A:506:GLN:HE21	2.27	0.47
1:B:308:VAL:H	1:B:602:THR:HG23	1.78	0.47
1:B:439:ASN:HD21	1:B:499:PRO:HA	1.79	0.47
1:C:439:ASN:HD21	1:C:499:PRO:HA	1.79	0.47
3:E:162:GLU:OE2	3:E:176:LEU:HB2	2.16	0.46
3:L:123:ASP:OD1	3:L:124:GLU:N	2.49	0.46
3:L:181:THR:C	3:L:182:LEU:HD12	2.35	0.46
1:B:358:ILE:O	1:B:359:SER:OG	2.34	0.46
1:B:365:TYR:O	1:B:368:LEU:HG	2.15	0.46
2:D:45:LEU:HD23	3:E:88:TYR:CZ	2.49	0.46
3:G:181:THR:C	3:G:182:LEU:HD12	2.35	0.46
1:A:130:VAL:HG12	1:A:130:VAL:O	2.15	0.46
1:A:873:TYR:CE1	1:B:699:LEU:HD22	2.51	0.46
1:A:111:ASP:OD1	1:A:135:PHE:N	2.48	0.46
1:A:115:GLN:CD	1:A:233:ILE:HG13	2.31	0.46
1:A:358:ILE:O	1:A:359:SER:OG	2.34	0.46
1:B:110:LEU:HD12	1:B:110:LEU:HA	1.81	0.46
3:G:123:ASP:OD1	3:G:124:GLU:N	2.49	0.46
2:H:45:LEU:HD23	3:L:88:TYR:CZ	2.49	0.46
1:B:227:VAL:CG1	1:B:228:ASP:N	2.79	0.46
1:B:912:THR:OG1	1:B:1106:GLN:OE1	2.26	0.46
1:C:34:ARG:NH2	1:C:218:GLN:O	2.48	0.46
1:C:339:GLY:O	1:C:343:ASN:HB2	2.14	0.46
3:G:186:ASP:HA	3:G:189:LYS:HB2	1.96	0.46
2:H:161:THR:OG1	2:H:209:ASN:HB3	2.15	0.46
1:A:101:ILE:H	1:A:101:ILE:HD12	1.80	0.46
1:A:971:GLY:HA2	1:C:755:GLN:OE1	2.16	0.46
1:B:37:TYR:OH	1:B:54:LEU:O	2.26	0.46
1:B:101:ILE:HD12	1:B:101:ILE:H	1.80	0.46
1:C:131:CYS:HA	1:C:166:CYS:CB	2.45	0.46
2:D:101:CYS:HA	2:D:106:CYS:HB3	1.98	0.46
2:F:161:THR:OG1	2:F:209:ASN:HB3	2.15	0.46
3:G:162:GLU:OE2	3:G:176:LEU:HB2	2.16	0.46
2:H:101:CYS:HA	2:H:106:CYS:HB3	1.98	0.46
1:A:676:THR:OG1	1:A:690:GLN:OE1	2.30	0.46
1:B:34:ARG:NH2	1:B:218:GLN:O	2.48	0.46

Continued on next page...

CONFIDENTIAL VALIDATION REPORT

Continued from previous page...

Atom-1	Atom-2	Interatomic distance (Å)	Clash overlap (Å)
1:C:938:LEU:HD23	1:C:938:LEU:HA	1.77	0.46
2:D:133:PRO:HB3	2:D:221:VAL:HA	1.96	0.46
2:D:161:THR:OG1	2:D:209:ASN:HB3	2.15	0.46
3:E:2:ILE:HA	3:E:27:GLN:OE1	2.16	0.46
3:E:196:GLU:N	3:E:196:GLU:OE1	2.49	0.46
1:A:502:GLY:O	1:A:506:GLN:HB2	2.16	0.46
3:E:123:ASP:OD1	3:E:124:GLU:N	2.49	0.46
2:H:1:GLN:OE1	2:H:2:MET:N	2.48	0.46
3:L:2:ILE:HA	3:L:27:GLN:OE1	2.16	0.46
1:A:115:GLN:HE21	1:A:233:ILE:CA	2.28	0.46
1:B:310:LYS:HG2	1:B:600:PRO:C	2.36	0.46
1:B:502:GLY:O	1:B:506:GLN:HB2	2.16	0.46
1:C:1053:PRO:O	1:C:1054:GLN:NE2	2.24	0.46
2:F:73:ASP:OD2	2:F:76:THR:OG1	2.29	0.46
1:C:358:ILE:O	1:C:359:SER:OG	2.34	0.46
1:C:444:LYS:HD2	1:C:445:VAL:H	1.81	0.46
1:C:763:LEU:HD21	1:C:1005:GLN:OE1	2.16	0.46
2:D:34:VAL:HG22	2:D:98:ALA:HB2	1.98	0.46
1:B:391:CYS:HB2	1:B:525:CYS:HA	1.96	0.45
2:F:101:CYS:HA	2:F:106:CYS:HB3	1.98	0.45
1:A:115:GLN:HE21	1:A:233:ILE:HA	1.80	0.45
1:A:332:ILE:HG21	1:A:527:PRO:HB3	1.98	0.45
1:A:444:LYS:HD2	1:A:445:VAL:H	1.81	0.45
1:B:763:LEU:HD21	1:B:1005:GLN:OE1	2.16	0.45
2:D:83:LEU:HD12	2:D:83:LEU:HA	1.84	0.45
3:G:196:GLU:N	3:G:196:GLU:OE1	2.49	0.45
3:L:196:GLU:N	3:L:196:GLU:OE1	2.49	0.45
1:B:210:ILE:CG2	1:B:211:ASN:N	2.78	0.45
2:D:73:ASP:OD2	2:D:76:THR:OG1	2.29	0.45
3:E:34:LEU:HD23	3:E:90:GLN:O	2.17	0.45
3:G:34:LEU:HD23	3:G:90:GLN:O	2.17	0.45
3:L:189:LYS:HB3	3:L:189:LYS:HE2	1.70	0.45
1:C:502:GLY:O	1:C:506:GLN:HB2	2.16	0.45
1:C:966:LEU:HD23	1:C:966:LEU:HA	1.82	0.45
2:H:147:ALA:HB2	2:H:193:THR:HG22	1.99	0.45
1:B:800:PHE:CD2	1:B:898:PHE:HE2	2.34	0.45
1:C:101:ILE:HD12	1:C:101:ILE:H	1.80	0.45
1:C:752:LEU:HD12	1:C:993:ILE:HG21	1.98	0.45
1:A:759:PHE:HD2	1:A:1001:LEU:HD21	1.81	0.45
1:A:763:LEU:HD21	1:A:1005:GLN:OE1	2.16	0.45
1:A:898:PHE:HZ	1:A:1050:MET:HE1	1.81	0.45

Continued on next page...

CONFIDENTIAL VALIDATION REPORT

Continued from previous page...

Atom-1	Atom-2	Interatomic distance (Å)	Clash overlap (Å)
1:B:977:LEU:HD12	1:B:977:LEU:HA	1.82	0.45
1:C:91:TYR:CG	1:C:91:TYR:O	2.70	0.45
2:F:34:VAL:HG22	2:F:98:ALA:HB2	1.98	0.45
2:H:34:VAL:HG22	2:H:98:ALA:HB2	1.98	0.45
3:L:162:GLU:OE2	3:L:176:LEU:HB2	2.15	0.45
1:A:109:THR:O	1:A:109:THR:CG2	2.65	0.45
1:A:110:LEU:HD12	1:A:110:LEU:HA	1.81	0.45
1:A:137:ASN:O	1:A:138:ASP:OD1	2.35	0.45
2:D:147:ALA:HB2	2:D:193:THR:HG22	1.99	0.45
1:A:115:GLN:CB	1:A:132:GLU:CG	2.95	0.45
1:B:90:VAL:HG12	1:B:91:TYR:N	2.32	0.45
1:B:1053:PRO:O	1:B:1054:GLN:NE2	2.24	0.45
3:L:34:LEU:HD23	3:L:90:GLN:O	2.17	0.45
1:A:752:LEU:HD12	1:A:993:ILE:HG21	1.98	0.45
1:B:195:LYS:HE2	1:B:197:ILE:CG1	2.46	0.45
1:B:196:ASN:HD22	1:B:235:ILE:HB	1.76	0.45
1:B:455:LEU:HD22	1:B:493:GLN:OE1	2.17	0.45
1:B:602:THR:OG1	1:B:603:ASN:N	2.50	0.45
1:B:752:LEU:HD12	1:B:993:ILE:HG21	1.98	0.45
1:A:800:PHE:CD2	1:A:898:PHE:HE2	2.34	0.45
1:A:1012:LEU:HD23	1:A:1012:LEU:HA	1.84	0.45
1:C:825:LYS:HA	1:C:825:LYS:HD3	1.70	0.44
2:D:1:GLN:OE1	2:D:2:MET:N	2.48	0.44
2:D:87:ARG:HD3	2:D:89:GLU:OE2	2.16	0.44
2:F:87:ARG:HD3	2:F:89:GLU:OE2	2.16	0.44
2:F:147:ALA:HB2	2:F:193:THR:HG22	1.99	0.44
3:G:29:VAL:HG12	3:G:70:THR:HG22	1.99	0.44
1:B:91:TYR:CG	1:B:91:TYR:O	2.70	0.44
1:C:498:GLN:CD	1:C:499:PRO:HD2	2.38	0.44
1:C:800:PHE:CD2	1:C:898:PHE:HE2	2.34	0.44
3:G:2:ILE:HA	3:G:27:GLN:OE1	2.16	0.44
1:A:92:PHE:HE1	1:A:265:TYR:CD2	2.35	0.44
1:B:444:LYS:HD2	1:B:445:VAL:H	1.81	0.44
1:B:598:ILE:HD13	1:B:666:ILE:HD11	1.96	0.44
3:E:131:ALA:HB3	3:E:182:LEU:HB2	1.99	0.44
3:G:149:TRP:O	3:G:156:GLN:NE2	2.51	0.44
1:A:363:ALA:HB2	1:A:524:VAL:HG12	1.99	0.44
1:C:318:PHE:CE1	1:C:620:VAL:HG13	2.53	0.44
2:F:1:GLN:OE1	2:F:2:MET:HG2	2.17	0.44
2:H:87:ARG:HD3	2:H:89:GLU:OE2	2.16	0.44
1:A:91:TYR:CG	1:A:91:TYR:O	2.70	0.44

Continued on next page...

CONFIDENTIAL VALIDATION REPORT

Continued from previous page...

Atom-1	Atom-2	Interatomic distance (Å)	Clash overlap (Å)
1:A:476:GLY:HA2	2:D:106:CYS:O	2.18	0.44
1:B:498:GLN:CD	1:B:499:PRO:HD2	2.38	0.44
1:C:92:PHE:HE1	1:C:265:TYR:CD2	2.35	0.44
3:E:149:TRP:O	3:E:156:GLN:NE2	2.51	0.44
3:L:149:TRP:O	3:L:156:GLN:NE2	2.51	0.44
1:A:699:LEU:HD22	1:C:873:TYR:HE1	1.82	0.44
1:B:92:PHE:HE1	1:B:265:TYR:CD2	2.35	0.44
1:B:476:GLY:HA2	2:F:106:CYS:O	2.18	0.44
1:C:109:THR:O	1:C:109:THR:CG2	2.65	0.44
2:F:3:GLN:O	2:F:4:LEU:HD23	2.18	0.44
1:A:498:GLN:CD	1:A:499:PRO:HD2	2.38	0.44
1:C:130:VAL:O	1:C:130:VAL:CG1	2.62	0.44
2:D:1:GLN:OE1	2:D:2:MET:HG2	2.17	0.44
3:L:131:ALA:HB3	3:L:182:LEU:HB2	1.99	0.44
1:A:455:LEU:HD22	1:A:493:GLN:OE1	2.17	0.44
1:A:737:ASP:HB3	1:A:740:MET:HB3	1.99	0.44
1:A:740:MET:HB2	1:B:319:ARG:HH21	1.82	0.44
1:A:616:ASN:CG	4:A:1307:NAG:N2	2.71	0.43
1:C:959:LEU:HD23	1:C:959:LEU:HA	1.66	0.43
3:E:24:ARG:HD2	3:E:25:ALA:H	1.83	0.43
3:G:24:ARG:HD2	3:G:25:ALA:H	1.83	0.43
2:H:1:GLN:OE1	2:H:2:MET:HG2	2.17	0.43
3:L:24:ARG:HD2	3:L:25:ALA:H	1.83	0.43
3:L:148:GLN:HG3	3:L:196:GLU:OE2	2.18	0.43
3:G:189:LYS:HE2	3:G:189:LYS:HB3	1.70	0.43
3:L:38:GLN:HB3	3:L:87:TYR:CE2	2.53	0.43
1:A:417:LYS:NZ	1:A:455:LEU:O	2.25	0.43
1:B:392:PHE:CD1	1:B:515:PHE:HB3	2.54	0.43
1:B:1086:LYS:HE2	1:B:1086:LYS:HB3	1.78	0.43
1:C:210:ILE:HG13	1:C:211:ASN:N	2.33	0.43
1:C:241:LEU:O	1:C:242:LEU:HD23	2.18	0.43
1:C:483:VAL:HG11	3:L:30:ARG:CZ	2.48	0.43
3:G:131:ALA:HB3	3:G:182:LEU:HB2	1.99	0.43
2:H:51:ILE:HD11	2:H:72:ARG:HD2	2.00	0.43
1:A:1013:ILE:HD13	1:C:1012:LEU:HB3	2.01	0.43
1:B:308:VAL:HG22	1:B:602:THR:HG23	1.99	0.43
1:B:767:LEU:HD23	1:B:767:LEU:HA	1.83	0.43
1:B:867:ASP:OD1	1:B:868:GLU:N	2.52	0.43
1:C:92:PHE:CD2	1:C:104:TRP:HZ2	2.37	0.43
1:C:455:LEU:HD22	1:C:493:GLN:OE1	2.17	0.43
1:C:476:GLY:HA2	2:H:106:CYS:O	2.18	0.43

Continued on next page...

CONFIDENTIAL VALIDATION REPORT

Continued from previous page...

Atom-1	Atom-2	Interatomic distance (Å)	Clash overlap (Å)
3:E:38:GLN:HB3	3:E:87:TYR:CE2	2.53	0.43
3:L:29:VAL:HG12	3:L:70:THR:HG22	2.00	0.43
2:F:51:ILE:HD11	2:F:72:ARG:HD2	2.00	0.43
2:H:3:GLN:O	2:H:4:LEU:HD23	2.18	0.43
2:H:73:ASP:OD2	2:H:76:THR:OG1	2.29	0.43
1:B:483:VAL:HG11	3:G:30:ARG:CZ	2.48	0.43
1:C:391:CYS:HB2	1:C:525:CYS:HA	2.01	0.43
2:D:3:GLN:O	2:D:4:LEU:HD23	2.18	0.43
3:G:148:GLN:HG3	3:G:196:GLU:OE2	2.18	0.43
1:A:92:PHE:CD2	1:A:104:TRP:HZ2	2.37	0.43
1:B:109:THR:O	1:B:109:THR:CG2	2.65	0.43
1:C:1018:ILE:HD13	1:C:1018:ILE:HA	1.88	0.43
3:E:29:VAL:HG12	3:E:70:THR:HG22	2.00	0.43
3:G:38:GLN:HB3	3:G:87:TYR:CE2	2.53	0.43
1:A:867:ASP:OD1	1:A:868:GLU:N	2.52	0.43
1:B:92:PHE:CD2	1:B:104:TRP:HZ2	2.37	0.43
1:B:241:LEU:O	1:B:242:LEU:HD23	2.18	0.43
1:C:54:LEU:CD1	1:C:197:ILE:HD11	2.48	0.43
1:C:822:LEU:HD23	1:C:822:LEU:HA	1.84	0.43
1:A:102:ARG:HD3	1:A:102:ARG:HA	1.86	0.43
1:A:483:VAL:HG11	3:E:30:ARG:CZ	2.48	0.43
1:B:332:ILE:HG22	1:B:335:LEU:HD21	2.01	0.43
1:C:392:PHE:CD1	1:C:515:PHE:HB3	2.54	0.43
3:E:148:GLN:HG3	3:E:196:GLU:OE2	2.18	0.43
1:C:867:ASP:OD1	1:C:868:GLU:N	2.52	0.43
1:A:241:LEU:O	1:A:242:LEU:HD23	2.19	0.42
2:D:51:ILE:HD11	2:D:72:ARG:HD2	2.00	0.42
3:E:32:SER:O	3:E:51:GLY:HA2	2.19	0.42
2:H:86(C):LEU:HD23	2:H:87:ARG:N	2.34	0.42
2:H:149:GLY:HA3	2:H:191:VAL:HA	2.01	0.42
2:H:160:VAL:HG12	2:H:210:HIS:HB2	2.00	0.42
1:A:767:LEU:HD23	1:A:767:LEU:HA	1.83	0.42
1:A:968:SER:HB3	1:C:755:GLN:O	2.19	0.42
1:B:981:LEU:HD23	1:B:981:LEU:HA	1.89	0.42
1:C:912:THR:OG1	1:C:1106:GLN:OE1	2.27	0.42
2:F:160:VAL:HG12	2:F:210:HIS:HB2	2.00	0.42
3:G:79:LEU:HD23	3:G:79:LEU:HA	1.88	0.42
1:A:201:PHE:HB3	1:A:229:LEU:HB2	2.02	0.42
1:A:873:TYR:CE1	1:B:699:LEU:HD13	2.54	0.42
1:B:105:ILE:HG22	1:B:110:LEU:HD11	2.02	0.42
1:B:131:CYS:HA	1:B:166:CYS:CB	2.49	0.42

Continued on next page...

CONFIDENTIAL VALIDATION REPORT

Continued from previous page...

Atom-1	Atom-2	Interatomic distance (Å)	Clash overlap (Å)
1:B:806:LEU:HD23	1:B:806:LEU:HA	1.86	0.42
2:D:160:VAL:HG12	2:D:210:HIS:HB2	2.00	0.42
2:F:86(C):LEU:HD23	2:F:87:ARG:N	2.34	0.42
3:L:86:VAL:HA	3:L:103:THR:O	2.20	0.42
1:A:335:LEU:HD23	1:A:362:VAL:CB	2.46	0.42
1:A:392:PHE:CD1	1:A:515:PHE:HB3	2.54	0.42
1:A:759:PHE:O	1:A:762:GLN:HB2	2.20	0.42
1:B:612:TYR:HB3	1:B:615:VAL:HG21	2.01	0.42
1:B:813:SER:OG	1:B:815:ARG:CG	2.64	0.42
3:E:86:VAL:HA	3:E:103:THR:O	2.20	0.42
2:F:149:GLY:HA3	2:F:191:VAL:HA	2.01	0.42
3:G:193:TYR:HH	3:G:212:ARG:NH1	2.17	0.42
1:C:290:ASP:O	1:C:297:SER:HB3	2.20	0.42
1:C:767:LEU:HD23	1:C:767:LEU:HA	1.83	0.42
1:C:971:GLY:CA	1:C:995:ARG:HH21	2.33	0.42
3:G:16:GLY:H	3:G:79:LEU:H	1.67	0.42
3:L:32:SER:O	3:L:51:GLY:HA2	2.19	0.42
1:A:290:ASP:O	1:A:297:SER:HB3	2.20	0.42
1:C:318:PHE:O	1:C:319:ARG:HG3	2.20	0.42
1:C:676:THR:OG1	1:C:690:GLN:OE1	2.30	0.42
1:C:977:LEU:HD12	1:C:977:LEU:HA	1.82	0.42
3:E:34:LEU:HD23	3:E:90:GLN:C	2.40	0.42
3:G:34:LEU:HD23	3:G:90:GLN:C	2.40	0.42
3:L:193:TYR:HE2	3:L:212:ARG:HG2	1.85	0.42
1:B:966:LEU:HD23	1:B:966:LEU:HA	1.82	0.42
3:G:86:VAL:HA	3:G:103:THR:O	2.20	0.42
3:G:181:THR:O	3:G:182:LEU:HD12	2.20	0.42
1:A:988:GLU:O	1:A:992:GLN:HG2	2.19	0.42
1:B:717:ASN:OD1	1:B:718:PHE:N	2.48	0.42
1:B:971:GLY:HA3	1:B:995:ARG:NH2	2.35	0.42
1:B:988:GLU:O	1:B:992:GLN:HG2	2.19	0.42
1:C:64:TRP:CD1	1:C:65:PHE:N	2.85	0.42
3:E:181:THR:O	3:E:182:LEU:HD12	2.20	0.42
2:F:11:VAL:HG22	2:F:120:THR:OG1	2.20	0.42
2:F:56:GLY:HA2	2:F:72:ARG:HE	1.85	0.42
1:B:196:ASN:HD21	1:B:235:ILE:HD12	1.85	0.42
1:B:227:VAL:HG12	1:B:228:ASP:H	1.84	0.42
1:B:290:ASP:O	1:B:297:SER:HB3	2.20	0.42
1:C:105:ILE:HG22	1:C:110:LEU:HD11	2.02	0.42
1:C:900:MET:HG2	1:C:917:TYR:OH	2.20	0.42
2:D:86(C):LEU:HD23	2:D:87:ARG:N	2.34	0.42

Continued on next page...

CONFIDENTIAL VALIDATION REPORT

Continued from previous page...

Atom-1	Atom-2	Interatomic distance (Å)	Clash overlap (Å)
1:B:439:ASN:HD22	1:B:506:GLN:NE2	2.18	0.42
1:B:959:LEU:HA	1:B:959:LEU:HD23	1.66	0.42
1:C:996:LEU:HD23	1:C:996:LEU:HA	1.81	0.42
3:E:193:TYR:HE2	3:E:212:ARG:HG2	1.85	0.42
3:L:34:LEU:HD23	3:L:90:GLN:C	2.40	0.42
1:A:318:PHE:O	1:A:319:ARG:HG3	2.20	0.41
1:B:83:VAL:HG21	1:B:237:ARG:HH21	1.85	0.41
2:F:28:THR:HB	2:F:102:SER:HA	2.02	0.41
3:L:33:TYR:CG	3:L:92:TYR:HB2	2.55	0.41
1:B:64:TRP:CD1	1:B:65:PHE:N	2.85	0.41
1:B:483:VAL:CG1	3:G:30:ARG:HH22	2.34	0.41
1:C:168:PHE:CG	1:C:169:GLU:N	2.88	0.41
2:D:149:GLY:HA3	2:D:191:VAL:HA	2.01	0.41
3:G:117:PHE:HA	3:G:208:LYS:HE3	2.02	0.41
3:G:193:TYR:HE2	3:G:212:ARG:HG2	1.85	0.41
1:A:83:VAL:HG21	1:A:237:ARG:HH21	1.85	0.41
1:A:973:ILE:HD12	1:A:983:ARG:NH2	2.35	0.41
1:B:312:ILE:HD11	1:B:596:SER:HB3	2.02	0.41
3:G:32:SER:O	3:G:51:GLY:HA2	2.19	0.41
3:L:11:LEU:HB3	3:L:105:VAL:HG22	2.02	0.41
2:D:28:THR:HB	2:D:102:SER:HA	2.02	0.41
2:D:65:GLN:HG3	2:D:66:GLU:HG3	2.02	0.41
2:F:38:ARG:NE	2:F:46:GLU:OE2	2.50	0.41
1:A:900:MET:HG2	1:A:917:TYR:OH	2.20	0.41
1:A:906:PHE:CD2	1:A:916:LEU:HB2	2.56	0.41
1:B:759:PHE:O	1:B:763:LEU:HG	2.20	0.41
2:F:129:PRO:HA	2:F:155:TYR:HB3	2.03	0.41
2:F:162:VAL:HG13	2:F:208:VAL:HG22	2.02	0.41
1:B:900:MET:HG2	1:B:917:TYR:OH	2.20	0.41
1:B:1049:LEU:HA	1:B:1049:LEU:HD23	1.79	0.41
1:C:287:ASP:OD1	1:C:288:ALA:N	2.54	0.41
1:C:1012:LEU:HD23	1:C:1012:LEU:HA	1.84	0.41
2:D:11:VAL:HG22	2:D:120:THR:OG1	2.20	0.41
2:D:56:GLY:HA2	2:D:72:ARG:HE	1.85	0.41
2:H:56:GLY:HA2	2:H:72:ARG:HE	1.85	0.41
1:B:611:LEU:HD12	1:B:650:LEU:HD13	2.03	0.41
1:B:971:GLY:CA	1:B:995:ARG:HH21	2.33	0.41
1:C:906:PHE:CD2	1:C:916:LEU:HB2	2.56	0.41
1:C:971:GLY:HA3	1:C:995:ARG:NH2	2.35	0.41
2:H:65:GLN:HG3	2:H:66:GLU:HG3	2.02	0.41
3:L:16:GLY:H	3:L:79:LEU:H	1.67	0.41

Continued on next page...

CONFIDENTIAL VALIDATION REPORT

Continued from previous page...

Atom-1	Atom-2	Interatomic distance (Å)	Clash overlap (Å)
1:A:168:PHE:CG	1:A:169:GLU:N	2.88	0.41
1:A:611:LEU:HD12	1:A:650:LEU:HD13	2.03	0.41
1:B:168:PHE:CG	1:B:169:GLU:N	2.89	0.41
1:B:790:LYS:NZ	1:C:702:GLU:HG3	2.36	0.41
1:C:611:LEU:HD12	1:C:650:LEU:HD13	2.03	0.41
1:C:988:GLU:O	1:C:992:GLN:HG2	2.19	0.41
2:D:162:VAL:HG13	2:D:208:VAL:HG22	2.02	0.41
3:E:38:GLN:HG2	3:E:48:LEU:HD21	2.03	0.41
1:A:117:LEU:HD23	1:A:118:LEU:N	2.36	0.41
1:A:328:ARG:HA	1:A:328:ARG:HD2	1.95	0.41
1:A:439:ASN:HD22	1:A:506:GLN:NE2	2.18	0.41
1:A:1086:LYS:HE2	1:A:1086:LYS:HB3	1.78	0.41
1:B:102:ARG:HA	1:B:102:ARG:HD3	1.86	0.41
1:B:494:SER:OG	1:B:495:TYR:N	2.54	0.41
1:B:596:SER:HB2	1:B:611:LEU:HB3	2.02	0.41
1:B:615:VAL:HG12	1:B:617:CYS:H	1.86	0.41
1:C:117:LEU:HD23	1:C:118:LEU:N	2.36	0.41
1:C:383:SER:HB2	1:C:386:LYS:HB2	2.03	0.41
1:C:1049:LEU:HD23	1:C:1049:LEU:HA	1.79	0.41
2:D:129:PRO:HA	2:D:155:TYR:HB3	2.03	0.41
3:E:11:LEU:HB3	3:E:105:VAL:HG22	2.03	0.41
3:E:33:TYR:CG	3:E:92:TYR:HB2	2.55	0.41
2:H:11:VAL:HG22	2:H:120:THR:OG1	2.20	0.41
2:H:129:PRO:HA	2:H:155:TYR:HB3	2.03	0.41
3:L:181:THR:O	3:L:182:LEU:HD12	2.20	0.41
1:A:126:VAL:O	1:A:128:ILE:HG13	2.21	0.41
1:A:129:LYS:HD2	1:A:133:PHE:HZ	1.86	0.41
1:B:227:VAL:CG1	1:B:228:ASP:H	2.33	0.41
1:B:825:LYS:HD3	1:B:825:LYS:HA	1.70	0.41
1:C:83:VAL:HG21	1:C:237:ARG:HH21	1.85	0.41
1:C:336:CYS:HA	1:C:361:CYS:CB	2.51	0.41
1:C:815:ARG:CZ	1:C:823:PHE:HE2	2.31	0.41
2:F:65:GLN:HG3	2:F:66:GLU:HG3	2.02	0.41
3:G:33:TYR:CG	3:G:92:TYR:HB2	2.55	0.41
2:H:162:VAL:HG13	2:H:208:VAL:HG22	2.02	0.41
1:B:565:PHE:N	1:B:565:PHE:CD1	2.89	0.40
1:C:417:LYS:NZ	1:C:455:LEU:O	2.25	0.40
3:E:81:PRO:HA	3:E:84:PHE:HE1	1.86	0.40
3:E:117:PHE:HA	3:E:208:LYS:HE3	2.02	0.40
2:H:28:THR:HB	2:H:102:SER:HA	2.02	0.40
2:H:149:GLY:HA2	2:H:164:TRP:CZ2	2.57	0.40

Continued on next page...

CONFIDENTIAL VALIDATION REPORT

Continued from previous page...

Atom-1	Atom-2	Interatomic distance (Å)	Clash overlap (Å)
3:L:38:GLN:HG2	3:L:48:LEU:HD21	2.03	0.40
1:A:1141:LEU:HD12	1:A:1141:LEU:HA	1.94	0.40
1:B:287:ASP:OD1	1:B:288:ALA:N	2.54	0.40
1:B:906:PHE:CD2	1:B:916:LEU:HB2	2.56	0.40
1:B:996:LEU:HA	1:B:996:LEU:HD23	1.81	0.40
1:C:110:LEU:HA	1:C:110:LEU:HD12	1.81	0.40
3:E:141:TYR:O	3:E:199:HIS:NE2	2.54	0.40
3:G:38:GLN:HG2	3:G:48:LEU:HD21	2.03	0.40
3:L:81:PRO:HA	3:L:84:PHE:HE1	1.86	0.40
1:A:383:SER:HB2	1:A:386:LYS:HB2	2.03	0.40
1:B:282:ASN:ND2	4:B:1304:NAG:C2	2.76	0.40
1:C:439:ASN:HD22	1:C:506:GLN:NE2	2.18	0.40
1:C:906:PHE:HE1	1:C:1049:LEU:HD11	1.86	0.40
2:D:149:GLY:HA2	2:D:164:TRP:CZ2	2.57	0.40
2:D:156:PHE:HA	2:D:157:PRO:HA	1.84	0.40
2:D:180:LEU:HD23	2:D:180:LEU:H	1.86	0.40
3:E:79:LEU:HD23	3:E:79:LEU:HA	1.88	0.40
3:G:11:LEU:HB3	3:G:105:VAL:HG22	2.02	0.40
2:H:179:VAL:N	2:H:187:SER:O	2.38	0.40
1:A:83:VAL:HG21	1:A:237:ARG:NH2	2.37	0.40
1:A:105:ILE:HG22	1:A:110:LEU:HD11	2.02	0.40
1:A:106:PHE:HB2	1:A:117:LEU:HB3	2.04	0.40
1:A:717:ASN:OD1	1:A:718:PHE:N	2.48	0.40
1:B:546:LEU:HD23	1:B:546:LEU:H	1.87	0.40
3:E:16:GLY:H	3:E:79:LEU:H	1.67	0.40
2:F:149:GLY:HA2	2:F:164:TRP:CZ2	2.57	0.40
3:L:117:PHE:HA	3:L:208:LYS:HE3	2.02	0.40
3:L:141:TYR:O	3:L:199:HIS:NE2	2.55	0.40
1:B:117:LEU:HD23	1:B:118:LEU:N	2.36	0.40
1:B:210:ILE:CG2	1:B:211:ASN:H	2.35	0.40
1:C:308:VAL:O	1:C:602:THR:HG23	2.22	0.40
1:C:981:LEU:HD23	1:C:981:LEU:HA	1.89	0.40
2:H:180:LEU:H	2:H:180:LEU:HD23	1.86	0.40

There are no symmetry-related clashes.

CONFIDENTIAL VALIDATION REPORT

5.3 Torsion angles (i)

5.3.1 Protein backbone (i)

In the following table, the Percentiles column shows the percent Ramachandran outliers of the chain as a percentile score with respect to all PDB entries followed by that with respect to all EM entries.

The Analysed column shows the number of residues for which the backbone conformation was analysed, and the total number of residues.

Mol	Chain	Analysed	Favoured	Allowed	Outliers	Percentiles	
1	A	972/1288 (76%)	891 (92%)	80 (8%)	1 (0%)	51	84
1	B	972/1288 (76%)	893 (92%)	79 (8%)	0	100	100
1	C	972/1288 (76%)	894 (92%)	78 (8%)	0	100	100
2	D	219/221 (99%)	204 (93%)	14 (6%)	1 (0%)	29	67
2	F	219/221 (99%)	204 (93%)	14 (6%)	1 (0%)	29	67
2	H	219/221 (99%)	204 (93%)	14 (6%)	1 (0%)	29	67
3	E	213/215 (99%)	195 (92%)	18 (8%)	0	100	100
3	G	213/215 (99%)	195 (92%)	18 (8%)	0	100	100
3	L	213/215 (99%)	194 (91%)	19 (9%)	0	100	100
All	All	4212/5172 (81%)	3874 (92%)	334 (8%)	4 (0%)	54	84

All (4) Ramachandran outliers are listed below:

Mol	Chain	Res	Type
1	A	200	TYR
2	D	137	SER
2	F	137	SER
2	H	137	SER

5.3.2 Protein sidechains (i)

In the following table, the Percentiles column shows the percent sidechain outliers of the chain as a percentile score with respect to all PDB entries followed by that with respect to all EM entries.

The Analysed column shows the number of residues for which the sidechain conformation was analysed, and the total number of residues.

CONFIDENTIAL VALIDATION REPORT

Mol	Chain	Analysed	Rotameric	Outliers	Percentiles	
1	A	825/1112 (74%)	825 (100%)	0	100	100
1	B	821/1112 (74%)	820 (100%)	1 (0%)	93	97
1	C	822/1112 (74%)	822 (100%)	0	100	100
2	D	158/188 (84%)	157 (99%)	1 (1%)	86	91
2	F	158/188 (84%)	157 (99%)	1 (1%)	86	91
2	H	158/188 (84%)	157 (99%)	1 (1%)	86	91
3	E	162/186 (87%)	162 (100%)	0	100	100
3	G	162/186 (87%)	162 (100%)	0	100	100
3	L	162/186 (87%)	162 (100%)	0	100	100
All	All	3428/4458 (77%)	3424 (100%)	4 (0%)	93	97

All (4) residues with a non-rotameric sidechain are listed below:

Mol	Chain	Res	Type
1	B	617	CYS
2	D	72	ARG
2	F	72	ARG
2	H	72	ARG

Sometimes sidechains can be flipped to improve hydrogen bonding and reduce clashes. All (11) such sidechains are listed below:

Mol	Chain	Res	Type
1	A	115	GLN
1	A	506	GLN
1	A	955	ASN
1	B	196	ASN
1	B	282	ASN
1	B	506	GLN
1	B	616	ASN
1	B	762	GLN
1	B	955	ASN
1	C	506	GLN
1	C	955	ASN

5.3.3 RNA (i)

There are no RNA molecules in this entry.

CONFIDENTIAL VALIDATION REPORT

5.4 Non-standard residues in protein, DNA, RNA chains (i)

There are no non-standard protein/DNA/RNA residues in this entry.

5.5 Carbohydrates (i)

There are no monosaccharides in this entry.

5.6 Ligand geometry (i)

22 ligands are modelled in this entry.

In the following table, the Counts columns list the number of bonds (or angles) for which Mogul statistics could be retrieved, the number of bonds (or angles) that are observed in the model and the number of bonds (or angles) that are defined in the Chemical Component Dictionary. The Link column lists molecule types, if any, to which the group is linked. The Z score for a bond length (or angle) is the number of standard deviations the observed value is removed from the expected value. A bond length (or angle) with $|Z| > 2$ is considered an outlier worth inspection. RMSZ is the root-mean-square of all Z scores of the bond lengths (or angles).

Mol	Type	Chain	Res	Link	Bond lengths			Bond angles		
					Counts	RMSZ	$\#\|Z\|>2$	Counts	RMSZ	$\#\|Z\|>2$
4	NAG	B	1302	1	14,14,15	0.27	0	17,19,21	0.35	0
4	NAG	B	1306	1	14,14,15	0.27	0	17,19,21	0.41	0
4	NAG	C	1307	1	14,14,15	0.30	0	17,19,21	0.59	1 (5%)
4	NAG	A	1303	1	14,14,15	0.40	0	17,19,21	0.92	1 (5%)
4	NAG	B	1305	1	14,14,15	0.20	0	17,19,21	0.46	0
4	NAG	A	1306	1	14,14,15	0.20	0	17,19,21	0.46	0
4	NAG	A	1301	1	14,14,15	0.43	0	17,19,21	1.81	4 (23%)
4	NAG	A	1307	1	14,14,15	0.29	0	17,19,21	0.60	1 (5%)
4	NAG	B	1304	1	14,14,15	0.20	0	17,19,21	0.58	0
4	NAG	C	1301	1	14,14,15	0.30	0	17,19,21	0.78	1 (5%)
4	NAG	A	1305	1	14,14,15	0.34	0	17,19,21	1.21	1 (5%)
4	NAG	C	1303	1	14,14,15	0.39	0	17,19,21	0.99	1 (5%)
4	NAG	B	1303	1	14,14,15	0.23	0	17,19,21	0.45	0
4	NAG	A	1302	1	14,14,15	0.19	0	17,19,21	0.49	0
4	NAG	C	1305	1	14,14,15	0.40	0	17,19,21	1.41	2 (11%)
4	NAG	C	1302	1	14,14,15	0.31	0	17,19,21	0.80	1 (5%)
4	NAG	A	1304	1	14,14,15	0.23	0	17,19,21	0.45	0
4	NAG	C	1306	1	14,14,15	0.20	0	17,19,21	0.46	0
4	NAG	B	1301	1	14,14,15	0.29	0	17,19,21	0.79	1 (5%)
4	NAG	C	1304	1	14,14,15	0.40	0	17,19,21	0.78	0

CONFIDENTIAL VALIDATION REPORT

Mol	Type	Chain	Res	Link	Bond lengths			Bond angles		
					Counts	RMSZ	#|Z| > 2	Counts	RMSZ	#|Z| > 2
4	NAG	C	1308	1	14,14,15	0.29	0	17,19,21	0.40	0
4	NAG	A	1308	1	14,14,15	0.29	0	17,19,21	0.41	0

In the following table, the Chirals column lists the number of chiral outliers, the number of chiral centers analysed, the number of these observed in the model and the number defined in the Chemical Component Dictionary. Similar counts are reported in the Torsion and Rings columns. '-' means no outliers of that kind were identified.

Mol	Type	Chain	Res	Link	Chirals	Torsions	Rings
4	NAG	B	1302	1	-	0/6/23/26	0/1/1/1
4	NAG	B	1306	1	-	2/6/23/26	0/1/1/1
4	NAG	C	1307	1	-	2/6/23/26	0/1/1/1
4	NAG	A	1303	1	-	2/6/23/26	0/1/1/1
4	NAG	B	1305	1	-	2/6/23/26	0/1/1/1
4	NAG	A	1306	1	-	2/6/23/26	0/1/1/1
4	NAG	A	1301	1	-	5/6/23/26	0/1/1/1
4	NAG	A	1307	1	-	2/6/23/26	0/1/1/1
4	NAG	B	1304	1	-	3/6/23/26	0/1/1/1
4	NAG	C	1301	1	-	1/6/23/26	0/1/1/1
4	NAG	A	1305	1	-	3/6/23/26	0/1/1/1
4	NAG	C	1303	1	-	4/6/23/26	0/1/1/1
4	NAG	B	1303	1	-	1/6/23/26	0/1/1/1
4	NAG	A	1302	1	-	1/6/23/26	0/1/1/1
4	NAG	C	1305	1	-	2/6/23/26	0/1/1/1
4	NAG	C	1302	1	-	2/6/23/26	0/1/1/1
4	NAG	A	1304	1	-	1/6/23/26	0/1/1/1
4	NAG	C	1306	1	-	2/6/23/26	0/1/1/1
4	NAG	B	1301	1	-	1/6/23/26	0/1/1/1
4	NAG	C	1304	1	-	0/6/23/26	0/1/1/1
4	NAG	C	1308	1	-	2/6/23/26	0/1/1/1
4	NAG	A	1308	1	-	2/6/23/26	0/1/1/1

There are no bond length outliers.

All (14) bond angle outliers are listed below:

Mol	Chain	Res	Type	Atoms	Z	Observed(^{o})	Ideal(^{o})
4	A	1301	NAG	C1-O5-C5	4.71	118.58	112.19
4	A	1301	NAG	O5-C1-C2	3.86	117.39	111.29

Continued on next page...

CONFIDENTIAL VALIDATION REPORT

Continued from previous page...

Mol	Chain	Res	Type	Atoms	Z	Observed(^{o})	Ideal(^{o})
4	C	1305	NAG	C2-N2-C7	3.84	128.37	122.90
4	A	1305	NAG	C2-N2-C7	3.12	127.34	122.90
4	B	1301	NAG	C1-O5-C5	2.65	115.78	112.19
4	C	1301	NAG	C1-O5-C5	2.60	115.72	112.19
4	A	1301	NAG	C4-C3-C2	-2.60	107.20	111.02
4	C	1303	NAG	O5-C1-C2	-2.45	107.42	111.29
4	A	1303	NAG	C1-O5-C5	-2.33	109.03	112.19
4	C	1305	NAG	O7-C7-N2	2.24	126.08	121.95
4	A	1301	NAG	C1-C2-N2	2.09	114.06	110.49
4	A	1307	NAG	C1-O5-C5	2.04	114.96	112.19
4	C	1307	NAG	C1-O5-C5	2.04	114.95	112.19
4	C	1302	NAG	O5-C1-C2	-2.00	108.12	111.29

There are no chirality outliers.

All (42) torsion outliers are listed below:

Mol	Chain	Res	Type	Atoms
4	A	1301	NAG	C8-C7-N2-C2
4	A	1301	NAG	O7-C7-N2-C2
4	A	1305	NAG	C3-C2-N2-C7
4	A	1305	NAG	C8-C7-N2-C2
4	A	1305	NAG	O7-C7-N2-C2
4	A	1301	NAG	C4-C5-C6-O6
4	C	1302	NAG	C8-C7-N2-C2
4	C	1302	NAG	O7-C7-N2-C2
4	C	1303	NAG	C8-C7-N2-C2
4	B	1304	NAG	C4-C5-C6-O6
4	A	1301	NAG	O5-C5-C6-O6
4	A	1301	NAG	C1-C2-N2-C7
4	A	1303	NAG	C8-C7-N2-C2
4	C	1303	NAG	O7-C7-N2-C2
4	A	1306	NAG	O5-C5-C6-O6
4	B	1305	NAG	O5-C5-C6-O6
4	C	1306	NAG	O5-C5-C6-O6
4	B	1304	NAG	O5-C5-C6-O6
4	A	1303	NAG	O7-C7-N2-C2
4	C	1308	NAG	O5-C5-C6-O6
4	A	1308	NAG	O5-C5-C6-O6
4	B	1306	NAG	O5-C5-C6-O6
4	C	1303	NAG	O5-C5-C6-O6
4	A	1306	NAG	C4-C5-C6-O6
4	B	1305	NAG	C4-C5-C6-O6

Continued on next page...

CONFIDENTIAL VALIDATION REPORT

Continued from previous page...

Mol	Chain	Res	Type	Atoms
4	C	1306	NAG	C4-C5-C6-O6
4	A	1307	NAG	C4-C5-C6-O6
4	C	1307	NAG	C4-C5-C6-O6
4	A	1304	NAG	O5-C5-C6-O6
4	A	1307	NAG	O5-C5-C6-O6
4	B	1303	NAG	O5-C5-C6-O6
4	C	1307	NAG	O5-C5-C6-O6
4	C	1303	NAG	C4-C5-C6-O6
4	B	1301	NAG	C3-C2-N2-C7
4	B	1304	NAG	C3-C2-N2-C7
4	C	1301	NAG	C3-C2-N2-C7
4	C	1305	NAG	C3-C2-N2-C7
4	B	1306	NAG	C4-C5-C6-O6
4	C	1308	NAG	C4-C5-C6-O6
4	A	1308	NAG	C4-C5-C6-O6
4	C	1305	NAG	C1-C2-N2-C7
4	A	1302	NAG	C4-C5-C6-O6

There are no ring outliers.

2 monomers are involved in 4 short contacts:

Mol	Chain	Res	Type	Clashes	Symm-Clashes
4	A	1307	NAG	1	0
4	B	1304	NAG	3	0

The following is a two-dimensional graphical depiction of Mogul quality analysis of bond lengths, bond angles, torsion angles, and ring geometry for all instances of the Ligand of Interest. In addition, ligands with molecular weight > 250 and outliers as shown on the validation Tables will also be included. For torsion angles, if less then 5% of the Mogul distribution of torsion angles is within 10 degrees of the torsion angle in question, then that torsion angle is considered an outlier. Any bond that is central to one or more torsion angles identified as an outlier by Mogul will be highlighted in the graph. For rings, the root-mean-square deviation (RMSD) between the ring in question and similar rings identified by Mogul is calculated over all ring torsion angles. If the average RMSD is greater than 60 degrees and the minimal RMSD between the ring in question and any Mogul-identified rings is also greater than 60 degrees, then that ring is considered an outlier. The outliers are highlighted in purple. The color gray indicates Mogul did not find sufficient equivalents in the CSD to analyse the geometry.

CONFIDENTIAL VALIDATION REPORT

Ligand NAG B 1302

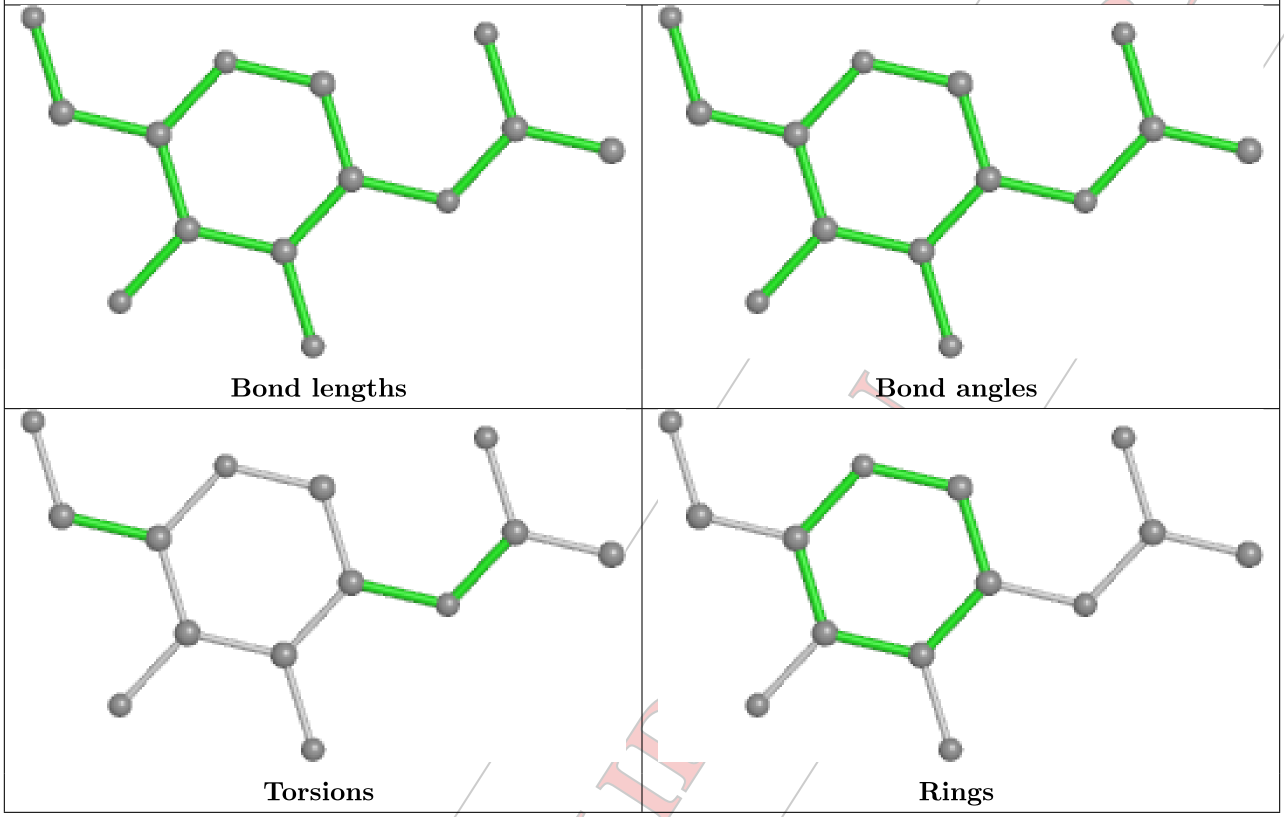

Ligand NAG B 1306

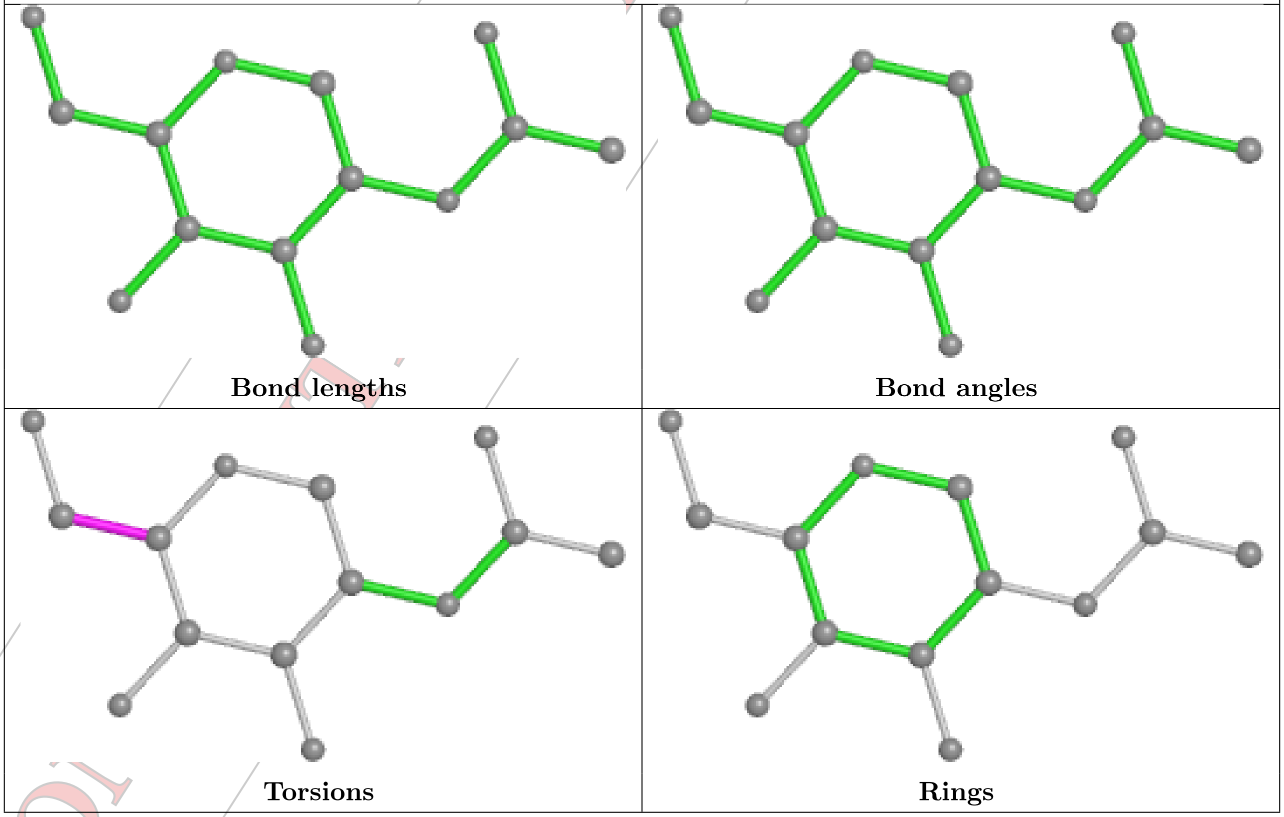

Ligand NAG C 1307

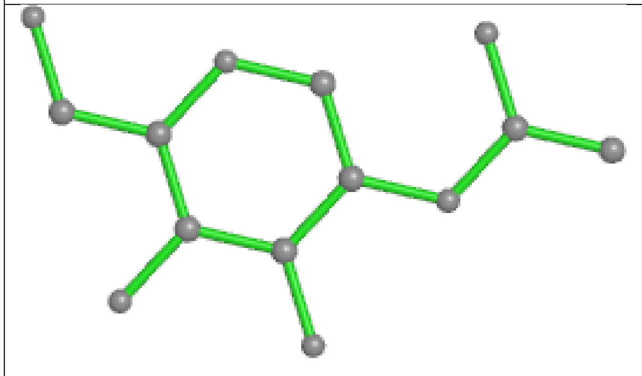

Bond lengths

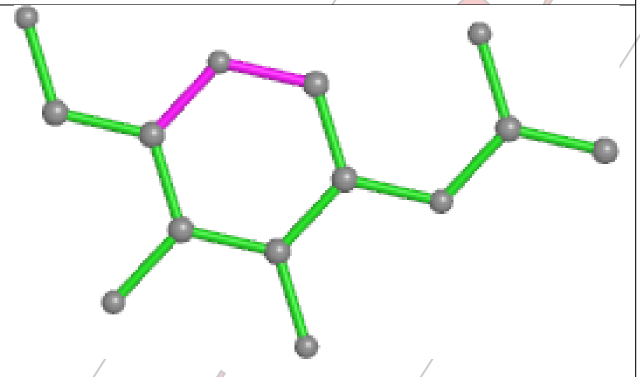

Bond angles

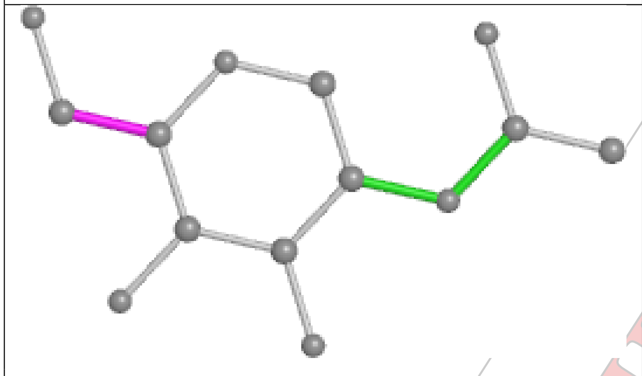

Torsions

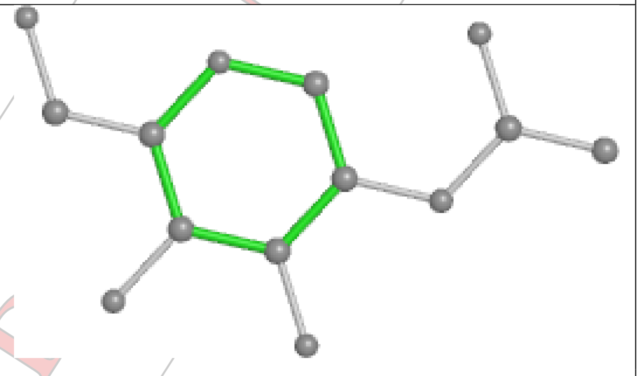

Rings

Ligand NAG A 1303

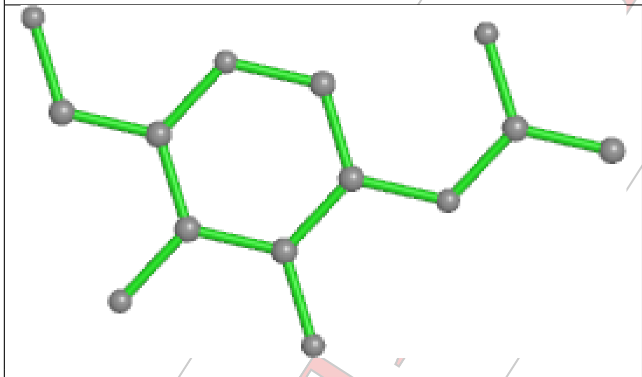

Bond lengths

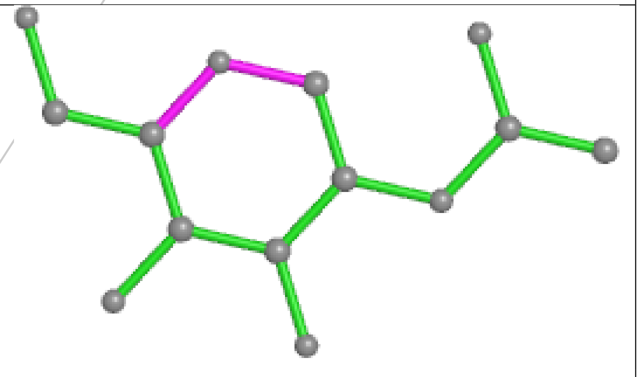

Bond angles

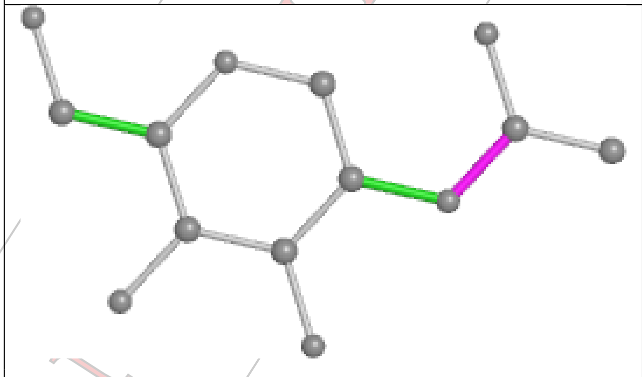

Torsions

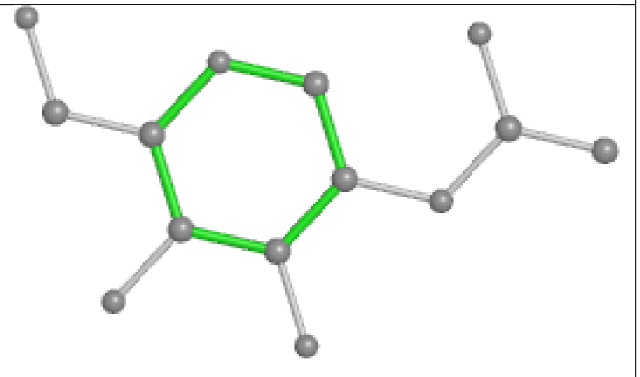

Rings

Ligand NAG B 1305

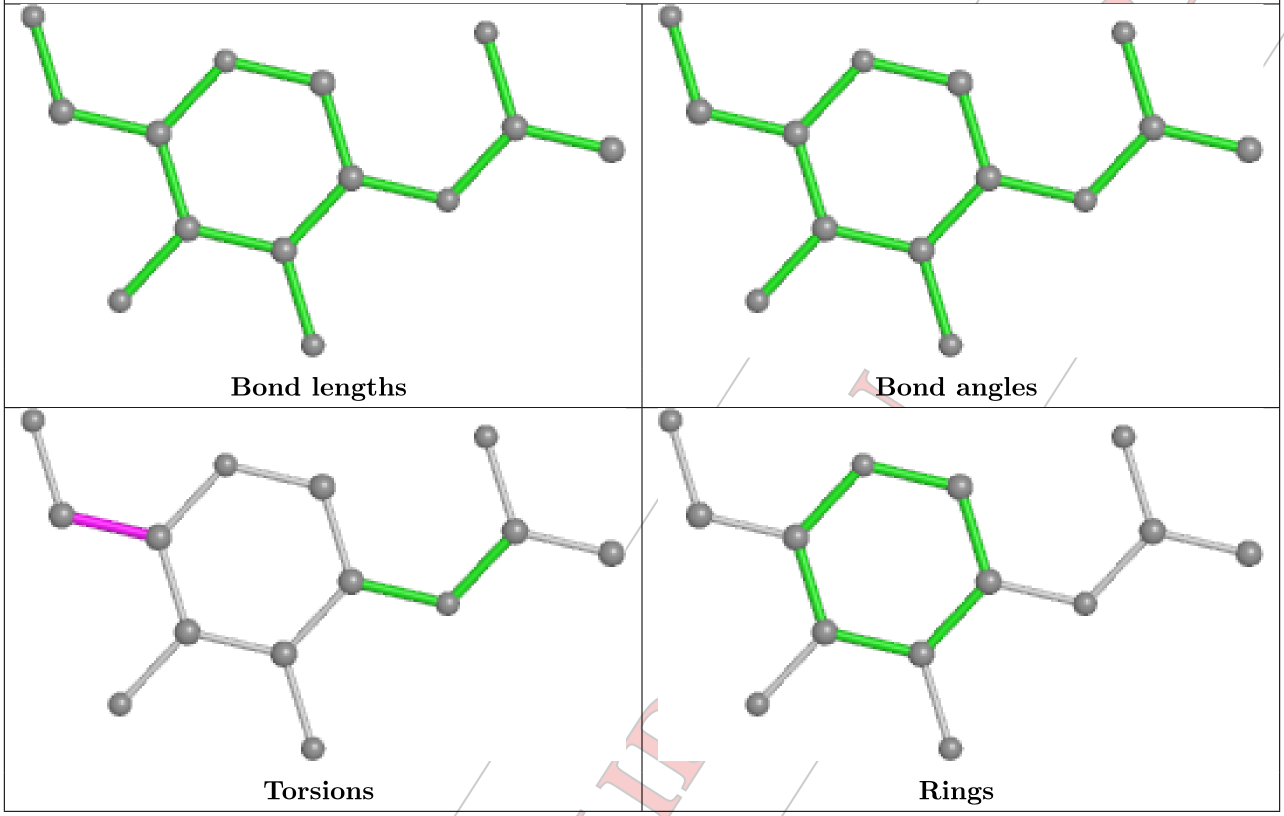

Ligand NAG A 1306

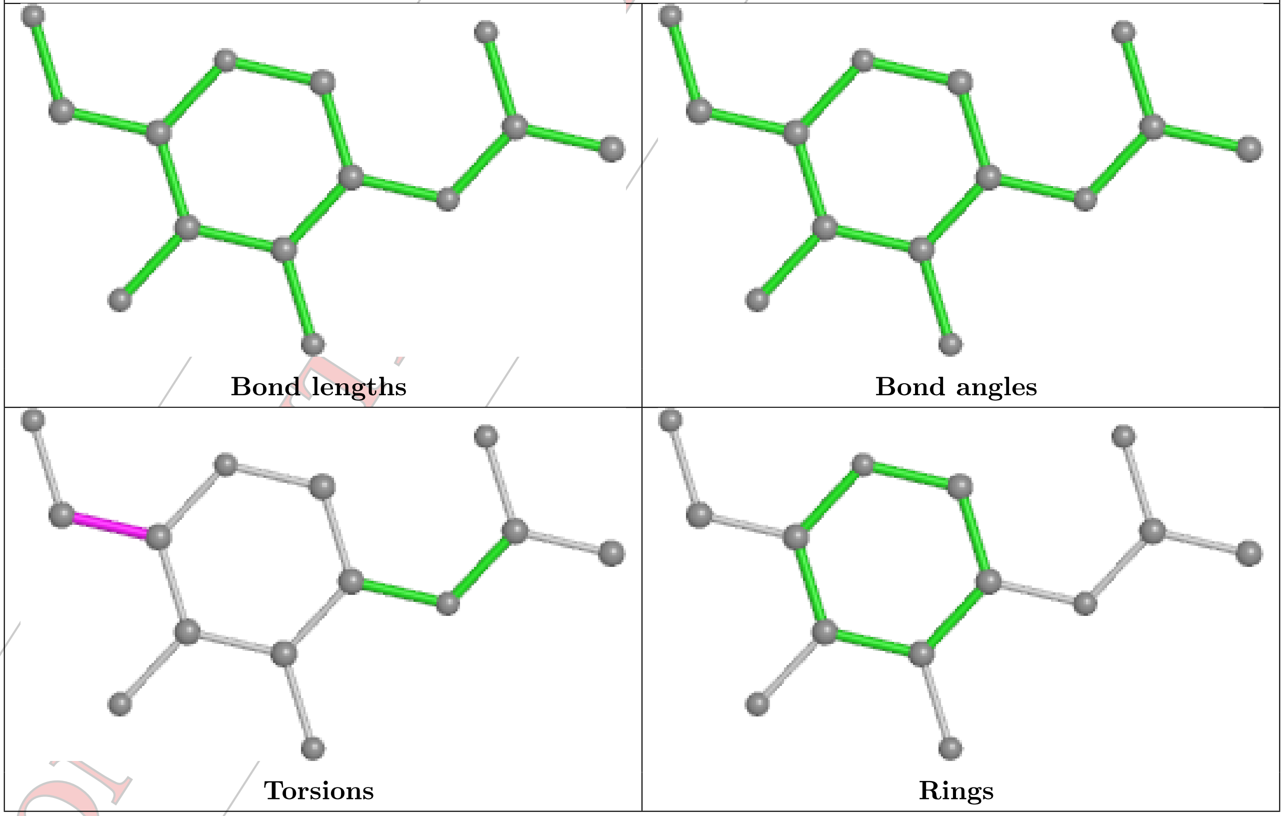

Ligand NAG A 1301

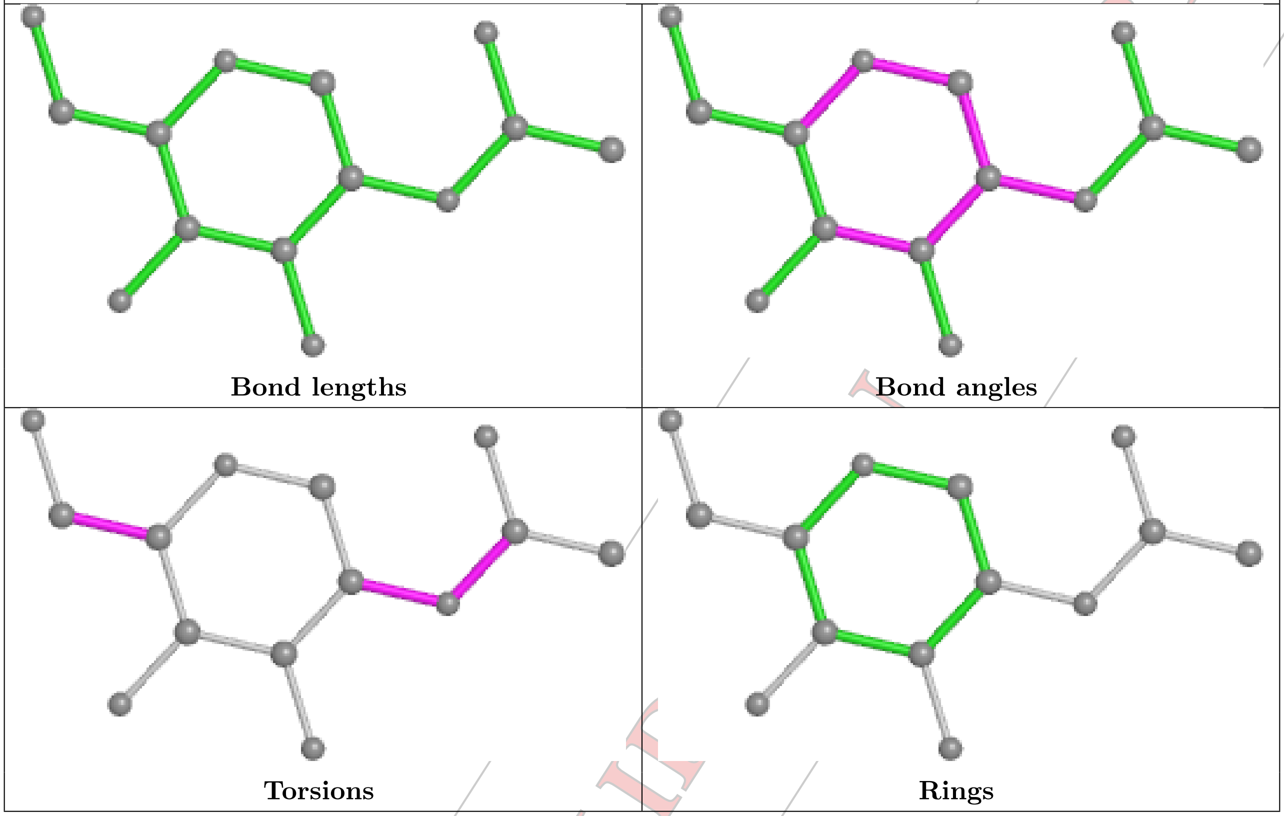

Ligand NAG A 1307

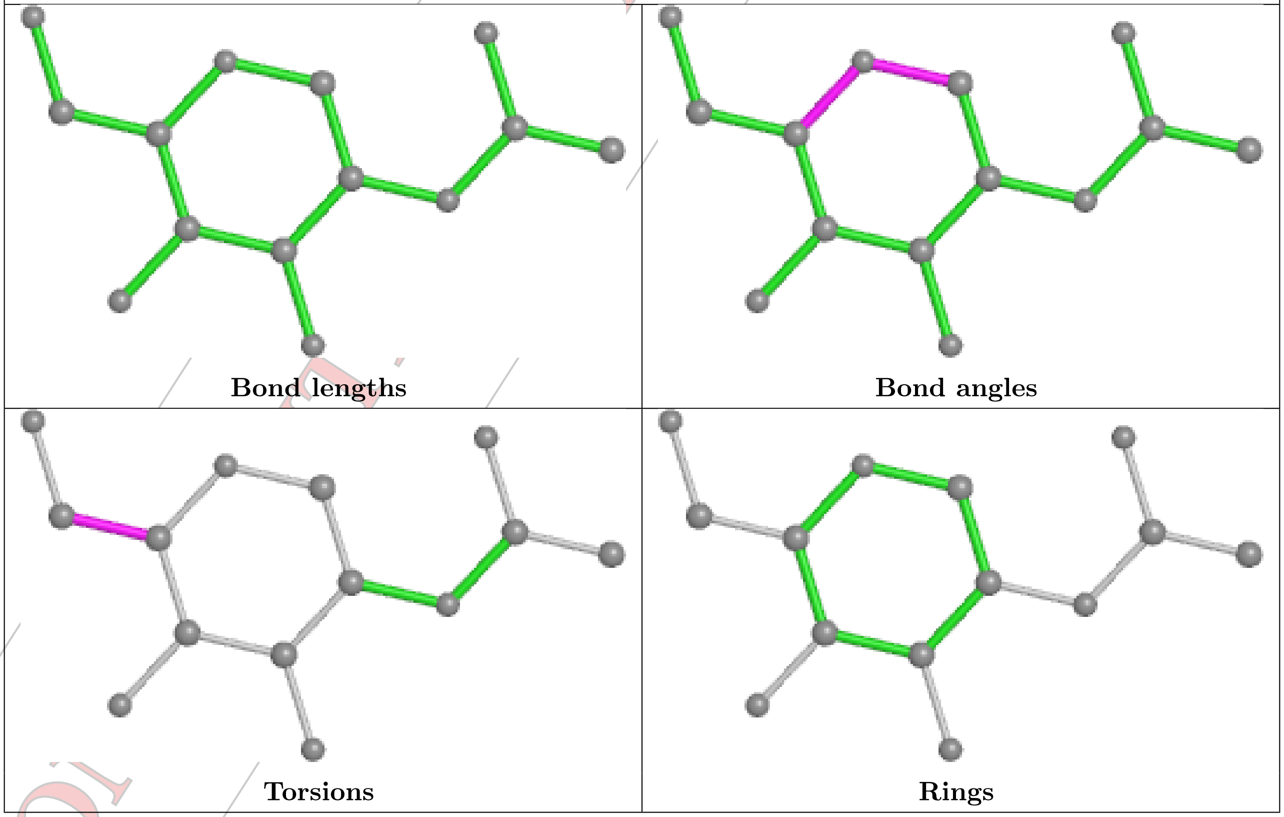

Ligand NAG B 1304

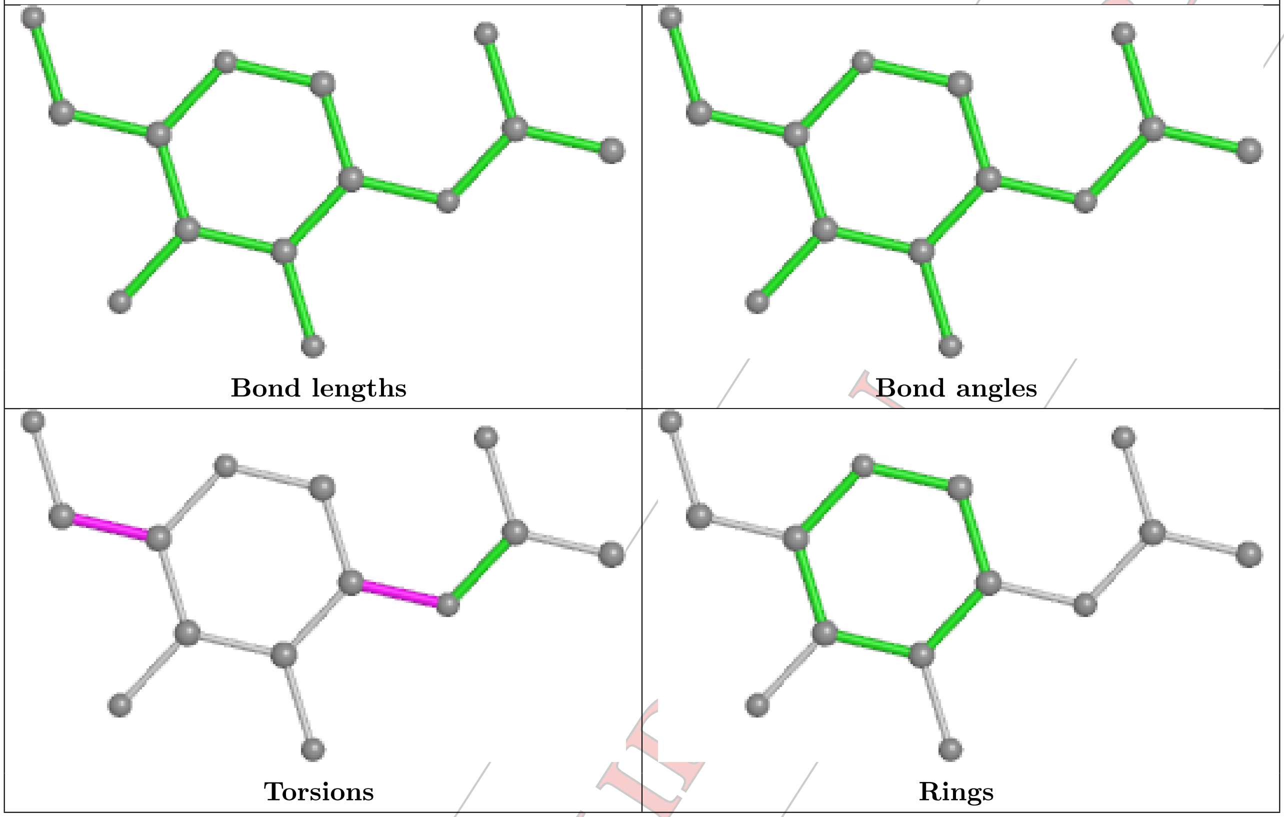

Ligand NAG C 1301

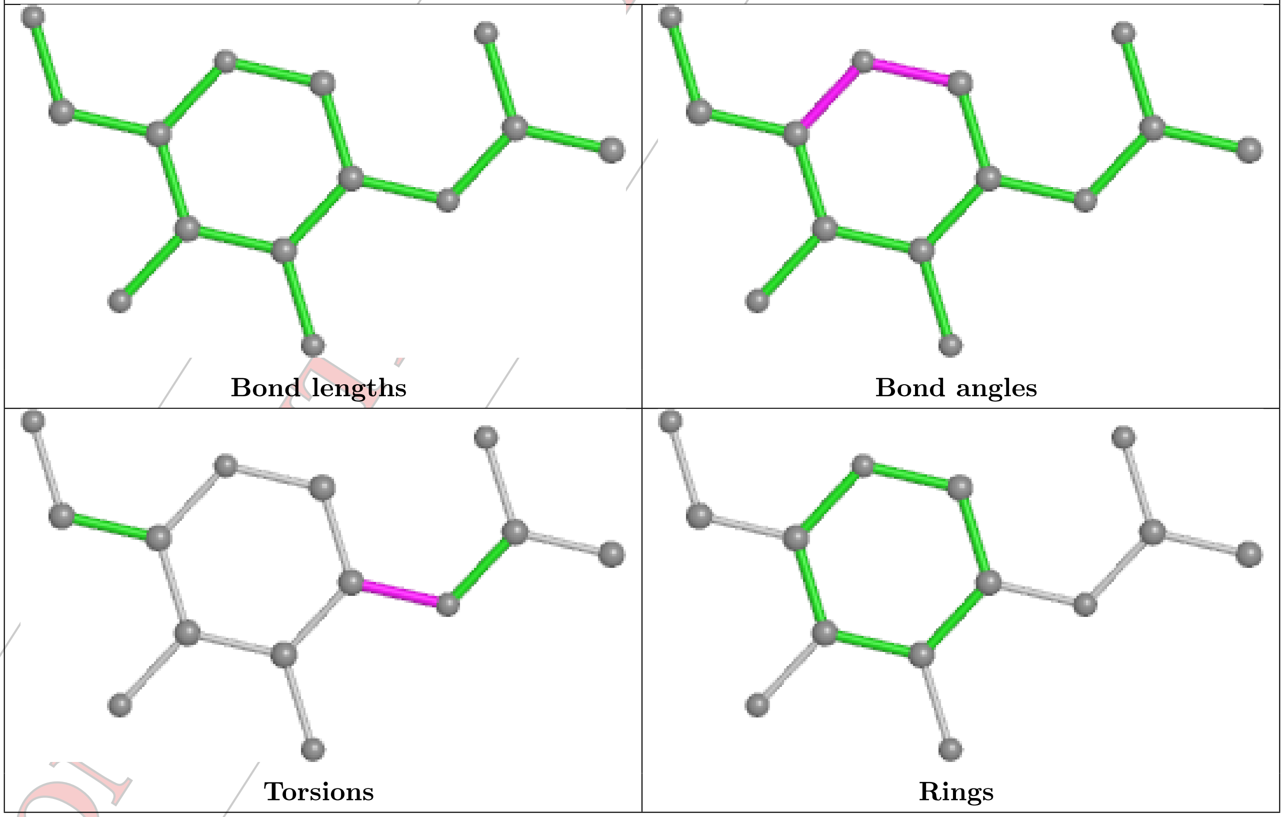

Ligand NAG A 1305

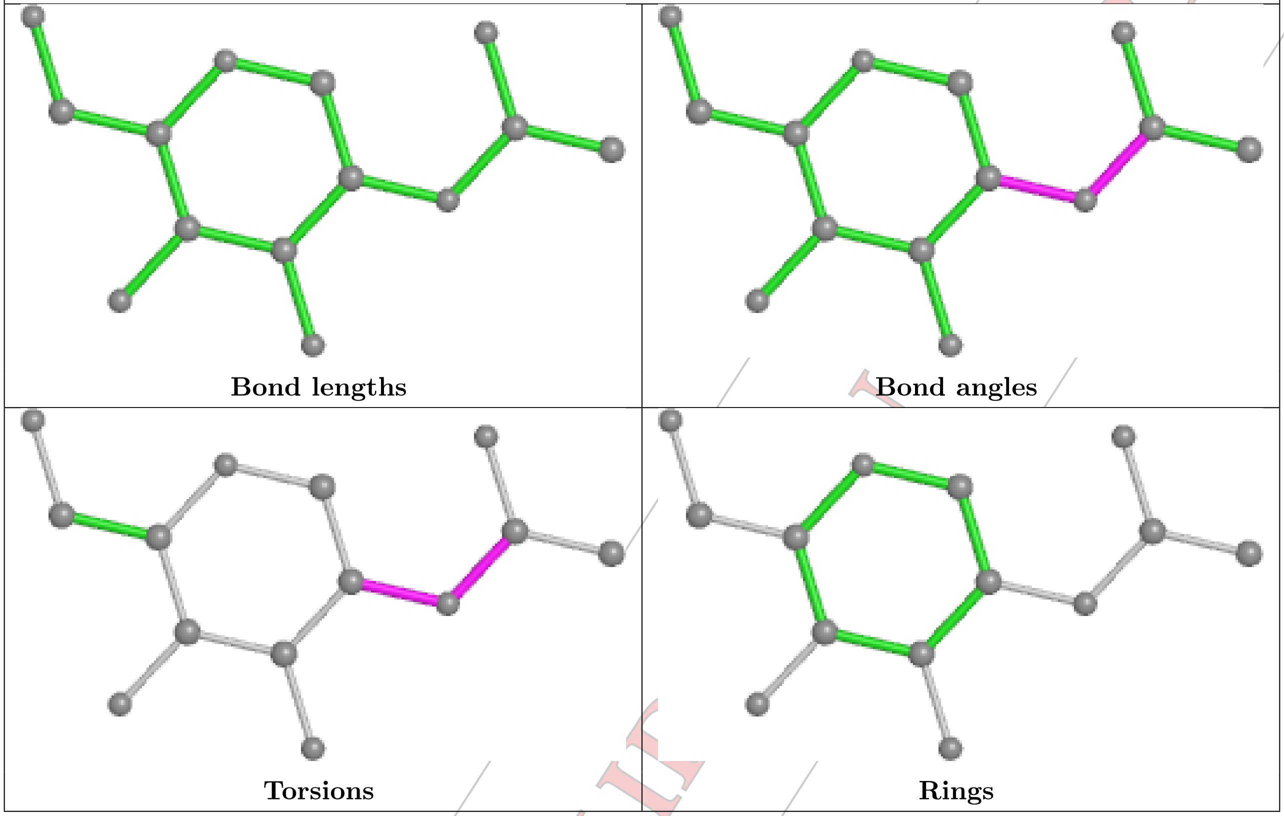

Ligand NAG C 1303

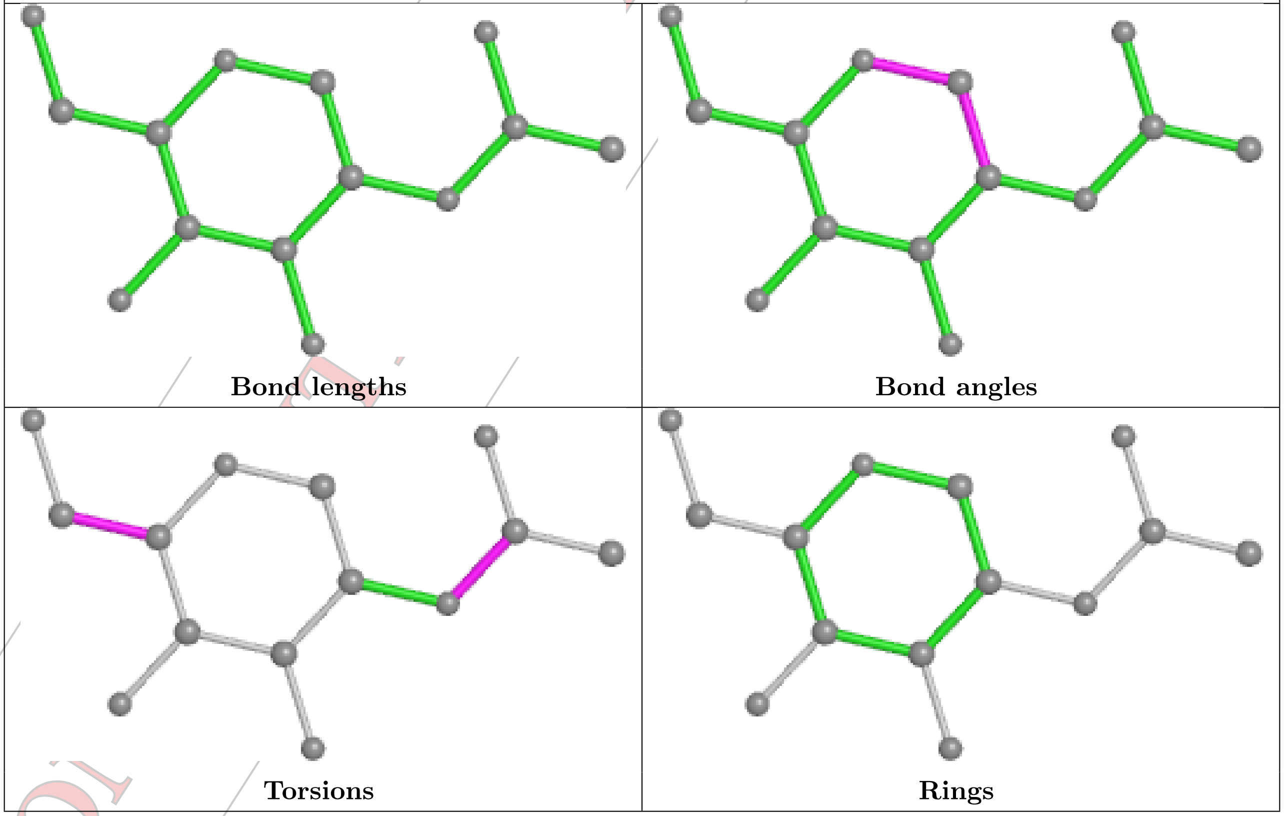

Ligand NAG B 1303

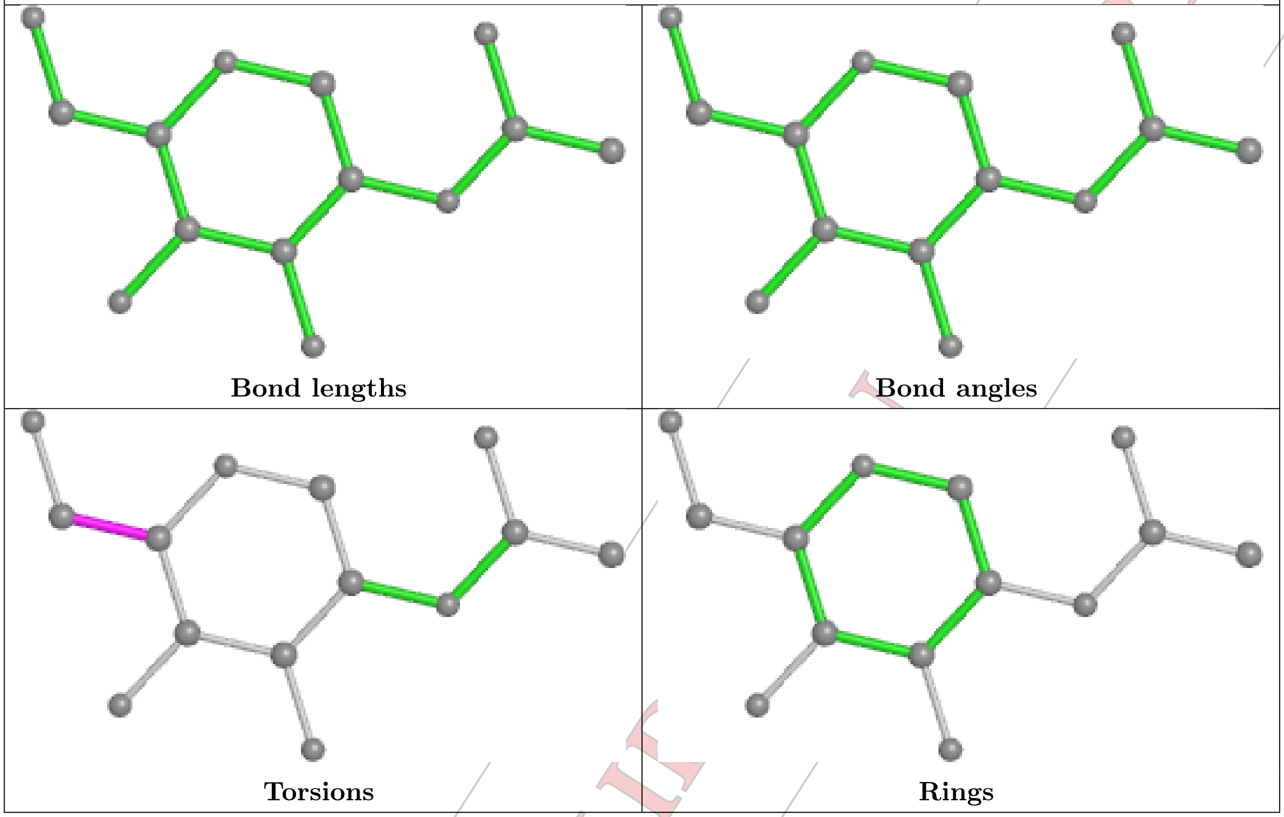

Ligand NAG A 1302

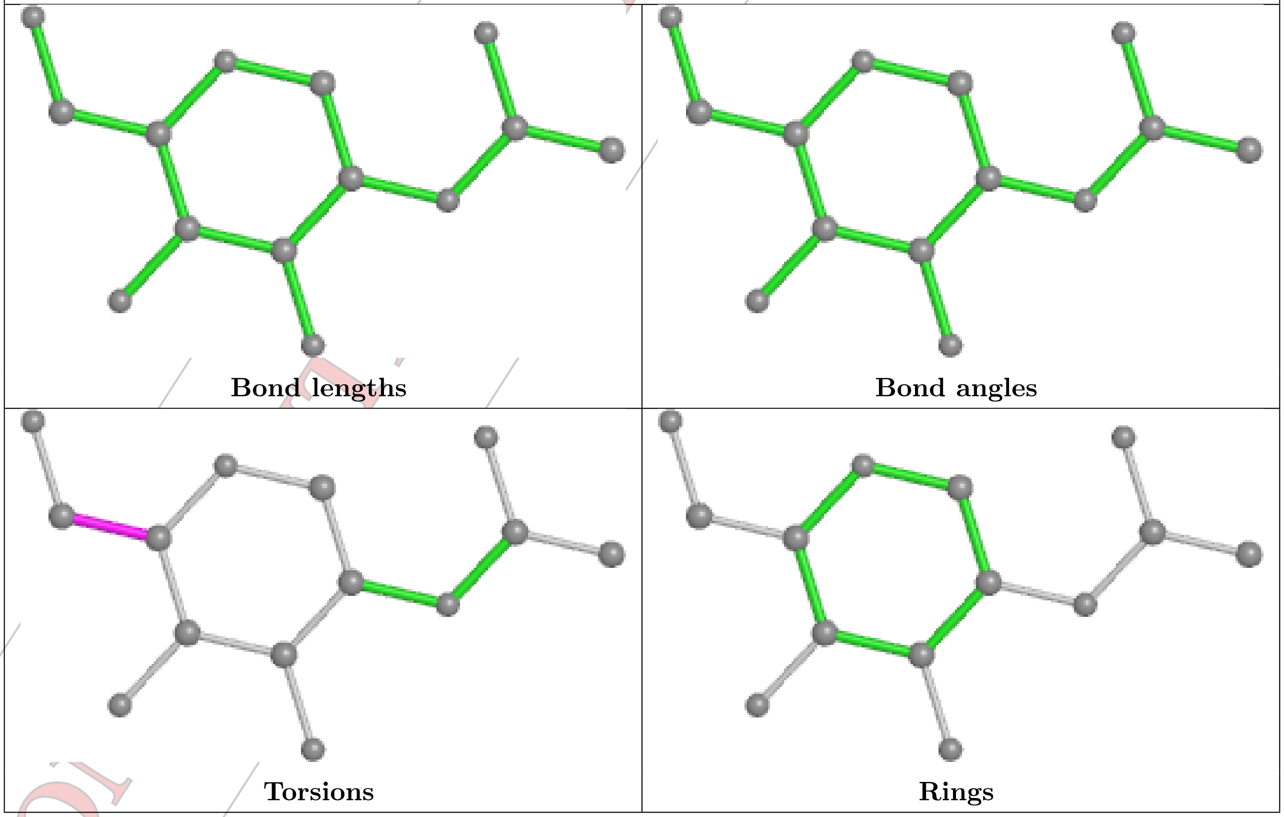

Ligand NAG C 1305

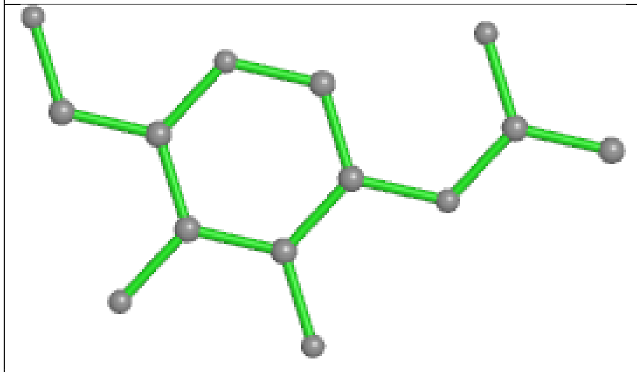

Bond lengths

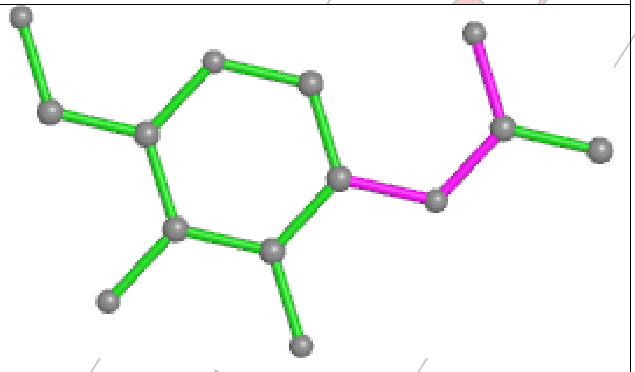

Bond angles

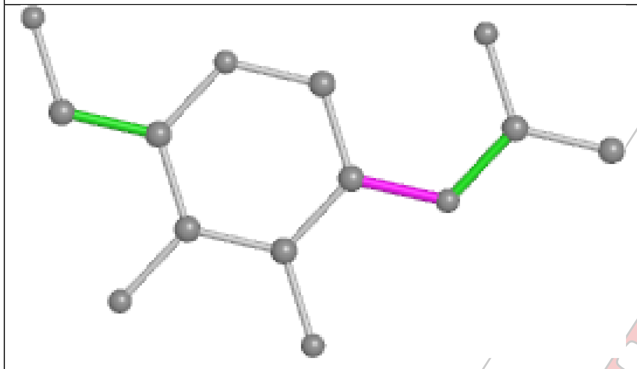

Torsions

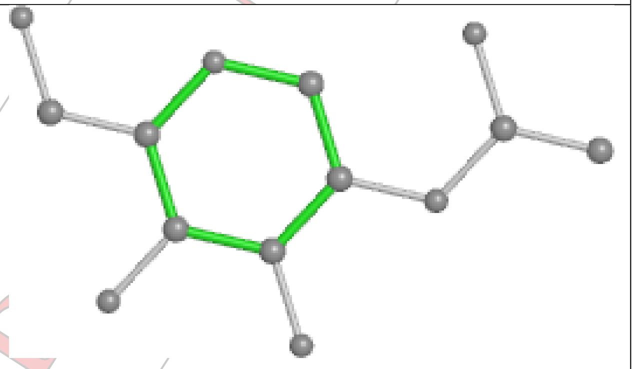

Rings

Ligand NAG C 1302

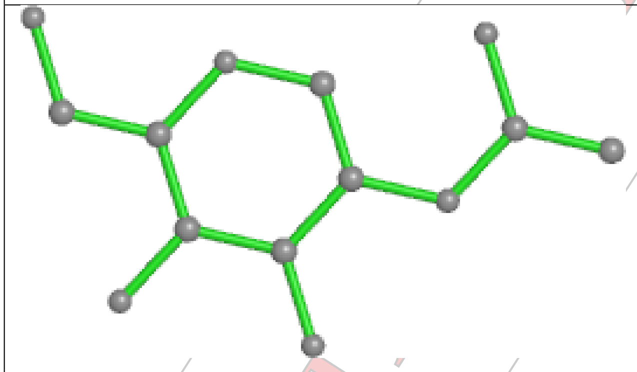

Bond lengths

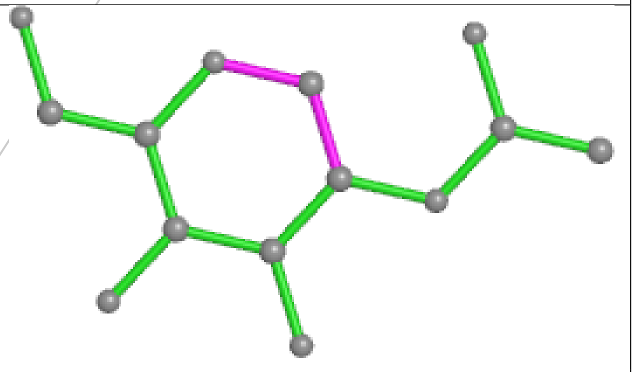

Bond angles

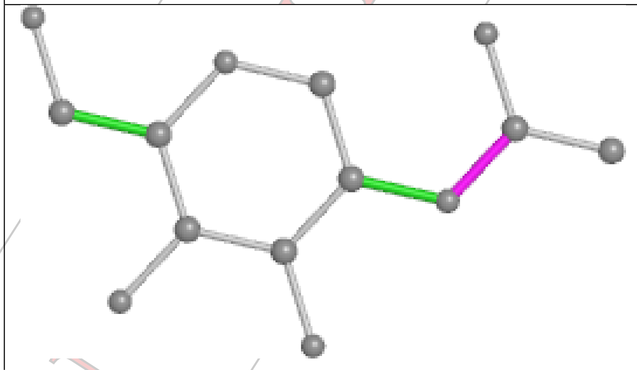

Torsions

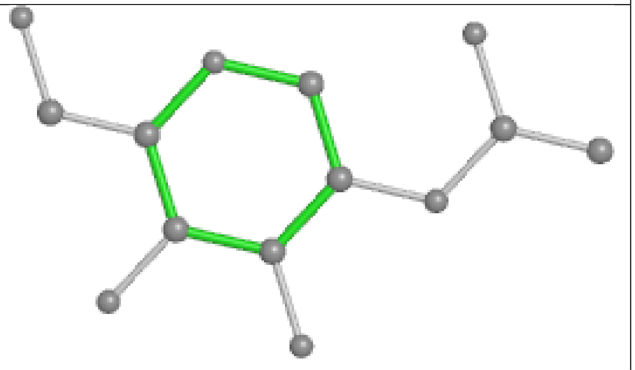

Rings

Ligand NAG A 1304

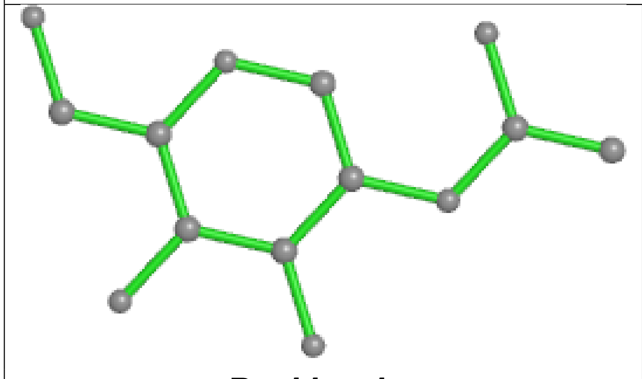

Bond lengths

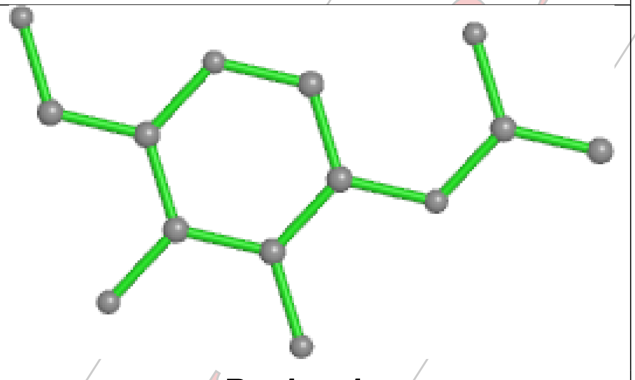

Bond angles

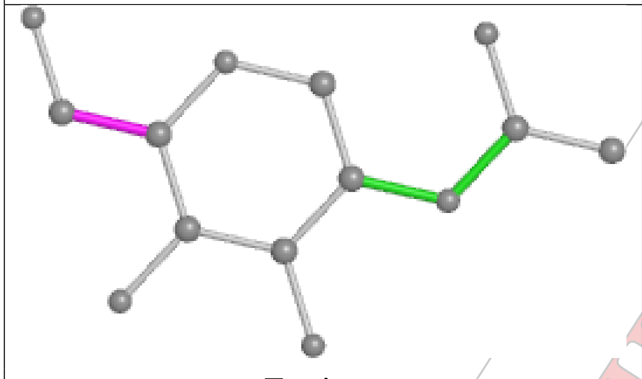

Torsions

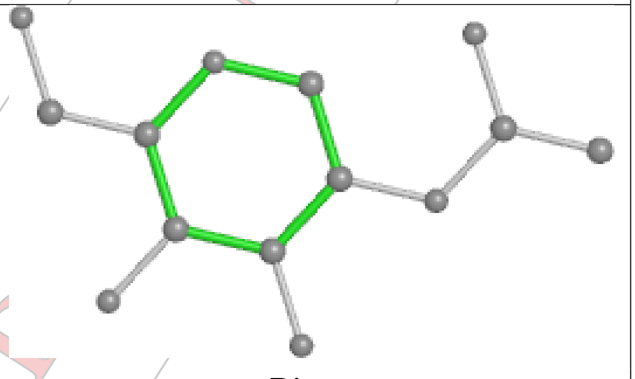

Rings

Ligand NAG C 1306

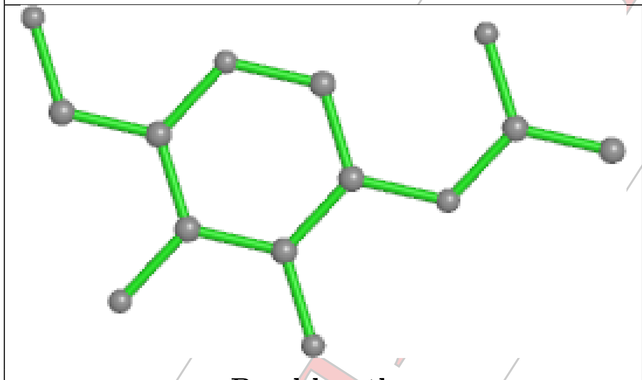

Bond lengths

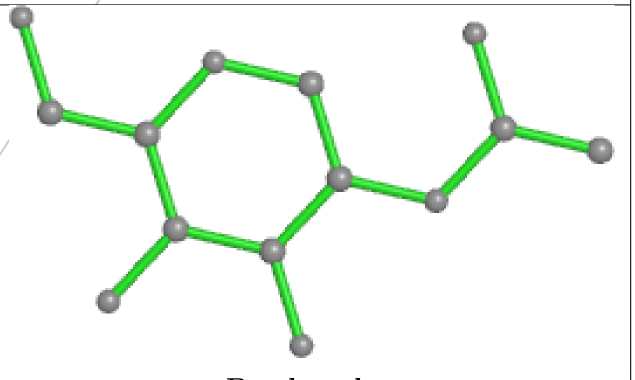

Bond angles

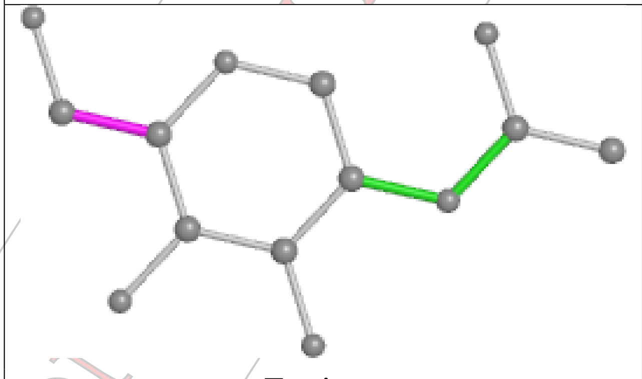

Torsions

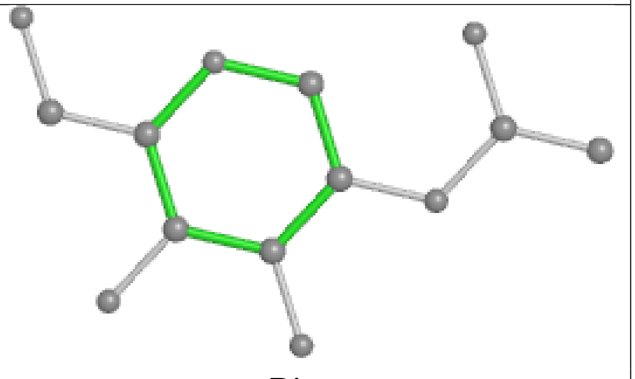

Rings

Ligand NAG B 1301

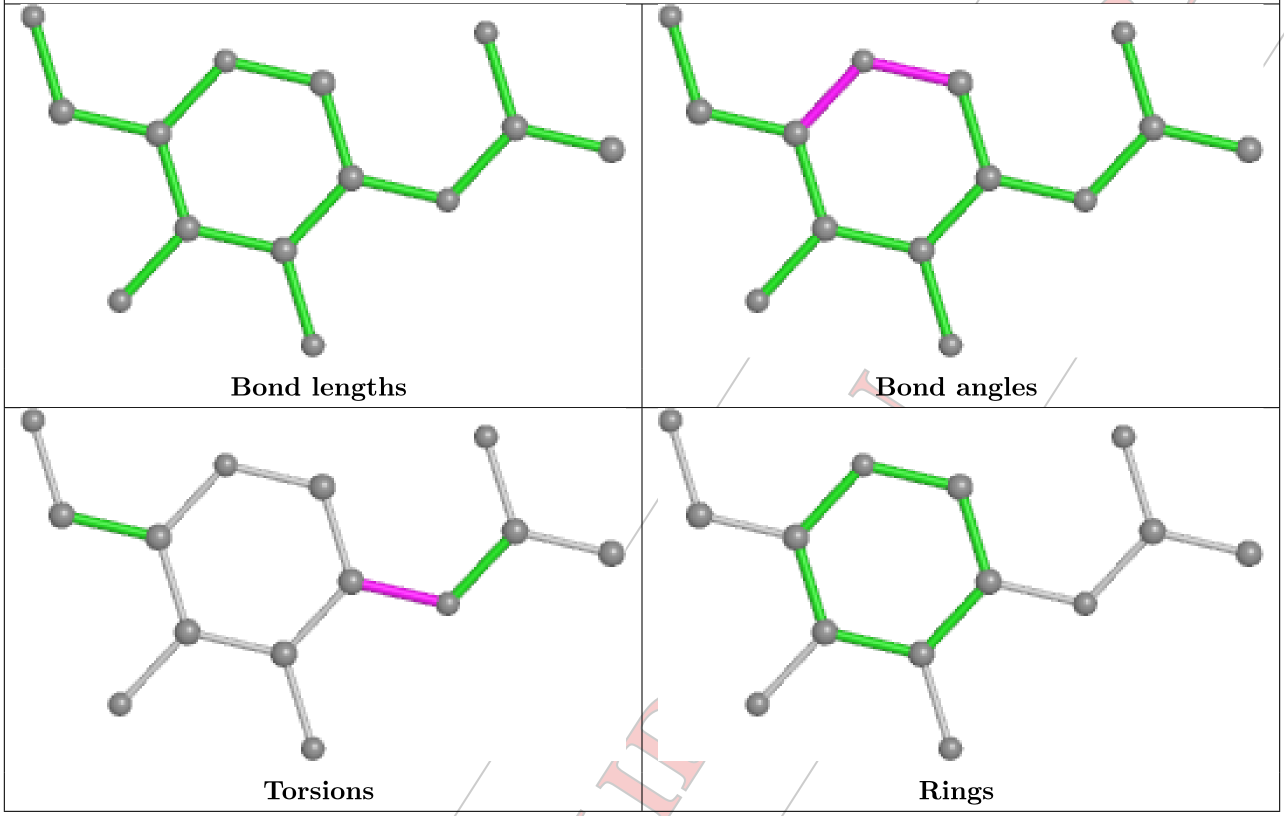

Ligand NAG C 1304

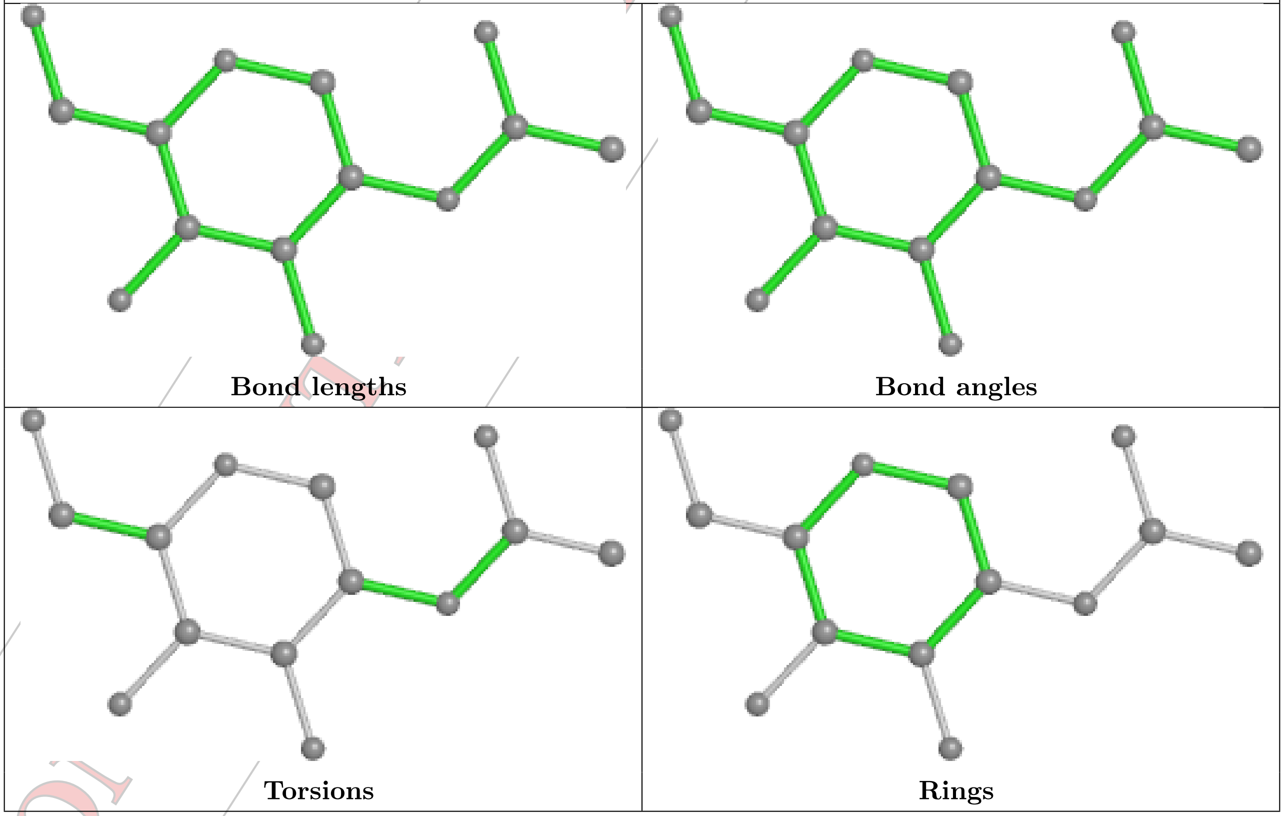

Ligand NAG C 1308

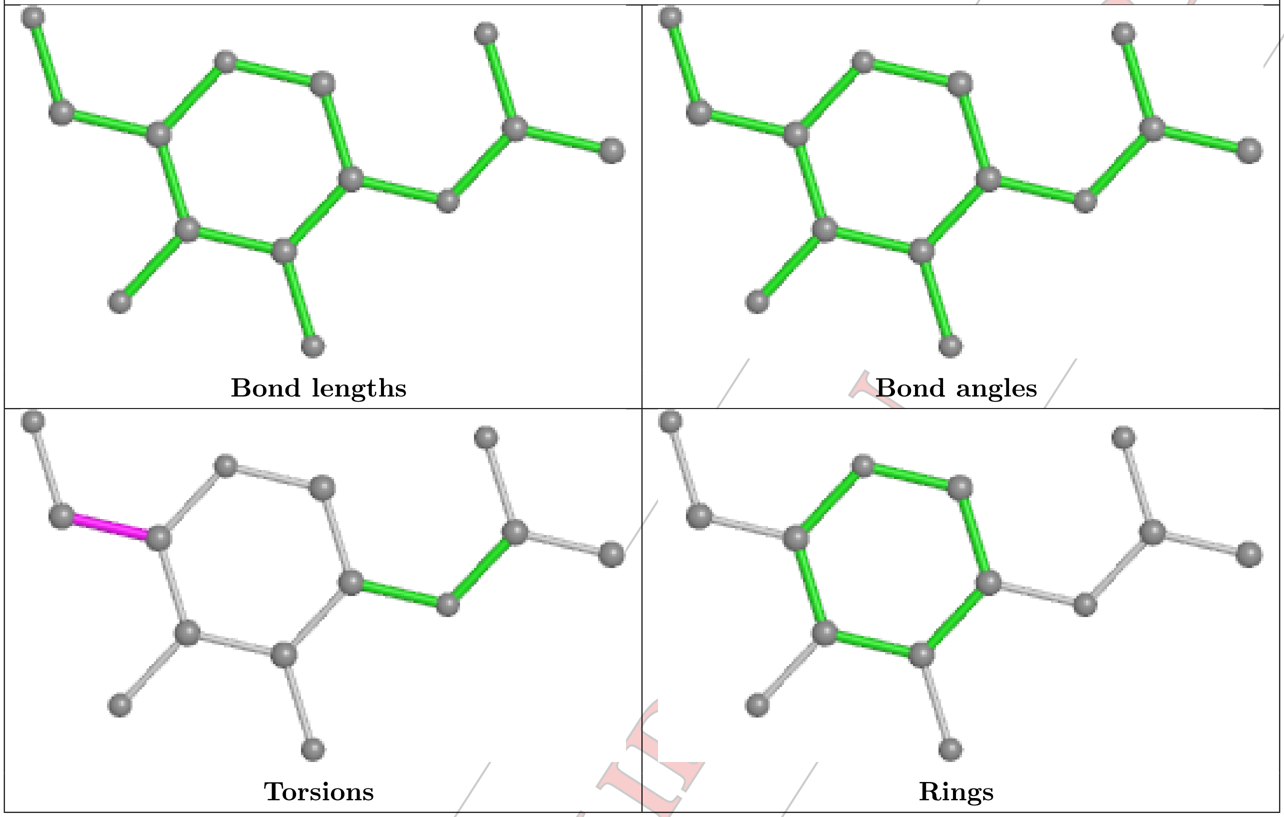

Ligand NAG A 1308

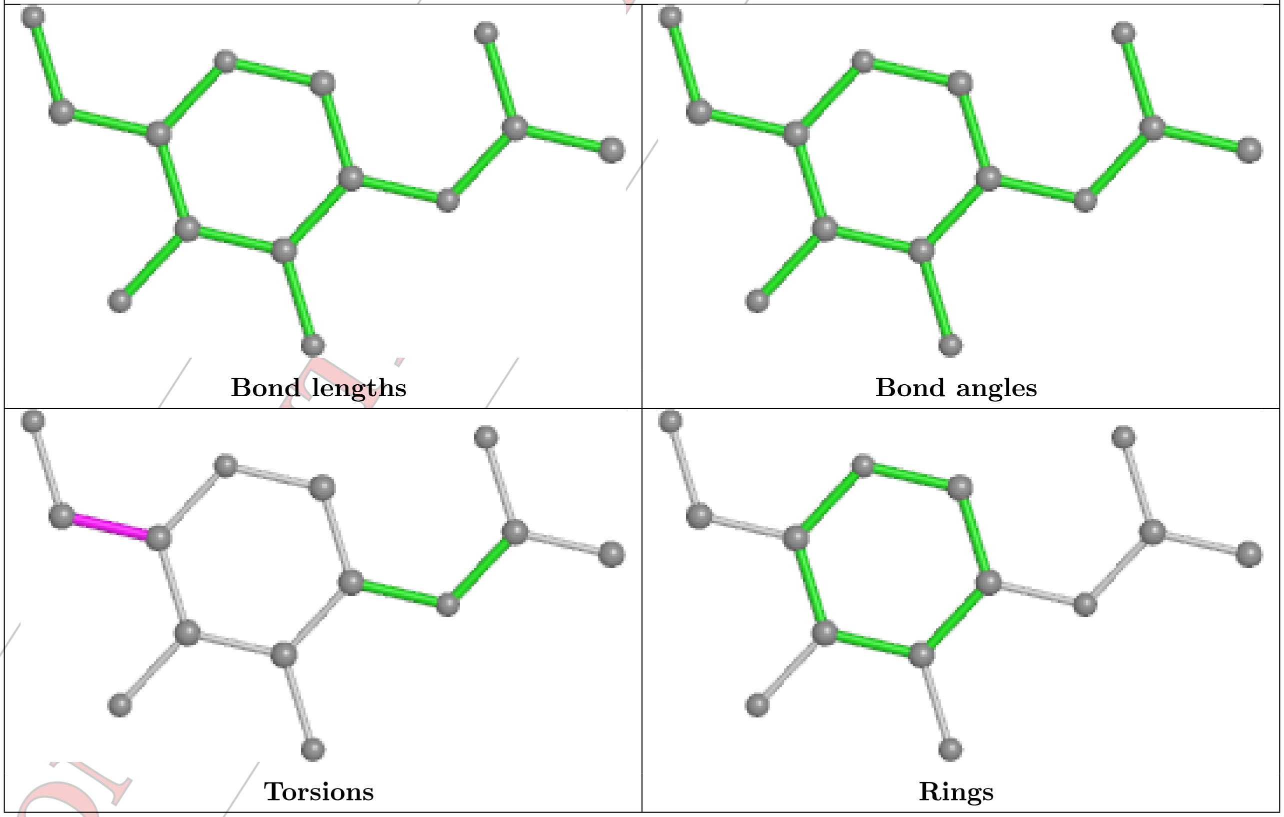

5.7 Other polymers (i)

There are no such residues in this entry.

5.8 Polymer linkage issues (i)

There are no chain breaks in this entry.

CONFIDENTIAL VALIDATION REPORT

6 Map visualisation (i)

This section contains visualisations of the EMDB entry EMD-30982. These allow visual inspection of the internal detail of the map and identification of artifacts.

No raw map or half-maps were deposited for this entry and therefore no images, graphs, etc. pertaining to the raw map can be shown.

6.1 Orthogonal projections (i)

6.1.1 Primary map

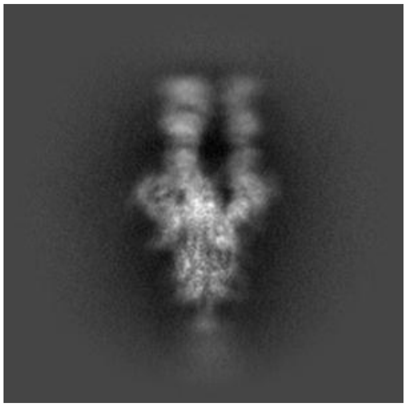

X

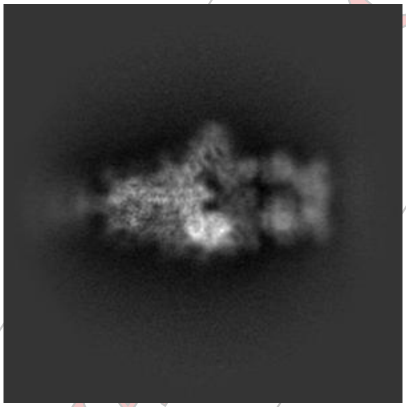

Y

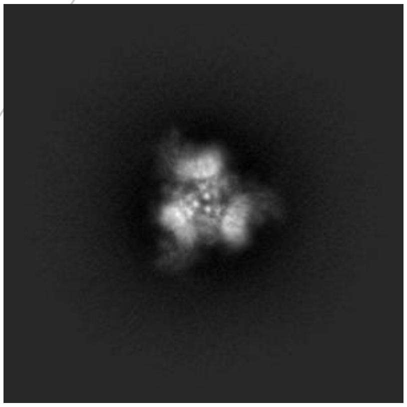

Z

The images above show the map projected in three orthogonal directions.

6.2 Central slices (i)

6.2.1 Primary map

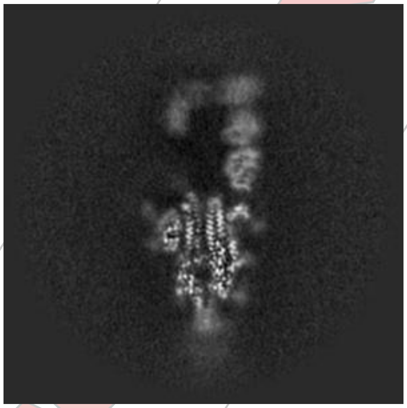

X Index: 256

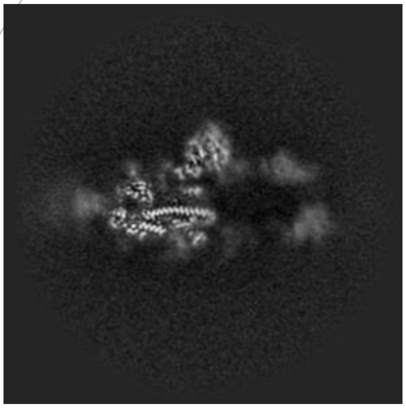

Y Index: 256

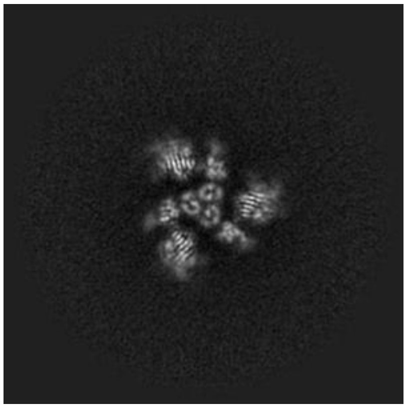

Z Index: 256

CONFIDENTIAL VALIDATION REPORT

The images above show central slices of the map in three orthogonal directions.

6.3 Largest variance slices (i)

6.3.1 Primary map

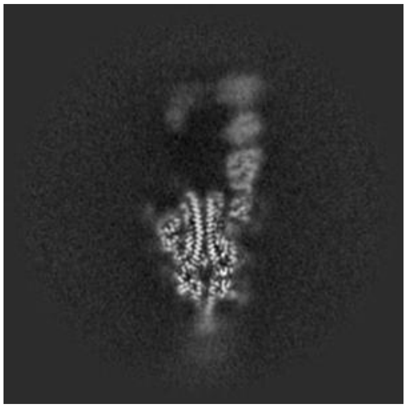
X Index: 261

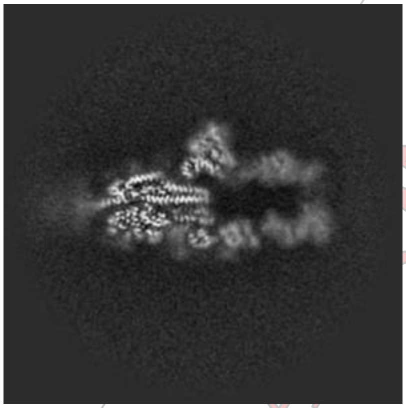
Y Index: 248

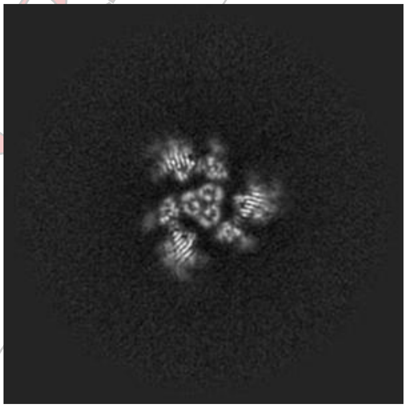
Z Index: 254

The images above show the largest variance slices of the map in three orthogonal directions.

6.4 Orthogonal surface views (i)

6.4.1 Primary map

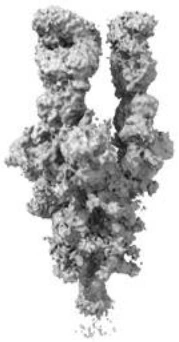
X

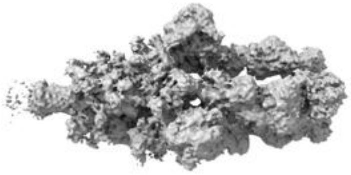
Y

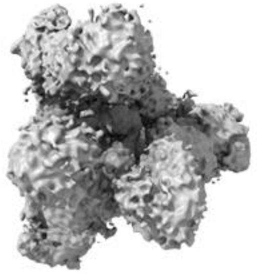
Z

The images above show the 3D surface view of the map at the recommended contour level 0.1042. These images, in conjunction with the slice images, may facilitate assessment of whether an appropriate contour level has been provided.

CONFIDENTIAL VALIDATION REPORT

6.5 Mask visualisation (i)

This section was not generated. No masks/segmentation were deposited.

CONFIDENTIAL VALIDATION REPORT

7 Map analysis (i)

This section contains the results of statistical analysis of the map.

7.1 Map-value distribution (i)

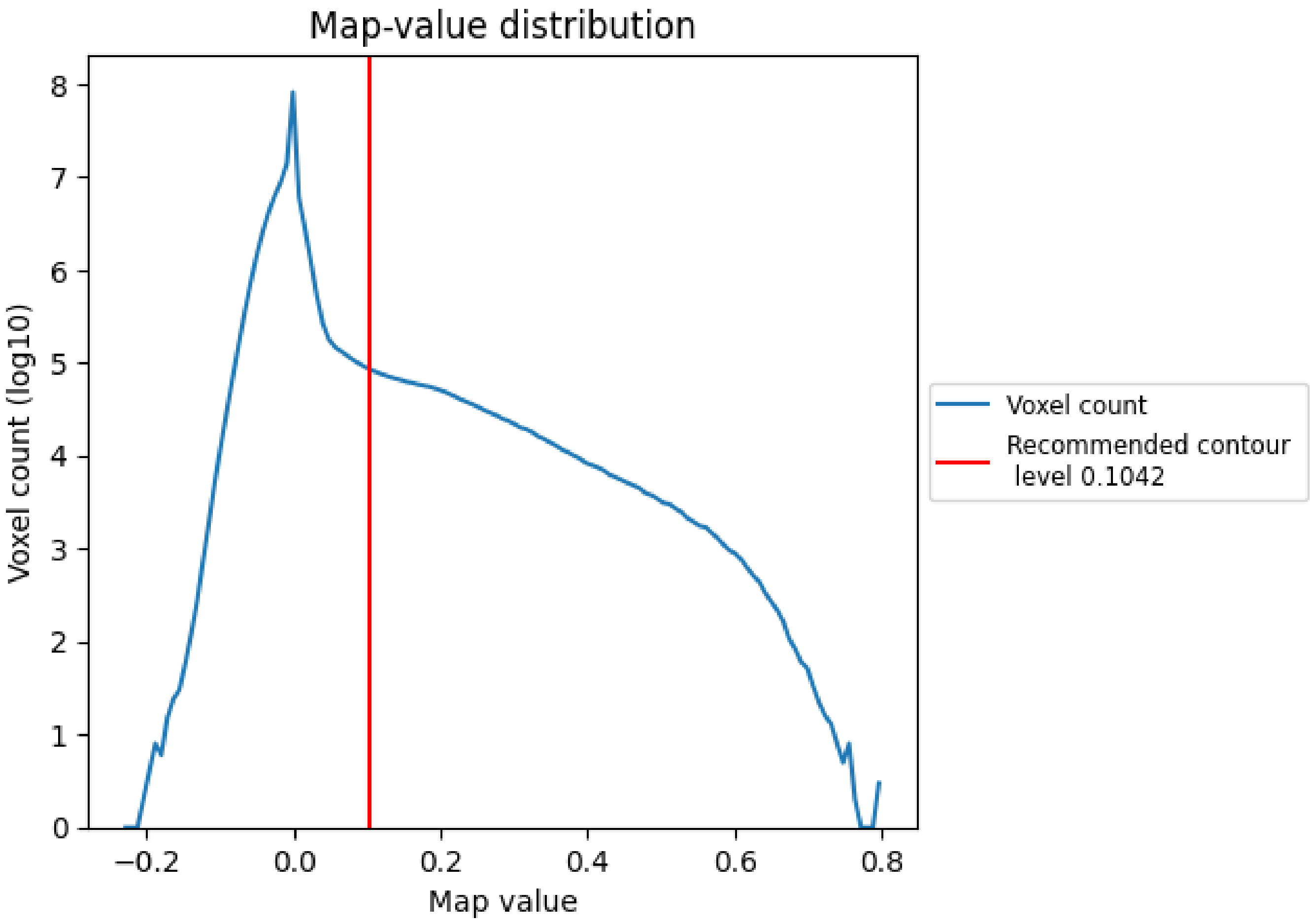

The map-value distribution is plotted in 128 intervals along the x-axis. The y-axis is logarithmic. A spike in this graph at zero usually indicates that the volume has been masked.

CONFIDENTIAL REPORT

7.2 Volume estimate ⓘ

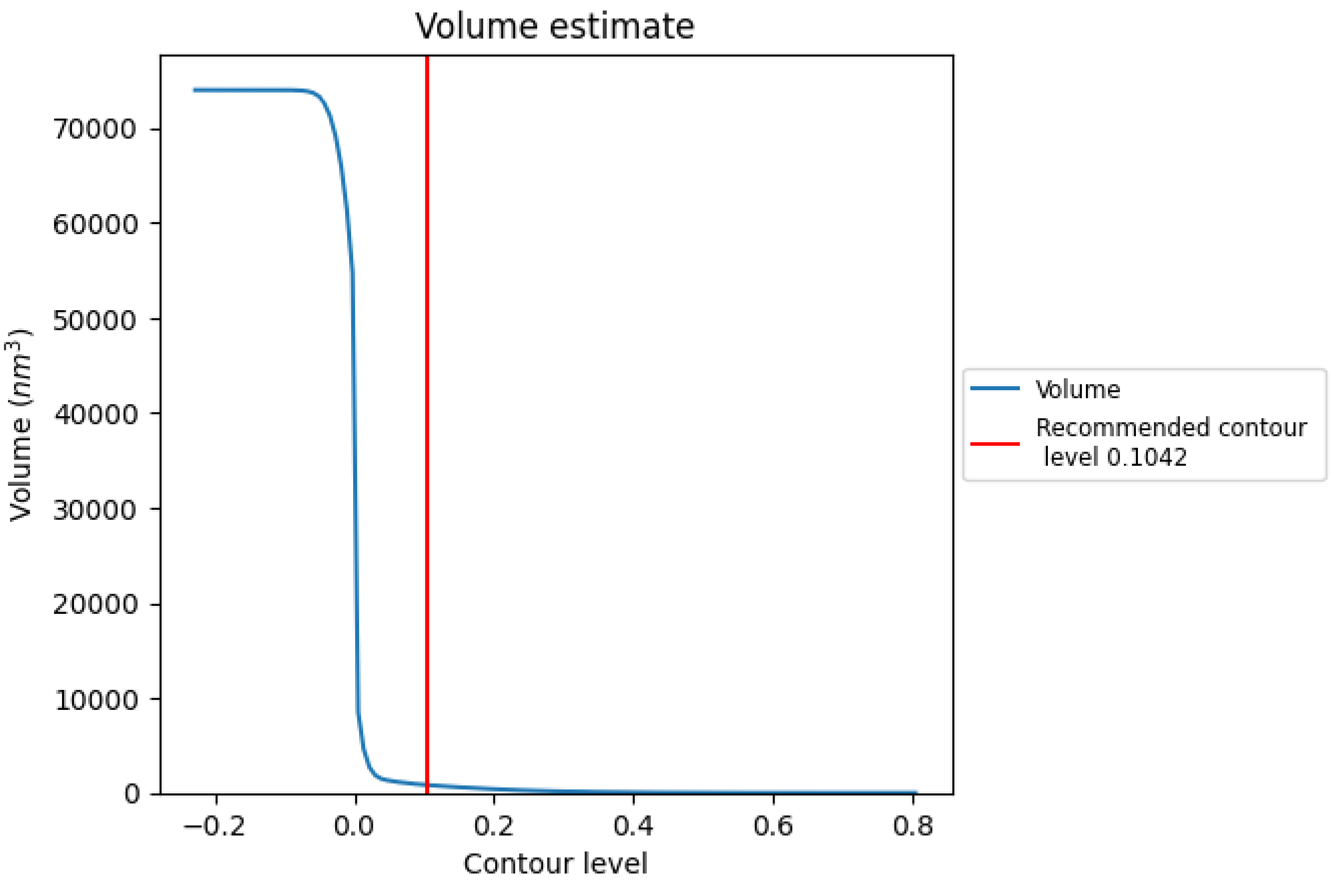

The volume at the recommended contour level is 829 nm^3; this corresponds to an approximate mass of 749 kDa.

The volume estimate graph shows how the enclosed volume varies with the contour level. The recommended contour level is shown as a vertical line and the intersection between the line and the curve gives the volume of the enclosed surface at the given level.

CONFIDENTIAL REPORT

7.3 Rotationally averaged power spectrum (i)

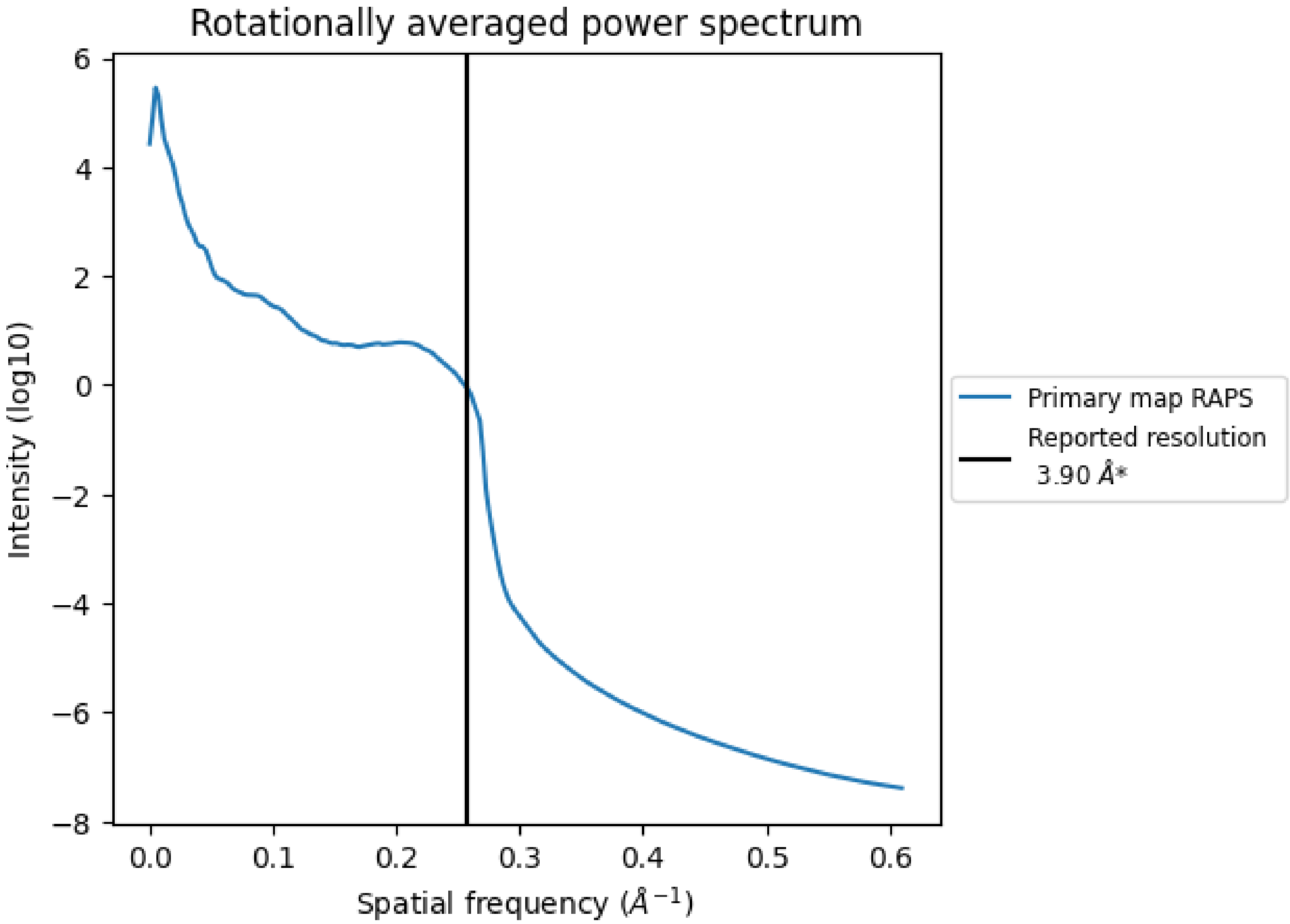

*Reported resolution corresponds to spatial frequency of 0.256 Å^{-1}

CONFIDENTIAL REPORT

8 Fourier-Shell correlation (i)

This section was not generated. No FSC curve or half-maps provided.

CONFIDENTIAL VALIDATION REPORT

9 Map-model fit (i)

This section contains information regarding the fit between EMDB map EMD-30982 and PDB model 7E3K. Per-residue inclusion information can be found in section 3 on page 13.

9.1 Map-model overlay (i)

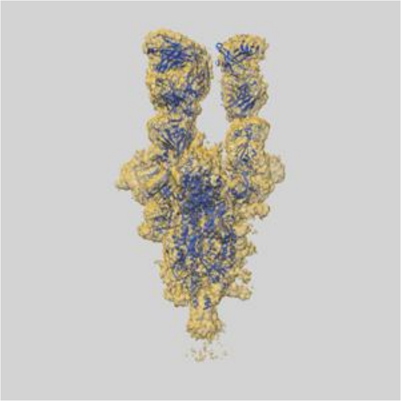

X

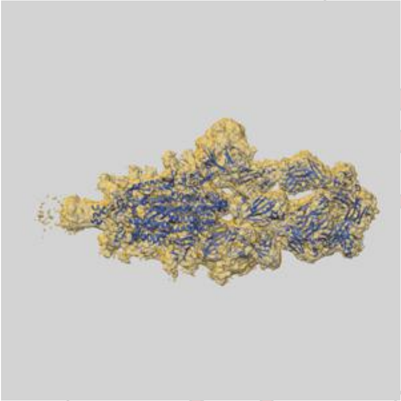

Y

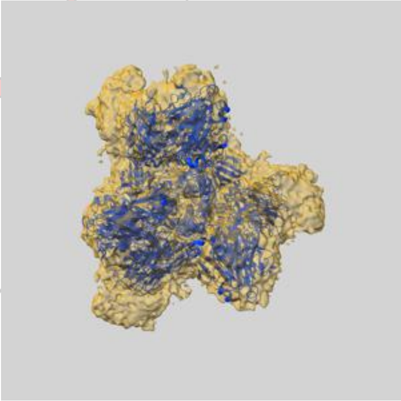

Z

The images above show the 3D surface view of the map at the recommended contour level 0.1042 at 50% transparency in yellow overlaid with a ribbon representation of the model coloured in blue. These images allow for the visual assessment of the quality of fit between the atomic model and the map.

CONFIDENTIAL VALIDATION REPORT

9.2 Atom inclusion ⓘ

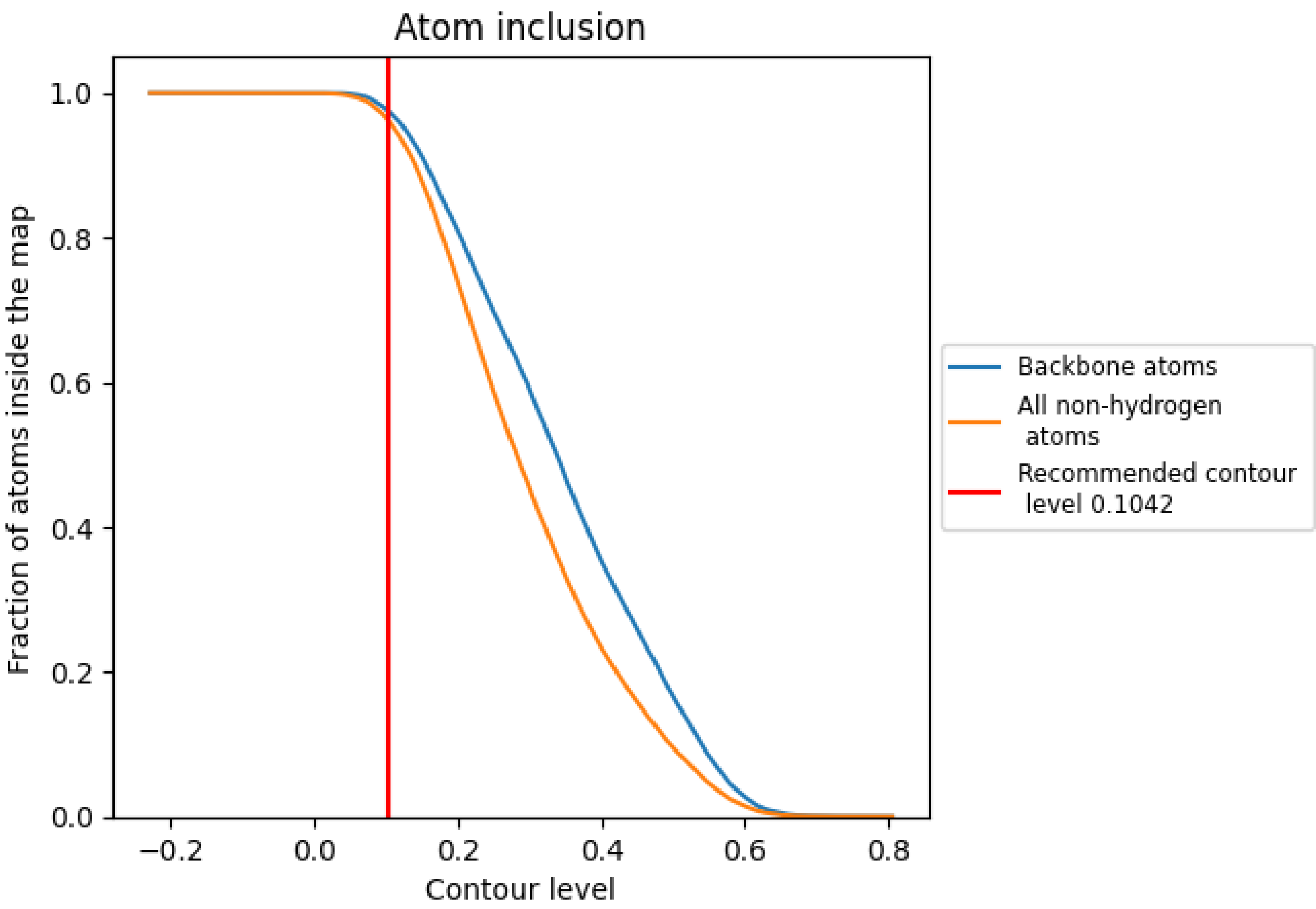

At the recommended contour level, 97% of all backbone atoms, 96% of all non-hydrogen atoms, are inside the map.

CONFIDENTIAL REPORT
